# Mind body exercise improves cognitive function more than aerobic- and resistance exercise in healthy adults aged 55 years and older – an umbrella review

**DOI:** 10.1186/s11556-023-00325-4

**Published:** 2023-08-09

**Authors:** Peter Blomstrand, Dario Tesan, Elisabeth Mueller Nylander, Nerrolyn Ramstrand

**Affiliations:** 1 Department of Natural Sciences and Biomedicine, School of Health and Welfare, Jönköping University, Jönköping, Sweden; 2 Futurum Academy for Health and Care, Region Jönköping County, Jönköping, Sweden; 3 Department of Health, Medicine and Caring Sciences, Linköping University, Linköping, Sweden; 4 Jönköping University Library, Jönköping University, Jönköping, Sweden; 5 Department of Rehabilitation, School of Health and Welfare, Jönköping University, Jönköping, Sweden

**Keywords:** Cognitive function, Exercise, Older adults, Umbrella review, Meta-analysis

## Abstract

Exercise is often cited as a major factor contributing to improved cognitive functioning. As a result, the relationship between exercise and cognition has received much attention in scholarly literature. Systematic reviews and meta-analyses present varying and sometimes conflicting results about the extent to which exercise can influence cognition. The aim of this umbrella review was to summarize the effects of physical exercise on cognitive functions (global cognition, executive function, memory, attention, or processing speed) in healthy adults ≥ 55 years of age.

**Methods** An umbrella review of systematic reviews with meta-analyses investigating the effect of exercise on cognition was performed. Databases (CINAHL, Cochrane Library, MEDLINE, PsycInfo, Scopus, and Web of Science) were searched from inception until June 2023 for reviews of randomized or non-randomised controlled trials. Full-text articles meeting the inclusion criteria were reviewed and methodological quality assessed. Overlap within included reviews was assessed using the corrected covered area method (CCA). A random effects model was used to calculate overall pooled effect size with sub-analyses for specific cognitive domains, exercise type and timing of exercise.

**Results** Database searches identified 9227 reviews. A total of 20 met the inclusion criteria. They were based on 332 original primary studies. Overall quality of the reviews was considered moderate with most meeting 8 or more of the 16 AMSTAR 2 categories. Overall pooled effects indicated that exercise in general has a small positive effect on cognition (d = 0.22; SE = 0.04; *p* < 0.01). Mind–body exercise had the greatest effect with a pooled effect size of (d = 0.48; SE = 0.06; *p* < 0.001). Exercise had a moderate positive effect on global cognition (d = 0.43; SE = 0,11; *p* < 0,001) and a small positive effect on executive function, memory, attention, and processing speed. Chronic exercise was more effective than acute exercise. Variation across studies due to heterogeneity was considered very high.

**Conclusions** Mind–body exercise has moderate positive effects on the cognitive function of people aged 55 or older. To promote healthy aging, mind–body exercise should be used over a prolonged period to complement other types of exercise. Results of this review should be used to inform the development of guidelines to promote healthy aging.

**Trial registration **PROSPERO (CDR 42022312955).

**Supplementary Information:**

The online version contains supplementary material available at 10.1186/s11556-023-00325-4.

## Background

An active lifestyle has long been promoted as a means of slowing down the aging process and helping people retain their independence. Physical exercise in particular has been identified as beneficial for older adults and has been suggested to have positive effects on both physical and cognitive health outcomes [1]. While there is high-level evidence supporting exercise as an effective intervention for maintaining physical function in older adults [2], recent research has provided reason to question previous claims of a positive association between physical exercise and cognitive functioning [3].

Cognitive functioning can be analysed from a general perspective (global cognition) or sub-divided into specific domains, each representing different abilities. These include executive functions, memory, attention, and processing speed [4]. Each of these domains has been associated with a measurable decline with age [5] which begins before the age of 60 in healthy adults [6]. Murman [5] suggests that the greatest impact of age-related change in cognition results from deterioration in a person’s ability of perform cognitive tasks requiring rapid processing of information and then a decision. These types of tasks require effective use of working memory, processing speed, and executive functions.

Slowing or even reversing age related cognitive decline has been a popular topic of many scholarly publications and physical exercise is one intervention that has received much attention as a potential mediating factor. Studies to date have attempted to identify the most effective type of exercise to promote maintenance of cognitive functions [7–9], determine the optimal intensity, duration and frequency of exercise for promoting cognitive function [8, 10–12] and to identify which specific cognitive domains may benefit most from an exercise intervention [13]. Specific types of physical exercise that have been investigated can be loosely categorised into three groups; aerobic exercise (e.g. walking, running, dancing, swimming or bicycling), resistance exercise (e.g. weight training, training by use of body weight or elastic bands) and mind body exercise (e.g. yoga, tai chi or qi gong) [7–9]. The link between exercise and cognition has also been studied as an acute intervention, involving a single bout of training, and as a chronic intervention, consisting of multiple bouts of training performed over a period of weeks or months [14].

A recent meta-analysis comparing the effects of resistance and aerobic exercise on global cognition, memory and executive function concluded that both types of exercise were beneficial for older adults with and without cognitive decline [15]. Another recent systematic review by Huang et al. showed that resistance exercise had the highest probability for slowing down cognitive decline [16]. Zhang et al. reported that mind–body exercise has significant benefits for global cognition, executive functions, learning and memory [17]. In contrast to these findings, a recent umbrella review including 23 meta-analyses and including people between the ages of 6 and 80 showed only small exercise related benefits on cognition and demonstrated that these effects became negligible after correcting for publication bias [3].

Many physiological processes are stimulated by exercise and support the premise that increased physical activity contributes to maintenance or even improvements in cognitive health. These processes are generally related to an exercise induced increase in neural activity or increased levels of exerkines. For example, high intensity aerobic exercise has been associated with increased activity in the frontal and parietal cortices as well as the supplementary motor area [18], all key areas for executive functions and motor planning. Aerobic exercise but not resistance exercise has also been linked to an increase in resting concentrations of brain-derived neutrophic factor (BDNF) in peripheral blood [19], and hippocampus [20], a regions which plays a major role in learning and memory. BDNF expression has also been found to be affected by the duration and intensity of exercise [19, 21].

Recent data has also linked potential beneficial effects of exercise to crosstalk which takes place between the brain and the liver, muscle, adipose tissue and gut [22]. In these studies, exercise-related signalling molecules and exerkines have been identified to regulate the positive effects of exercise on cognitive function. An example of this is Cathepsin B which increases in plasma and muscles during exercise and which is strongly associated with memory functions [23]. Similarly, Glycosylposphatidylinositol-Specific Phospholipase D1 (GLDP1) from the liver is increased after exercise. GLDP1 is correlated with neurogenesis, increased expression of BDNF and improved hippocampal dependent learning and memory in aged mice [24]. Exercise also increases circulating interleukin-6 (IL-6) which reduces the pathological amyloid precursor protein in prefrontal cortex and hippocampus. This protein plays a central role in the pathophysiology Alzheimer´s disease [25].

While pathophysiological evidence seems to support exercise induced benefits on cognition, inconsistencies in data syntheses which have studied cognitive outcomes after exercise suggest that further investigation is warranted. Umbrella reviews are a relatively new concept which may help to shed light on uncertainties that exist regarding the relationship between exercise and cognition. This research method allows researchers to synthesise results from previous reviews under a single “umbrella” and to draw conclusions about the overall strength and quality of studies which may have inconsistent of conflicting conclusions [26]. Umbrella reviews represent one of the highest levels of evidence [27].

The aim of this study was to conduct an umbrella review to evaluate the impact of physical exercise on cognitive functions in healthy adults who are 55 years of age or older. More specifically we aimed to determine the type of exercise that is most effective for improving cognitive functions (aerobic exercise, resistance exercise or mind body exercise), which cognitive domains are likely to be most affected (global cognition, executive functions, memory, attention, or processing speed) and if exercise duration (acute versus chronic) has a significant effect on cognitive outcomes.

## Methods

### Protocol and registration

The protocol for this umbrella review was pre-registered in PROSPERO and is available at https://www.crd.york.ac.uk/prospero/display_record.php?RecordID=42022312955. This review complies with the Preferred Reporting Items for Systematic reviews and Meta-Analyses (PRISMA [28]).

### Literature search strategy

In March 2022 and June 2023, the following databases were searched for systematic reviews with meta-analyses of randomized controlled trials (RCTs) or non-randomized controlled trials (NRCTs): CINAHL (EBSCOhost), Cochrane Library (Wiley), MEDLINE (EBSCOhost), PsycInfo (ProQuest), Scopus, and Web of Science. The search strategies were based on the concepts of age (older adults), exercise, and cognition. Searches were further limited by study type but not by language or publication date. The full search strategy for each database is reported in Supplementary data, S1. A manual search of the reference lists of included reviews was performed in addition to the digital search to ensure that no relevant articles were missed.

### The literature selection criteria

Studies were included in this umbrella review if they were systematic reviews with meta-analyses which assessed the effect of acute or chronic exercise interventions on cognitive functions. The definition of systematic review used in the study was: “A review of a clearly formulated question that uses systematic and explicit methods to identify, select, and critically appraise relevant research, and to collect and analyse data from the studies that are included in the review” [29]. Participants were required to be ≥ 55 years and healthy, with no specific disorders such as cancer, heart failure, mental illness, neurological disease, cognitive impairment, or dementia. The age cut-off of 55 years deviates from the original Prospero registration and was made for pragmatic reasons as few reviews were found to include participants from 65 years of age. Reviews that comprised of both healthy and unhealthy participants were included only if results from the healthy participants were reported independently and meta data for this specific group could be extracted. Reviews were required to investigate a physical exercise intervention compared to a control group performing no activity or another type of activity. Physical exercise interventions included in this umbrella review were required to be categorised as either: aerobic exercise, resistance exercise, mind body exercise or a combination of these. These categorisations were selected as they represent the broad classifications commonly used by health promoting organisations and have previously been used to classify exercise types in systematic reviews [30, 31]. For the purposes of the review, aerobic exercise was defined as any exercise intervention aiming to improve cardiovascular fitness. This included activities such as walking, running, dancing, bicycling, swimming, or exergaming [7]. Resistance exercise was defined as interventions which aimed to improve muscle strength and included weight training, bodyweight training or use of resistance bands [8]. Mind–body exercise was classified as exercise which combines movement sequences, breathing control, and attention regulation [32]. Examples of mind–body exercise are Tai Chi, Pilates and Yoga.

In addition to an exercise intervention, meta-analyses included in the umbrella review were required to investigate at least one cognitive outcome that could be classified into one or more of the following categories: global cognition, executive functioning, memory, attention, or processing speed. Only peer reviewed, English language publications were included. No supplemental primary studies were added.

### Study selection and data extraction

Publications identified by the search were exported to EndNote where duplicate publications were removed using methods described by Bramer et al. [33]. In contrast to other de-duplication methods, this method does not rely on digital object identifies (DOI’s) and PubMedIDs (PMIDs) which are not present in every database, rather combines other fields (e.g. author, year, title) with page numbers to identify duplicate publications. Following the deduplication process remaining publications were exported to Rayyan online software where titles and abstracts were initially reviewed [34]. Publications were excluded if they were not systematic reviews with meta-analyses, if they included participants under 55 years of age in the analysis or if they included patients with cognitive impairment, dementia, or severe medical disorders and did not present separate analyses for healthy people. The reviewers (PB, NR, DT) worked in pairs to review titles and abstract. Each pair initially reviewed the studies independently before results were compared to the second reviewer. Any disagreement was resolved through discussion with the third reviewer. Finally, the reviewers read the full text of remaining articles. Manuscripts were excluded during the full text review if they had; A. the wrong study design (e.g. not a systematic review or meta-analysis); B. wrong or no intervention; C. wrong outcome (e.g. no cognitive test reported); D. wrong participants (e.g. participants with mild cognitive impairment or dementia, or aged < 55 years); or E. were not published in English. During this process reviewers read the full text of each article independently before comparing their decision to include or exclude the review with at least one other author. Conflicts were discussed among all three authors until consensus was reached.

Data extracted from the remaining articles included citation (author/year), study design, population characteristics, description of the exercise intervention, cognitive outcome measures used, and results of the study (effect size, confidence intervals). At least two authors independently extracted all the data and then met to compare their results. Discrepancies were resolved through discussion among all three authors.

### Study quality assessment

The validated AMSTAR tool for systematic reviews was used to assess the risk of bias and the quality of reviews [35, 36]. Risk of bias was initially rated independently by all three authors. Ratings were then compared between the authors and any conflicts were resolved through discussion within the group. To assess the potential impact of overlap, where the same primary studies were included in two or more reviews, we used the corrected cover area (CCA) method. This is a validated measure which uses a citation matrix to calculate overlapping publications included in reviews. A CCA score of 0–5 indicates slight overlap, 6–10 moderate, 11–15 high and > 15very high [37]. The authors agreed that reviews would be removed from the analysis if the overlap was found to be high or very high.

### Statistical analysis

Data analysis was performed with IBM SPSS Statistics v. 28.0.1.0. Pooled effect sizes were calculated from effect size data reported in each review (Cohen’s d) together with standard error data calculated from 95% confidence intervals [38]. Four studies included in this umbrella review reported effect size as Hedge’s g [10, 39–41]. The main difference between Cohen´s d and Hedge´s g is that Hedge´s g is multiplied by a correction factor for small samples. Given that the sample sizes in studies reporting Hedge´s g were relatively large, and considering that Hedge´s g would provide a more conservative estimate, this data was not converted to Cohen´s d [42]. No re-analysis of raw data from reviews included in this study was performed.

When available, data was extracted to allow for a sub-analysis of a/ global cognition and specific cognitive domains; b/ different types of exercise and c/ acute versus chronic exercise. Specific domains were included in sub-analyses when they were identified in at least two reviews. Cognition was analysed as global cognition or one of the following specific domains; executive function, memory, attention and processing speed.

Data related to the specific type of exercise performed was classified as being aerobic, resistance or mind–body exercise. Classifications were based on the definitions presented above and agreed upon by all three authors. Classifications of acute versus chronic exercise were determine in the same manner.

Data was pooled into one overall effect size for each analysis. A random effects model was used to adjust the weights according to the extent of variation, or heterogeneity. Effect sizes were interpreted as small d = 0.2; medium d = 0.5 and large d = 0.8 [38].

Publication bias and small study effects biases were evaluated using funnel plots and Egger’s test. Small-study effects bias was considered an issue for *p* values < 0.01 in the regression asymmetry test [43]. Heterogeneity was estimated using I^2^ and interpreted as very large (> 75%); large (50–74); moderate (25–49); and low (< 25%) [44]. In both instances *p* < 0.05 was considered significant. To explore if results related to the overall effect size were sensitive to exclusion of specific studies, we calculated effect size while systematically excluding one study at a time.

## Results

**Fig. 1:**
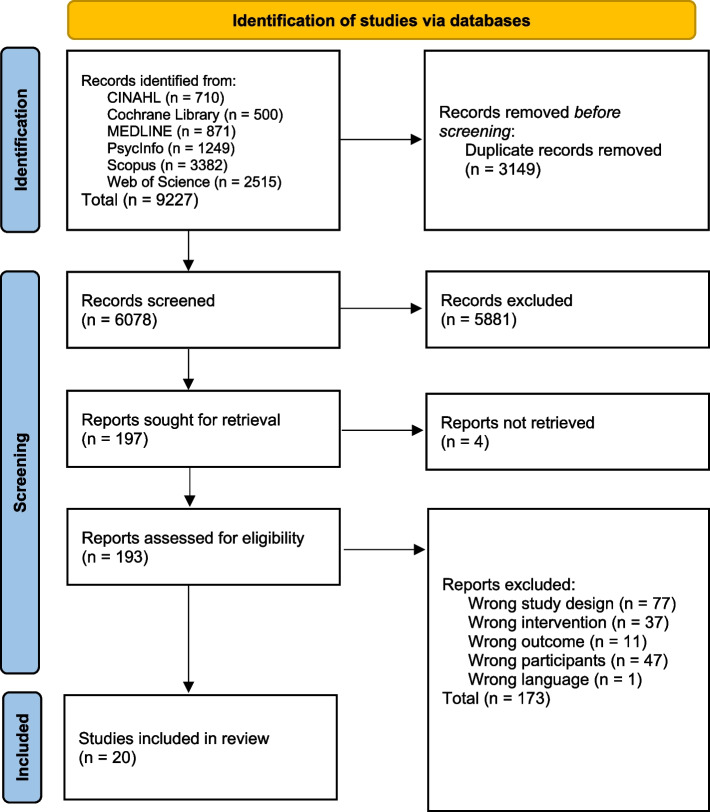
PRISMA flow diagram. Legends Flow chart illustrating the literature search

Database searches identified 9227 reviews. No additional reviews were identified by manually searching reference lists. 3149 reviews were removed as they were identified to be duplicate publications, and 5881 reviews were removed following the authors’ review of titles and abstracts. Full text copies of four reviews were not able to be retrieved. Full text versions 193 articles were read by the authors, of which 173 were excluded due to; wrong study design (*n* = 77); wrong intervention (*n* = 37); wrong outcome (*n* = 11); wrong participants (*n* = 47); wrong language (*n* = 1). This left a total of 20 meta-analyses that were identified as assessing the effects of exercise on cognition in healthy individuals aged 55 years and older. Figure 1 presents the PRISMA flowchart and reasons for exclusion. A list of all articles excluded during the full-text review is included as Supplementary data, S2 (Fig. 1).

### Characteristics of included studies

Study characteristics are presented as Table 1. The average number of studies included in each meta-analysis ranged from two (45) to 50 (41) with an average of 13 studies. Overlap in the included reviews is presented in supplementary data, S3. The CCA was calculated to be 1.84% representing only slight overlap [37].

**Table 1:** Characteristics of included studies

Author, year, reference, title	Type of review	No. relevant studies / total no. studies	No. relevant participant / total no. participants, age and cognitive status of participants	Physical activity / exercise description	Control group activity	Outcome cognitive domain	Main results of the Healthy persons without cognitive impairment
Angevaren et al., 2008 [7]* *Physical activity and enhanced fitness to improve cognitive function in older people without known cognitive impairment*	MA of RCTs	11/11	MD/612 > 55 years, without MCI	Aerobic exercise Exercise programs of any intensity, duration, frequency, and length	Any intervention, No intervention, Flexibility, Balance, Resistance, Social activities Mental activities	Attention, Executive function, Memory, Processing speed	Aerobic exercise had a large effect on cognitive function, especially motor function, and a moderate effect on auditory attention Small effects were found on cognitive speed and visual attention
Bhattacharyya et al., 2021 [9]* *Effects of yoga-related mind–body therapies on cognitive function in older adults: a systematic review with meta-analysis*	MA of RCTs NRCTs	6/12	673/912 > 55 years, with and without MCI	Mind body exercise Duration 60 min, Frequency1-4/week, Length 2–6 months	Health education, Memory enhancement training, Stretching, Resistance, Music listening	Executive function, Memory, Processing speed	Mind–body exercises (Kundalini and Hatha yoga and yogic meditation) had a small positive effect on executive function, memory, and attention and processing speed
Chen et al., 2020 [10]* *Effects of exercise training interventions on executive function in older adults: a systematic review and meta-analysis*	MA of RCTs	13/24	1989/7023 > 55 years with or without MCI	Aerobic exercise Resistance exercise Mind body exercise Intensity 3–9 METs, Duration 45–60 min, Frequency 2—7/week, Length 1- 6 months	No activity, Stretching, Social activity, Flexibility	Executive function	Exercise training was associated with a small improvement in executive function, especially inhibition, especially updating and shifting
Clifford et al., 2022 [45]* *The effect of dance on physical health and cognition in community dwelling older adults: A systematic review and meta-analysis*	MA of RCTs	5/22	400/1090 > 60 years without MCI	Aerobic exercise Freq 1–3/week Length 6 weeks to 18 months	No activity Other exercise	Global cognition, Memory	The effect of dance on cognition was not significantly different compared to other exercise interventions
Coelho-Junior et al., 2022 [8]* *Resistance training improves cognitive function in older adults with different cognitive status: a systematic review and meta-analysis*	MA of RCTs	11/18	383/MD ≥ 60 years Healthy participants with or without MCI	Resistance exercise Intensity low–high, Frequency 1–3/week, Length 6–36 weeks	No intervention, Stretching, Balance, Social activities	Attention, Global cognitive function, Memory,	Resistance training had a moderate effect in improving overall global cognitive function and a small effect on short term memory. No improvement was seen regarding concentration and attention
Falck et al., 2019 [40]* *Impact of exercise training on physical and cognitive function among older adults: a systematic review and meta-analysis*	MA of RCTs	32/48	3523/6281 ≥ 60 years without MCI	Aerobic Resistance Mixed Exercise Frequency ≥ 1/week, Length ≥ 2 months	MD	Executive function, Global cognitive function, Memory, Processing speed	Exercise training had a small positive effect on cognitive function
Gasquoine and Chen, 2022 [41]* *Effect of physical exercise on popular measures of executive function in older, nonclinical, participants of randomized controlled trials: a meta-analytic review*	MA of RCTs	50/50	MD > 60 years Nonclinical participants	Aerobic Resistance Mixed Duration 1.5–3 h, Length 12–104 weeks	Placebo, Waitlist, No exercise, Stretching, Health lectures, Educational lectures, Balance	Executive function, Memory, Processing speed	Exercise training had only a small positive effect on executive functions (digit symbol). Memory tests were all not significantly different from zero
Hindin and Zelinski, 2012 [46] *Extended practice and aerobic exercise interventions benefit untrained cognitive outcomes in older adults: a meta-analysis*	MA of NRCTs	17/42	1016/3781 > 55 years without MCI	Aerobic exercise Duration 3–79 min, Length 2–52 weeks	Extended practice of cognitive tasks	Choice reaction time, Executive function, Memory	Aerobic fitness training produced a small improvement in executive function, choice reaction time and memory
Jiang, et al. 2022 [47]* *Effects of exergaming on executive function of older adults: a systematic review and meta-analysis*	MA of RCTs	11/15	489/650 > 60 Without MCI	Aerobic exercise Freq 1–3/week Length single session (one study)-26 weeks	No activity Bike Balance/stretching Education material Aerobic Cognitive training Exercise without VR	Executive function	Participants who were subjected to an exergaming intervention had better overall EF than control subjects
Loprinzi et al., 2019 [48]* *The temporal effects of acute exercise on episodic memory function: systematic review with meta-analysis*	MA of SR	2/25	68/2085 middle-age 45–60 years and older adults > 60 years	Aerobic exercise Intensity low – vigorous, Duration 2–35 min	MD	Memory	Acute aerobic exercise before memory encoding and during early consolidation had a negative effect on episodic memory
Ma et al., 2023 [13]* *The effect of rhythmic movement on physical and cognitive functions among cognitively healthy older adults: A systematic review and meta-analysis*	MA of RCTs	10/44	1358/2752 ≥ 60 years without MCI	Aerobic exercise Freq 1–3/ week Length 8 weeks—4 years	No activity, health education, walking, wait-listing	Global cognition, Executive function, memory, attention	An association was found between rhythmic movement and global cognitive function. No significant improvement was found in executive function
Martins et al., 2022 [49]* *The Effects of High-Speed Resistance Training on Health Outcomes in Independent Older Adults: A Systematic Review* *and Meta-Analysis*	MA of RCTs	4/14	133/408 ≥ 65 years without MCI	High-speed resistance training Freq 1–3/weeks Length 18–16 weeks	MD	Global cognition	High-speed resistance training had large effects on global cognitive function
Roig et al., 2013 [14]* *The effects of cardiovascular exercise on human memory: a review with meta-analysis*	MA of RCTs NRCTs	14/50	1244/2224 ≥ 60 years without MCI	Aerobic exercise Intensity low—vigorous, Duration 20–40 min, Length 1–60 months	No exercise	Memory	Acute aerobic exercise had a large effect and long-term exercise an insignificant effect on long-term memory Long-term exercise had an insignificant effect on short-term memory
Scherder et al., 2014 [50]* *Executive functions of sedentary elderly may benefit from walking: a systematic review and meta-analysis*	MA of RCTs	5/8	363/642 > 55 years, with or without MCI	Aerobic exercise Intensity low – moderate, Frequency 3–5/week, Duration 40–60 min, Length 1–12 months	No intervention, Flexibility, Toning, Balance, Strength	Executive function	Walking had a small positive effect on executive functions, set-shifting and inhibition, in older persons without cognitive impairment. Walking had no effect on executive function in older persons with cognitive impairment
Wang et al., 2021 [51]* *Effects of square-stepping exercise on motor and cognitive function in older adults—a systematic review and meta-analysis*	MA of RCTs NRCTs	5/10	308/423 ≥ 55 years without significant medical conditions	Aerobic exercise Frequency 3–7/week, Duration 30–60 min, Length 1.5–12 months	Maintained lifestyle, Outdoor supervised walking, Daily activities, Aerobic exercise Balance	Executive function, Global cognitive function	Square-stepping exercise had no effect on reaction time or executive function
Xiong et al., 2021 [39]* *Effects of physical exercise on executive function in cognitively healthy older adults: a systematic review and meta-analysis of randomized controlled trials: physical exercise for executive function*	MA of RCTs	25/25	3197/3197 ≥ 60 years without MCI	Aerobic exercise Resistance exercise Mind–body exercise Frequency ≥ 3/week, Duration 20–90 min, Length 1–12 months	Flexibility, Balance, Toning, Stretching, Daily routine, Waitlist, Social activities, Reading, Health education	Memory	Physical exercise had a medium effect in improving the executive function subdomain cognitive flexibility but no effect on working memory or inhibitory control
Ye et al., 2021 [32]* *The effect of mind–body exercise on memory in older adults: a systematic review and meta-analysis*	MA of RCTs	6/12	490/1051 ≥ 60 years with or without MCI	Mind body exercise Frequency 3–7/week, Duration 30–90 min, Length 8–48 weeks	Daily routine, Health education, Stretching, Toning, Resistance	Memory	Mind body exercise had a large effect in improving general memory and long-term memory in participants without cognitive impairment. Corresponding effects were moderate on episodic memory, semantic memory and short-term memory. A small effect was seen on working memory
Zhao et al., 2022 [52]* *Physical Activity and Cognition in Sedentary Older Adults: A Systematic* *Review and Meta-Analysis*	MA of RCTs	5/7	280/350 ≥ 60 years with or without MCI	Aerobic exercise Freq 2–4/weeks Length 8–24 weeks	No exercise	Global cognition, Memory, Executive function, Processing speed	Physical activity might have a general positive effect on the cognition of sedentary older adults
Zhidong et al., 2021 [53]* *Effects of physical exercise on working memory in older adults: a systematic and meta-analytic review*	MA of RCTs	17/28	1259/2063 ≥ 60 years with or without MCI	Aerobic exercise Resistance exercise Mind body exercise Mixed Exercise Freq 1–5/week Length 4–52 weeks	No activity social activities, health education, stretching exercises, cognitive training,	Memory	Physical exercise can improve the working memory of older adults. Greatest effects are seen in multi-component exercise or mind–body exercise of moderate intensity for 45–60 min 3 times a week, for more than 6 months
Zhu et al., 2023 [54]* Effects of physical activity on visuospatial working memory in healthy individuals: A systematic review and meta-analysis	MA of RCTs	12/21	867/1595 Children, adults and Seniors without MCI	Aerobic Freq 15 min –5/week Single session—18 weeks	No movement; cognitive training; daily routine; stretching; reading; passive cycling; Walk	Working memory	Physical activity had a small but significant positive impact on VSWM in healthy individuals

Twenty systematic reviews and meta-analyses assessing the effects of exercise on cognitive functions in healthy individuals aged 55 years and older were included in our study. To summarize the effects were eleven of these studies (*) also included in a meta-analysis. *CCTs* Clinical controlled trials, *MA* Meta-analyses, *MCI* mild cognitive impairment, *MD* missing data, *No*. number of, *RCTs* Randomized controlled trials, *NRCTs* not randomized controlled trials, *SMD* Standardized mean difference, *SR* Systematic review, *WMD* weighted mean difference, *WSTs* Within subjects trials

The total number of participants included in meta-analyses ranged from 68 (45) to 3523 (40). Fifteen meta-analyses included only RCTs, three included both RTCs and NRTCs [9, 14, 51], and one included systematic reviews of studies with an experimental design [48, 55]. Most reviews included studies with passive control groups although Clifford et al. [45] and Jiang et al. [47] did include both passive and active control groups. It was not possible to determine the characteristics of control groups in two reviews [40, 49] (Table 1).

Age span of participants included in the reviews varied from 55 to 94 years. Most studies (*n* = 11) investigated the effects of aerobic exercise on cognition [7, 13, 14, 45–48, 50–52, 54]. Three studies investigated the effects of mind body exercise on cognition [9, 32, 39], two analysed the effects of resistance exercise [8, 49] and five investigated the effects of mixed exercise interventions [10, 39–41, 53] (Table 1). Only two studies investigated cognition after a single bout of exercise (Acute) [14, 48] while all others investigated cognition after prolong exercise (Chronic). The duration of chronic exercise ranged from one month [10] to two-years [41]. The most common intervention for control groups was no training, other control interventions included balance training, flexibility training, health education and even social activities.

Outcomes were typically reported for one or more cognitive domains. Six studies reported results for global cognition [8, 13, 40, 49, 51, 52], while others reported outcomes for more specific cognitive domains. Memory and executive function were the most frequently reported domains (15 studies and 11 studies respectively). Processing speed and attention were reported in five and three studies respectively. Ma et al. [13] reported analyses for global cognition and memory but it was unclear if memory data was reported as mean differences or standardised mean differences so only data for global cognition was analysed.

Several meta-analyses chose to report specific domain broken down into sub-categories. An example of this was Angevaren et al. [7] who presented separate analyses for verbal memory, visual memory, working memory and memory functions. Cognitive domains along with cognitive tests used to measure cognition are presented in Supplementary file S4. The most frequently used tests for executive functioning were the Trail making test B and Task switching test. Memory was most frequently evaluated using the Wechsler Memory Scale and Rey’s Auditory test. Many studies used several different tests of memory and over 40 different memory tests were reported across the studies included in this umbrella review.

### Methodological quality assessment

**Fig. 2:**
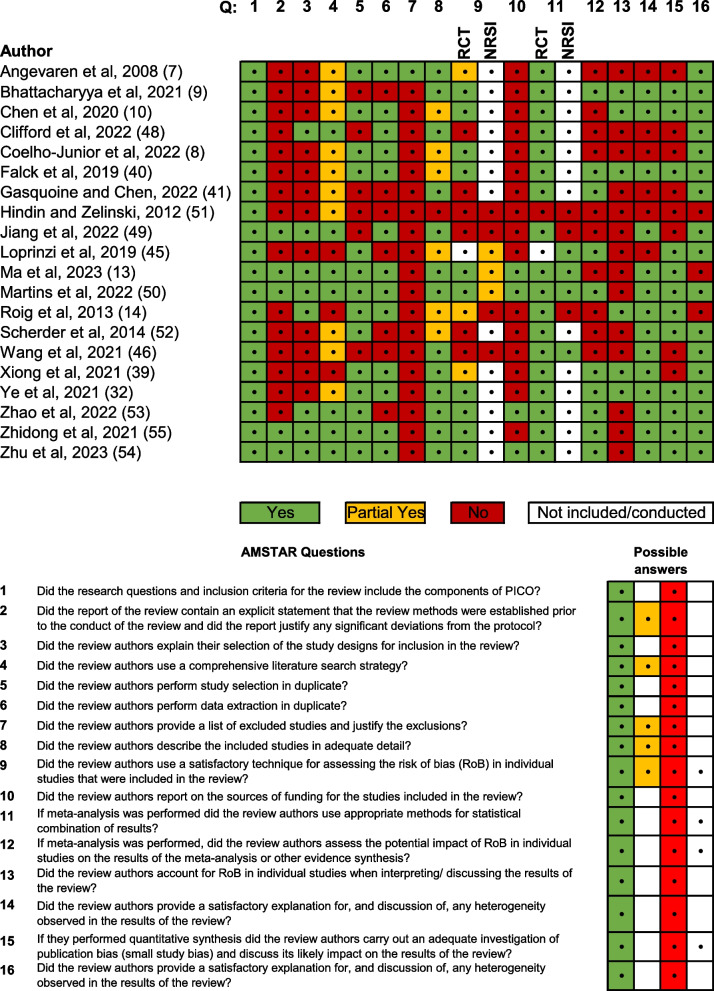
Amstar rating. The validated AMSTAR tool for systematic reviews was used to assess the risk of bias and the quality of reviews. RCT, Randomized controlled trials; NRSI, Not randomized studies of interventions

The AMSTAR 2 rating of overall confidence in reviews is presented in Fig. 2. In the AMSTAR 2 rating overall quality was considered high in six studies, moderate in 12 studies and low in two studies. The review by Hindin et al. was considered to have critical flaws, having scored satisfactorily on only one of the sixteen AMSTAR 2 criteria. This study was removed from further analysis [40]. Five studies contained an explicit statement that the review methods were established prior to the review. Recently published studies presented a fully comprehensive literature search strategy to a greater extent than older studies. No studies reported on sources of funding for articles included in their review. Most authors used appropriate methods for study selection and methods used for meta-analyses were generally performed well (Fig. 2).

### Results from pooling of effect sizes

**Fig. 3:**
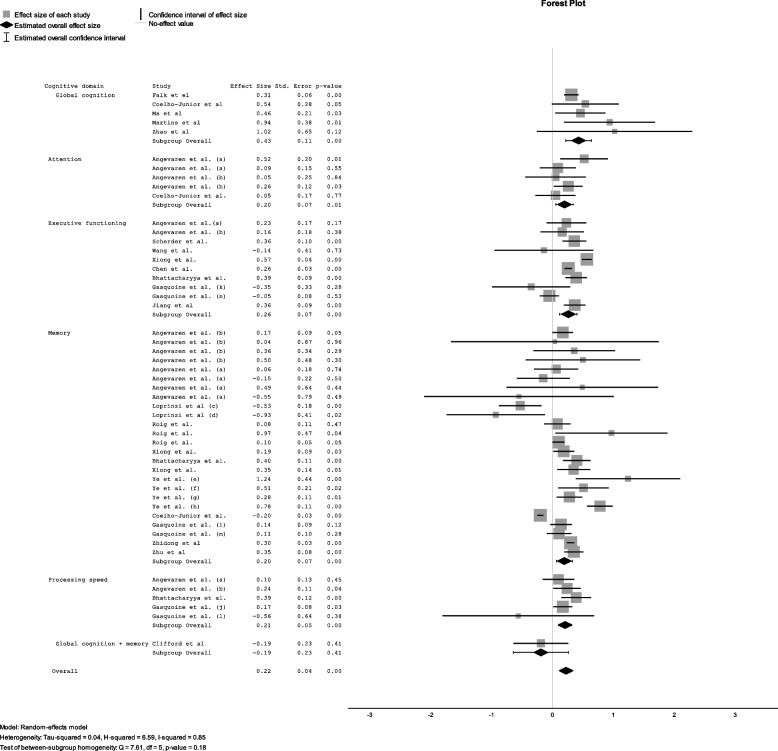
Effect size for each cognitive domain. Forest Plot showing the effect of exercise on cognitive domains (a = control group received no intervention, b = control group received any other intervention, c = exercise immediately before memory test, d = exercise during memory test, e = general memory, f = short-term memory, g = working memory, h = long-term memory, i = Digital span backwards, j = digit symbol test, k = trail making test a, l = trail making test b, m = letter fluency test, *n* = stroop test)

**Fig. 4:**
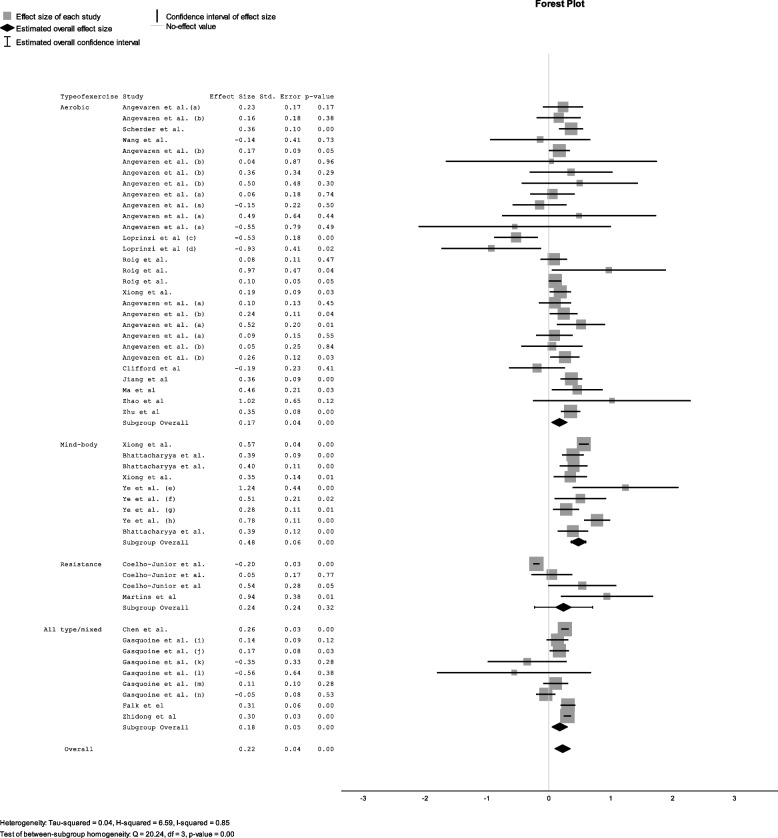
Effect size for each type of exercise. Forest Plot showing the effect of exercise on cognitive function. Sub-analyses are presented for different types of exercise (a = control group received no intervention, b = control group received any other intervention, c = exercise immediately before memory test, d = exercise during memory test, e = general memory, f = short-term memory, g = working memory, h = long-term memory, i = Digital span backwards, j = digit symbol test, k = trail making test a, l = trail making test b, m = letter fluency test, *n* = stroop test)

**Fig. 5:**
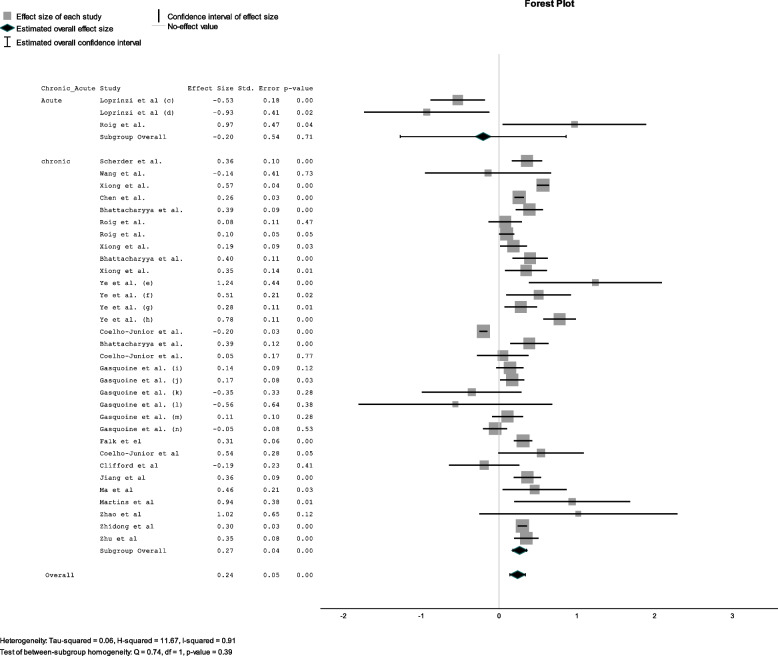
Effect size for each acute versus chronic exercise. Forest Plot showing the effect of acute and chronic exercise on cognitive function. Sub-analyses are presented for different types of exercise (a = control group received no intervention, b = control group received any other intervention, c = exercise immediately before memory test, d = exercise during memory test, e = general memory, f = short-term memory, g = working memory, h = long-term memory, i = Digital span backwards, j = digit symbol test, k = trail making test a, l = trail making test b, m = letter fluency test, n = stroop test)

Effect size data used in our analysis are presented in Fig. 3. Pooled results of all studies assessing the effect of exercise on cognition resulted in a small, positive effect in favour of exercise (d = 0.22; SE = 0.04; *p* < 0.01). Sub-analyses for each cognitive domain are presented in Fig. 3 (Global Cognition, Executive functioning, Memory, Attention and Processing speed), for type of exercise in Fig. 4 (aerobic, resistance and mind–body) and for duration of intervention (Acute vs Chronic) in Fig. 5.

In several studies included in this review, authors presented results separately for categories within a specific cognitive domain or separated their analysis based on study design (see Table 2). For example, Angevaren et al. presented effect sizes which were categorised into four types of memory (verbal memory, visual memory, working memory and memory function) as well as separating their analysis into 1/controls with no interventions and 2/controls with any other type of intervention [7]. Given that there is no overlap in the data included in each of these analyses we have chosen to include all relevant results (Table 2).

**Table 2:** Meta-data extracted from reviews

Author, year, reference	Type of exercise	Cognitive domain	Chronic/acute exercise	Effect size Cohens d or Hedges g^#^	lower limit	upper limit	SE
Angevaren et al., 2008 [7]	Aerobic (control group no exercise)	Attention (Auditory)	unclear	0.52	0.13	0.91	0.20
Angevaren et al., 2008 [7]	Aerobic (control group no exercise)	Attention (Visual)	unclear	0.09	-0.2	0.39	0.15
Angevaren et al., 2008 [7]	Aerobic (control group no exercise)	Executive functioning	unclear	0.23	-0.09	0.56	0.17
Angevaren et al., 2008 [7]	Aerobic (control group no exercise)	Memory (Verbal)	unclear	0.06	-0.3	0.42	0.18
Angevaren et al., 2008 [7]	Aerobic (control group no exercise)	Memory (Visual)	unclear	-0.15	-0.58	0.29	0.22
Angevaren et al., 2008 [7]	Aerobic (control group no exercise)	Memory (Working)	unclear	0.49	-0.76	1.73	0.64
Angevaren et al., 2008 [7]	Aerobic (control group no exercise)	Memory (memory functions)	unclear	-0.55	-2.11	1	0.79
Angevaren et al., 2008 [7]	Aerobic (control group no exercise)	Processing speed	unclear	0.1	-0.16	0.36	0.13
Angevaren et al., 2008 [7]	Aerobic (control group any other intervention)	Attention (Auditory)	unclear	0.05	-0.45	0.54	0.25
Angevaren et al., 2008 [7]	Aerobic (control group any other intervention)	Attention (Visual)	unclear	0.26	0.02	0.49	0.12
Angevaren et al., 2008 [7]	Aerobic (control group any other intervention)	Executive functioning	unclear	0.16	-0.2	0.51	0.18
Angevaren et al., 2008 [7]	Aerobic (control group any other intervention)	Memory (Verbal)	unclear	0.17	0.1	0.44	0.09
Angevaren et al., 2008 [7]	Aerobic (control group any other intervention)	Memory (Visual)	unclear	0.04	-1.66	1.75	0.87
Angevaren et al., 2008 [7]	Aerobic (control group any other intervention)	Memory (Working)	unclear	0.36	-0.31	1.03	0.34
Angevaren et al., 2008 [7]	Aerobic (control group any other intervention)	Memory (Memory functions)	unclear	0.5	-0.44	1.44	0.48
Angevaren et al., 2008 [7]	Aerobic (control group any other intervention)	Processing speed	unclear	0.24	0.01	0.46	0.11
Bhattacharyya et al., 2021 [9]	Mind–body	Memory	Chronic	0.4	0.17	0.62	0.11
Bhattacharyya et al., 2021 [9]	Mind–body	Processing speed	Chronic	0.39	0.15	0.64	0.13
Bhattacharyya et al., 2021 [9]	Mind–body	Executive functioning	Chronic	0.39	0.21	0.56	0.09
Chen et al., 2020 [10]	All types (aerobic. mind–body. resistance)	Executive functioning	Chronic	0.26^#^	0.2	0.32	0.03
Clifford et al., 2022 [45]	Aerobic	Global cognition and Memory	Chronic	-,190	-,650	-,270	0,23
Coelho-Junior et al., 2022 [8]	Resistance	Global cognition	Acute	,540	,000	1,080	0,28
Coelho-Junior et al., 2022 [8]	Resistance	Attention	Chronic	0.05	-0.28	0.38	0.17
Coelho-Junior et al., 2022 [8]	Resistance	Memory (short-term memory)	Chronic	-0.2	-0.25	-0.15	0.03
Falk et al., 2019 [40]	All types (aerobic. mind–body. resistance)	Global cognition	Chronic	,310^#^	,200	,430	0,06
Gasquoine and Chen, 2022 [41]	All types (aerobic. resistance. combination)	Memory	Chronic	0.14^#^	-0.03	0.32	0.09
Gasquoine and Chen, 2022 [41]	All types (aerobic. resistance. combination)	Processing speed	Chronic	0.17^#^	0.01	0.32	0.08
Gasquoine and Chen, 2022 [41]	All types (aerobic. resistance. combination)	Executive functioning	Chronic	-0.35^#^	-0.99	0.29	0.33
Gasquoine and Chen, 2022 [41]	All types (aerobic. resistance. combination)	Processing speed	Chronic	-0.56^#^	-1.8	0.69	0.64
Gasquoine and Chen, 2022 [41]	All types (aerobic. resistance. combination)	Memory	Chronic	0.11^#^	-0.09	0.31	0.10
Gasquoine and Chen, 2022 [41]	All types (aerobic. resistance. combination)	Executive functioning	Chronic	-0.05^#^	-0.21	0.1	0.08
Jiang et al., 2022 [47]	Aerobic	Executive functioning	Chronic	,365	,179	,550	0,09
Loprinzi et al., 2019 [48]	Aerobic (exercise immediatly prior to memory test)	Memory	Acute	-0.53	-0.88	-0.18	0.18
Loprinzi et al., 2019 [48]	Aerobic (exercise during memory test)	Memory	Acute	-0.93	-1.76	-0.15	0.41
Ma et al., 2023 [13]	Aerobic	Global cognition	Chronic	,460	,040	,880	0,21
Martins et al., 2022 [49]	Resistance	Global cognition	Chronic	,940	,200	1,680	0,38
Roig et al., 2013 [14]	Aerobic (long-term exercise)	Memory (short-term memory)	Chronic	0.1	-0.03	0.23	0.07
Roig et al., 2013 [14]	Aerobic (longterm memory)	Memory (long-term)	Chronic	,080	-,140	,310	0,11
Roig et al., 2013 [14]	Aerobic (longterm memory))	Memory (long term)	Acute	,970	,040	1,890	0,47
Scherder et al., 2014 [50]	Aerobic	Executive functioning	Chronic	0.36	0.16	0.55	0.10
Wang et al., 2021 [51]	Aerobic	Executive functioning	Chronic	-0.14	-0.95	0.67	0.41
Xiong et al., 2021 [39]	Aerobic	Memory	Chronic	0.186^#^	0.014	0.358	0.09
Xiong et al., 2021 [39]	Mind–body	Memory	Chronic	0.348^#^	0.079	0.617	0.14
Ye et al., 2021 [32]	Mind–body	Memory (General memory)	Chronic	1.24	0.38	2.09	0.44
Ye et al., 2021 [32]	Mind–body	Memory (short-term memory)	Chronic	0.51	0.1	0.93	0.21
Ye et al., 2021 [32]	Mind–body	Memory (Working memory)	Chronic	0.28	0.07	0.49	0.11
Ye et al., 2021 [32]	Mind–body	Memory (Long-term memory)	Chronic	0.78	0.57	0.99	0.11
Zhao et al., 2022 [52]	Aerobic	Global cognition	Chronic	1,020	-,250	2,300	0,65
Zhidong et al., 2021 [53]	All types	Memory	Chronic	,300	,230	,360	0,03
Zhu et al., 2023 [54]	Aerobic	Memory	Chronic	,351	,207	,514	0,08

Effect size data used in our meta-analysis

#### Sub-analyses for global cognition and specific cognitive domains

Global cognition was investigated in 5 studies and pooled data resulted in a moderate positive effect of exercise on cognition (d = 0.43; SE = 0,11; *p* < 0,001) [8, 13, 40, 49, 52].

Data presenting the effect of exercise on executive function was able to be extracted from 8 systematic reviews. Pooled data indicated a small, significant effect in favour of exercise (d = 0.26; SE = 0.07; *p* < 0.001).

Memory was the most frequently investigated cognitive domain and was reported in a total of 15 reviews, ten reporting effect size data relevant for this analysis. When studies reported separate results which were categorised by a specific type of memory (e.g. long-term and short-term memory) we included all results. Exercise was found to have a small, significant effect on pooled memory data (d = 0.20; SE = 0.05; *p* < 0.001).

Only two reviews were found to investigate the effect of exercise on attention. Angevaren et al. presented pooled data for auditory attention and visual attention as separate analyses [7]. Exercise was found to have a positive, but small effect on attention (d = 0.20; SE = 0.11; *p* = 0.01).

Four reviews investigated the effect of exercise on processing speed with three of these reporting relevant effect size data. Exercise was found to have a positive but small, effect on processing speed (d = 0.21; SE = 0.05; *p* < 0.001).

#### Sub analyses for types of exercise

Mind–body exercise had the greatest effect on cognition with a pooled effect size of d = 0.48 (SE = 0.06; *p* < 0.001) (Fig. 4). Five systematic reviews with meta-analyses included all together 31 original primary studies (overlaps excluded) that evaluated the effect of mind body exercise on cognitive function [9, 10, 32, 39, 53]. Eleven reviews investigated the effects of aerobic exercise on cognitive function with several studies evaluating the effects of aerobic exercise on multiple cognitive domains [7, 14, 48]. Aerobic exercise had a small effect on cognition (d = 0.17; SE = 0.04; *p* < 0.001), as did resistance exercise (d = 0.24; SE = 0,24; *p* < 0.32). The effect of mixed exercise on cognition was also small (d = 0.18; SE = 0.05; *p* < 0.001). Note that all cognitive domains were in this sub-analysis.

In order to investigate if the type of exercise had an effect of different cognitive domains we performed a separate analysis which stratified domains and exercise types. Results of this analysis can be found in Supplementary file S6. Mind–body exercise was not represented in every cognitive domain however was found to have the greatest effect size on executive function (d = 0.5; SE = 0.10; *p* < 0.001) and processing speed (d = 0,39; SE = 0.13; *p* < 0.01). Only aerobic and resistance exercise were investigated for their effects on global cognition and both resulted in moderate effect sizes (Aerobic d = 0.51; SE = 0.2; *p* = 0.01), Resistance d = 0.68; SE = 0.23; *p* < 0.01).

#### Sub analysis for acute versus chronic exercise

Nineteen reviews investigated the effects of chronic exercise on cognition while two studied the effects of acute exercise [14, 48]. Roig et al. [14] included analyses for both chronic and acute exercise. Chronic exercise had a small positive effect on cognition (d = 0,24;SE = 0,04;*p* < 0.001) while acute exercise has a small negative effect (d = -0.20; SE = 0.54; *p* = 0.71) (see Fig. 5).

#### Analysis of heterogeneity and publication bias

**Fig. 6:**
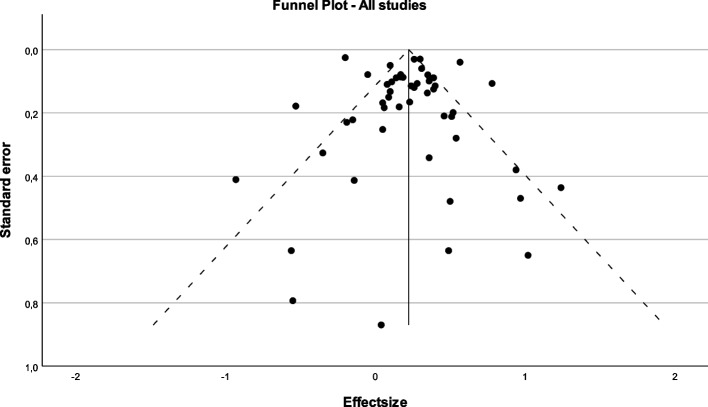
Funnel plot. Funnel plot including studies assessing the impact of exercise on cognitive functions. The plot shows the effect estimates from all studies and 95% confidence limits around the summary treatment effect

Variation across studies due to heterogeneity was very high (I^2^ = 85%). A funnel plot showing effect estimates from all studies and 95% confidence limits around the summary treatment effect is presented as Fig. 6. Egger’s test including all data revealed a significant deviation from zero (β_0_ = 0.23; CI = 0.107–0.350; t = 3.783; *p* < 0.001) confirming that small study effects may have influenced the results. This was further analysed by evaluating sub-groups (see Supplementary data S5). Results suggest that the heterogeneity is mainly due to the subgroups for memory and executive functions as well as the subgroups for aerobic and mixed exercise.

#### Sensitivity analysis

Supplementary Table S7 presents results of a sensitivity analysis showing the overall effect size for all reviews and the effect size calculated while systematically excluding A/ one review at a time and B/ reviews that included acute exercise interventions. Individual reviews which had the greatest influence on effect size were Ye et al. and Gasquoin et al. [32, 41]. The overall effect size varied from a minimum of 0.19 with Ye al al removed to a maximum of 0.25 with Gasquoine et al. removed. Removing any one study did not vary how the overall effect size would be interpreted, ie. a weak positive effect size [44]. Removing reviews including acute exercise interventions (*n* = 2) had little effect on the overall effect size which raised from d = 0.22 to d = 0.24.

## Discussion

To the best of our knowledge, this is the first umbrella review investigating the effects of exercise on cognitive functions in healthy adults (≥ 55 years of age). Our analyses indicate that aerobic and resistance exercise have a rather small effect on cognitive functioning while mind–body exercise has a moderate positive effect which would be more likely to result in a noticeable change in cognitive functions in adults over the age of 55. Chronic exercise was found to have a greater effect than acute exercise suggesting that regular training over a longer period is more beneficial for promoting cognitive functioning than a single bout of acute exercise.

Of the exercise modalities studied in this review, mind body exercise showed the greatest potential for slowing age-related cognitive decline. In contrast to aerobic and resistance exercise, which focus on cardiovascular fitness and strength, mind–body exercise combines movement sequences together with breathing control and attention regulation. This combination of physical and neurological resources may provide an explanation for the observed differences in the exercise modalities investigated. The potential relationship between physical activity and changes in neurological activity is supported by results from a recent systematic review which demonstrated that mind–body exercise induces changes in neural activity and functional connectivity in the brain [47], including the pre-frontal cortex which has an important role for cognitive functions [56, 57].

It is important to reflect on results related to exercise modality from a holistic perspective and with consideration of previous work demonstrating that aerobic and resistance exercise play an important role in maintaining physical function and in protecting against falls in older adults [58, 59]. Considering this previous work, combined with result of the present study, we suggest that a regular exercise routine including all three modalities (aerobic, resistance and mind–body) is most beneficial for promoting healthy aging.

Effect sizes across specific cognitive domains, executive functioning, memory, attention, and processing speed, ranged between 0.20 and 0.26 suggesting a relatively small effect when types of exercise are pooled. Whether these effects translate into clinically meaningful outcomes for older adults remains unclear. A sub-analysis for each domain, stratified by exercise type does indicate that different types of exercise may affect cognitive domains to different extents. For example, mind–body exercise had the greatest effect on executive function and processing speed, but no reviews reported the effects of mind–body exercise on attention or global cognition. These results are support by Ye et al. who reported mind–body exercise having a large effect on memory functions but only small to moderate effects on executive function [32]. Ren et al. call for additional research to clarify the effects of exercise types on different cognitive domains [60].

Effects of exercise on global cognition were higher than more specific cognitive domains (d = 0.43). Tests for global cognition aim to assess an individual’s general mental status and typically comprise of items representing a wide variety of different cognitive domains. For example, the Mini-Mental State Examination, included in many reviews, comprises of items that test memory, attention, speech perception and visuo-spatial skills [61]. Based on our study results it is not possible to determine why exercise has a greater effect on global cognition, although it is possible that the generalised global cognition tests included items covering cognitive domains that were not addressed in this review.

### Exercise intensity and duration

Exercise intensity was poorly reported in many of the reviews and may have affected results of this study. Exercise intensity has been suggested as an important factor in promoting healthy aging however, there appears to be significant discrepancy in the literature regarding the optimal intensity for promoting cognitive function [62–64].

Results of this umbrella review indicated that prolonged (chronic) exercise has a greater effect on cognitive function than a single (acute) bout of exercise. It should be noted however that only two reviews included data for acute exercise and these had contrasting results. Roig et al. concluded that acute aerobic exercise had a large, positive effect on memory functions by priming molecular processes involved in encoding and consolidation, while long-term exercise had negligible effects [14]. Loprinzi et al. found that acute aerobic exercise before memory encoding and during early consolidation had a negative effect on episodic memory [48]. Empirical studies involving younger adults have demonstrated an intensity-dependent effect of acute exercise on cognitive functions [65, 66]. El-Sayes et al. [67] propose a model of neuroplasticity which is induced by acute exercise and facilitates cognitive and motor function. They report that concentrations of BDNF and vascular endothelian growth factor (VEGF) increase after a bout of acute exercise and that this, together with increases in neurotransmitter and metabolite concentrations induces neuroplasticy within the brain to facilitate cognitive functions. It is important to recognise that this model based on studies involving adults in their early to late 20 s and further research is necessary to determine its validity with an older population.

Timing of the application of cognitive tests post exercise may be an important factor that influences results of empirical studies. In a recent systematic review, again involving young adults, a single, acute exercise workout immediately before a learning activity improved learning and memory functions and the effects remained for 30 to 120 min [68]. Unfortunately, most studies in our review did not report the time elapsing between physical activity and cognitive testing. This, along with clear details of exercise dosage (frequency, duration and intensity) are recommended as standard reporting parameters when studying exercise interventions.

An additional factor that must be taken into consideration when interpreting results of this umbrella review is the activity level of control groups. Some reviews only included studies with control group participants who did not undertake training [14], while others also included controls who undertook another form of exercise which would likely result in smaller effect sizes when comparing the means of intervention and control groups [7, 9, 51].

### Population

Many studies of exercise in older adults include both healthy individuals and those with mild cognitive decline. In this umbrella review we made a conscious decision to only include healthy individuals as previous work has identified differences in the effects of exercise on cognition between the two groups [10]. We also set the minimum age limit to 55 years. It has been found that cognitive outcomes are moderated by age with significant benefits for young-old (55–65 years) compared to older adults [10]. This decision was a rather pragmatic one based upon classifications used in previous studies and we recognised that results may have varied if we had limited our review to adults within a higher age range. Six of the selected studies in our meta-analysis included adults from the age of 55 and older [7, 9, 10, 46, 50, 51].

### Limitations

As is the case with all review studies, umbrella reviews are limited by the number, quality and comprehensiveness of data which is possible to extract from primary sources [69]. Inconsistency in use and classification of outcome measures representing specific cognitive domains as well as specific exercise interventions may limit the specificity of results in this review. Including sample populations from 55 years of age may also be considered a limitation of this study although age-related cognitive decline had been demonstrated to begin well before the age of 60 [6].

We are confident that a thorough search of the literature was performed in this umbrella review however with so many studies identified in the initial search it is possible that some relevant meta-analyses were overlooked. Our umbrella review also recorded high levels of heterogeneity suggesting high levels of variability in the data. This may be due to differences in target populations, measurement instruments or analytical methods. There were also a large number and variety of outcome measures that were included in reviews and inconsistencies in the cognitive domain classifications allocated to some measures. The variety of outcome measures together with overlap in the classification of outcomes is also likely to have contributed to high levels of heterogeneity. To manage heterogeneity we used a random-effects model for calculating effect size.

## Conclusions

This umbrella review has been a search for answers regarding the effects of exercise on cognitive functioning in healthy people aged 55 years and older. Results indicate that aerobic and resistance exercise have a rather small, and likely negligible effect, on cognitive functions in adults aged 55 years or older. A noteworthy finding is that mind body exercise had a moderate effect on cognition. Choice of cognitive outcomes along with timing and dosage of exercise may be key factors that influence the cognitive functions and require further investigation. Based upon results of this study we recommend that mind–body exercise be incorporated in the regular exercise routine of people aged 55 years and older. To promote healthy aging, mind–body exercise should serve as a complement to other types of exercise such as endurance training, resistance and balance activities all of which have been shown to improve body functions. It is anticipated that results of this review will be beneficial in supporting future studies, standardisation of study designs and the development of guidelines including mind body exercises for interventions which support healthy aging.

### Supplementary Information

**Additional file 1: Supplementary S1. **Search strategies.

**Additional file 2:** **Supplement S2. **Characteristics of excluded studies.

**Additional file 3:** **Supplement S3.** Included studies overlap.

**Additional file 4:** **Supplement 4.** Cognitive domains and tests.

**Additional file 5:** **Supplement S5.** Funnel plots for subgroups.

**Additional file 6: Supplement S6.** Type of exercise stratified by cognitive domain.

**Additional file 7: Supplement S7.** A) Sensitivity analysis showing effect size when each study is individually removed from the analysis.

## Data Availability

The datasets used and/or analysed during the current study are available from the corresponding author on reasonable request.

## Abbreviations

BDNF: Brain-derived neutrophic factor
CI: Confidence interval
CCA: Corrected covered area method
DOI´s: Digital Object Identifies
GLDP1: Glycosylphosphatidylinositol-Specific Phospholipase S1
IL-6: Interleukin-6
MA: Meta-analyses
MCI: Mild cognitive impairment
MD: Missing data
NRCTs: Non-randomized controlled trials
PMIDs: PrubMedIDs
RCTs: Randomized Controlled Trials
SMD: Standardized mean difference
