# Supplementary material for: Mind body exercise improves cognitive function more than aerobic- and resistance exercise in healthy adults aged 55 years and older – an umbrella review
Source: Eur Rev Aging Phys Act. 2023 Aug 9;20:15. doi: 10.1186/s11556-023-00325-4 (PMC10413530; doi:10.1186/s11556-023-00325-4)
Supplement: Supplementary file 1 — Additional file 1: Supplementary S1. Search strategies. [file 11556_2023_325_MOESM1_ESM.pdf]

Supplementary S1. Search strategies

CINAHL with Full Text (EBSCOhost) n = 710 1
Cochrane Library (Wiley) n = 500 6
MEDLINE (EBSCOhost) n = 871 8
PsycInfo (ProQuest) n = 1249 14
Scopus (Elsevier) n = 3382 16
Web of Science Core Collection n = 2515 17

CINAHL with Full Text (EBSCOhost) n = 710

Date of final search: 30 May 2023
Years of coverage: 1976 – 2023

#	Query	Results
	**Age 55+**	
S1	(MH "Aged") OR (MH "Frail Elderly") OR (MH "Aged, 80 and over") OR (MH "Middle Age") OR (MH "Geriatrics")	1,441,443
S2	TI (aged OR ageing OR aging OR elder* OR (old* N3 (adult* OR person* OR people)) OR senior* OR centenarian* OR nonagenarian* OR octogenarian* OR “oldest old” OR “old age” OR “middle age” OR geriatric* OR gerontolog*) OR AB (aged OR ageing OR aging OR elder* OR (old* N3 (adult* OR person* OR people)) OR senior* OR centenarian* OR nonagenarian* OR octogenarian* OR “oldest old” OR “old age” OR “middle age” OR geriatric* OR gerontolog*)	504.882
S3	S1 OR S2	1,668,192
	**Exercise, physical activity, sports**	
S4	(MH "Exercise+") OR (MH "Physical Fitness+") OR (MH "Physical Activity")	180,208
S5	TI ((aerobic OR acute OR isometric OR physical) N3 (activit* OR conditioning OR exercise* OR fitness OR training*)) OR AB ((aerobic OR acute OR isometric OR physical) N3 (activit* OR conditioning OR exercise* OR fitness OR training*))	104,405
S6	TI ((cool-down OR cooldown OR (cool N3 down) OR cooling-down OR recovery OR warm-down OR (warm N3 down) OR warming-down OR warm-up OR warmup OR warming-up OR (warm* N3 up)) N3 exercise*) OR AB ((cool-down OR cooldown OR (cool N3 down) OR cooling-down OR recovery OR warm-down OR (warm N3 down) OR warming-down OR warm-up OR warmup OR warming-up OR (warm* N3 up)) N3 exercise*)	2,580

S7	TI (gymnastics OR calisthenics) OR AB (gymnastics OR calisthenics)	1,121
S8	TI ((muscle* OR active OR ballistic OR dynamic OR isometric OR passive OR PNF OR “Proprioceptive Neuromuscular Facilitation*” OR relaxed OR static OR static-active OR (static N3 active) OR static-passive OR (static N3 passive)) N3 stretching) OR AB ((muscle* OR active OR ballistic OR dynamic OR isometric OR passive OR PNF OR “Proprioceptive Neuromuscular Facilitation*” OR relaxed OR static OR static-active OR (static N3 active) OR static-passive OR (static N3 passive)) N3 stretching)	1,643
S9	TI (human N3 (conditioning OR training) N3 physical) OR AB (human N3 (conditioning OR training) N3 physical)	12
S10	TI ((circuit OR circuit-based) N3 (exercise* OR training*)) OR AB ((circuit OR circuit-based) N3 (exercise* OR training*))	479
S11	TI (endurance N3 (exercise* OR training*)) OR AB (endurance N3 (exercise* OR training*))	4,341
S12	TI ((high-intensity OR (high N3 intensity) OR interval OR intermittent OR sprint) N3 (exercise* OR interval* OR training*)) OR AB ((high-intensity OR (high N3 intensity) OR interval OR intermittent OR sprint) N3 (exercise* OR interval* OR training*))	8,028
S13	TI ((plyometric OR cycle OR stretch-shortening OR (stretch N3 shortening)) N3 (drill* OR exercise* OR training*)) OR AB ((plyometric OR cycle OR stretch-shortening OR (stretch N3 shortening)) N3 (drill* OR exercise* OR training*))	2,135
S14	TI ((resistance OR strength OR strengthening OR weight-bearing OR weight-lifting OR (weight* N3 (bear* OR lift*))) N3 (activit* OR conditioning OR exercise* OR training* OR program*)) OR AB ((resistance OR strength OR strengthening OR weight-bearing OR weight-lifting OR (weight* N3 (bear* OR lift*))) N3 (activit* OR conditioning OR exercise* OR training* OR program*))	23,095
S15	TI (run OR running OR runnings OR jog OR jogging OR joggings OR marathon* OR ultramarathon*) OR AB (run OR running OR runnings OR jog OR jogging OR joggings OR marathon* OR ultramarathon*)	42,147
S16	TI (swim* OR swimming OR walk* OR walking OR (stair* N3 (climbing OR navigation))) OR AB (swim* OR swimming OR walk* OR walking OR (stair* N3 (climbing OR navigation)))	62,733
S17	TI (cardiorespiratory N3 fitness) OR AB (cardiorespiratory N3 fitness)	3,604
S18	TI (physical N3 (endurance OR fitness OR stamina)) OR AB (physical N3 (endurance OR fitness OR stamina))	7,383

S19	TI ((physical OR functional) N3 performance*) OR AB ((physical OR functional) N3 performance*)	12,952
S20	(MH "Sports+") OR (MH "Tai Chi")	90,725
S21	TI (sport* OR athletic*) OR AB (sport* OR athletic*) OR TI (baseball* OR softball* OR basketball* OR netball* OR bike* OR bicycl* OR boxing* OR cricket) OR AB (baseball* OR softball* OR basketball* OR netball* OR bike* OR bicycl* OR boxing* OR cricket) OR TI (football* OR rugby OR rugbies OR golf* OR hockey* OR mountaineering*) OR AB (football* OR rugby OR rugbies OR golf* OR hockey* OR mountaineering*) OR TI (Aikido OR “Hap Ki Do” OR Judo OR Karate OR Jujitsu OR (martial N3 arts*) OR “Kung Fu” OR (gong N3 fu) OR Gongfu OR “Tae Kwon Do” OR Wushu OR Tai-Ji OR (Tai N3 Chi) OR (Tai N3 Ji N3 Quan) OR Taiji OR Taijiquan OR “T’ai Chi”) OR AB (Aikido OR “Hap Ki Do” OR Judo OR Karate OR Jujitsu OR (martial N3 arts*) OR “Kung Fu” OR (gong N3 fu) OR Gongfu OR “Tae Kwon Do” OR Wushu OR Tai-Ji OR (Tai N3 Chi) OR (Tai N3 Ji N3 Quan) OR Taiji OR Taijiquan OR “T’ai Chi”)	87,008
S22	TI (badminton OR lacrosse OR racketball OR racquetball OR ((racquet OR racket OR squash) N3 ball)) OR AB (badminton OR lacrosse OR racketball OR racquetball OR ((racquet OR racket OR squash) N3 ball)) OR TI (tennis OR skating* OR skateboarding* OR soccer*) OR AB (tennis OR skating* OR skateboarding* OR soccer*) OR TI ((return OR resumption*) N3 (play OR recreational)) OR AB ((return OR resumption*) N3 (play OR recreational)) OR TI (snowmobiling OR sledding OR snowboarding OR skiing*) OR AB (snowmobiling OR sledding OR snowboarding OR skiing*)	13,349
S23	TI (parathletic* OR para-athletic* OR para-sport* OR (athletic* N3 (adaptive OR disabled OR para OR wheelchair*))) OR AB (parathletic* OR para-athletic* OR para-sport* OR (athletic* N3 (adaptive OR disabled OR para OR wheelchair*))) OR TI (track* OR “Field and Track”) OR AB (track* OR “Field and Track”) OR TI (volleyball*) OR AB (volleyball*)	47,954
S24	TI (boating OR canoeing OR diving* OR kayaking OR rowing OR surfing OR surfboarding OR (water N3 polo) OR (water N3 skiing)) OR AB (boating OR canoeing OR diving* OR kayaking OR rowing OR surfing OR surfboarding OR (water N3 polo) OR (water N3 skiing)) OR TI ((weight N3 lifting*) OR wrestling*) OR AB ((weight N3 lifting*) OR wrestling*)	4,260
S25	(MH "Therapeutic Exercise+")	61,929
S26	TI ((exercise N3 movement N3 (technics OR technique*)) OR pilates OR pilates-based) OR AB ((exercise N3 movement N3 (technics OR technique*)) OR pilates OR pilates-based) OR TI ((breathing N3 exercise*) OR (respiratory N3 muscle N3 training*)) OR AB ((breathing N3 exercise*) OR (respiratory N3 muscle N3 training*))	2,730
S27	(MH "Qigong") OR (MH "Yoga")	9,248

S28	TI (Qigong OR “Qi Gong” OR “Ch’i Kung” OR yoga) OR AB (Qigong OR “Qi Gong” OR “Ch’i Kung” OR yoga)	8,763
S29	(MH "Dancing+") OR (MH "Dance Therapy")	4,968
S30	TI (dance OR dancing OR ballet) OR AB (dance OR dancing OR ballet)	5,584
S31	S4 OR S5 OR S6 OR S7 OR S8 OR S9 OR S10 OR S11 OR S12 OR S13 OR S14 OR S15 OR S16 OR S17 OR S18 OR S19 OR S20 OR S21 OR S22 OR S23 OR S24 OR S25 OR S26 OR S27 OR S28 OR S29 OR S30	466,144
	**Cognitive function**	
S32	(MH "Mental Processes") OR (MH "Executive Function")	13,517
S33	TI ((mental N3 process*) OR (cognitive N3 process*) OR (information N3 processing)) OR AB ((mental N3 process*) OR (cognitive N3 process*) OR (information N3 processing)) OR TI (executive N3 (control* OR function*)) OR AB (executive N3 (control* OR function*))	26,843
S34	(MH "Cognition") OR (MH "Consciousness") OR (MH "Imagination") OR (MH "Intuition") OR (MH "Intention")	83,330
S35	TI (cognition* OR (cognitive N3 function*) OR awareness*) OR AB (cognition* OR (cognitive N3 function*) OR awareness*) OR TI (comprehension OR understanding* OR consciousness* OR imagination* OR intuition* OR intention*) OR AB (comprehension OR understanding* OR consciousness* OR imagination* OR intuition* OR intention*)	444,416
S36	(MH "Learning") OR (MH "Problem-Based Learning") OR (MH "Self Directed Learning")	32,451
S37	TI (learning* OR phenomenography OR (memory N3 training)) OR AB (learning* OR phenomenography OR (memory N3 training))	153,806
S38	(MH "Conditioning (Psychology)")	2,036
S39	TI ((psycholog* N3 conditioning) OR (social N3 learning N3 theor*)) OR AB ((psycholog* N3 conditioning) OR (social N3 learning N3 theor*))	737
S40	(MH "Memory") OR (MH "Associative Memory") OR (MH "Episodic Memory") OR (MH "Memory, Short Term") OR (MH "Recognition (Psychology)") OR (MH "Semantic Memory") OR (MH "Skill Retention")	35,687
S41	TI (memory OR memories OR ((immediate OR mental) N3 recall*) OR (psycholog* N3 (recognition OR retention)) OR familiarity OR (repetition N3 priming)) OR AB (memory OR memories OR ((immediate OR mental) N3 recall*) OR (psycholog* N3 (recognition OR retention)) OR familiarity OR (repetition N3 priming)) OR TI ((skill* OR training) N3 (carryover OR	67.633

	retain* OR retention*)) OR AB((skill* OR training) N3 (carryover OR retain* OR retention*))	
S42	(MH "Transfer (Psychology)")	545
S43	TI ((psychology OR learning OR training) N3 transfer*) OR AB ((psychology OR learning OR training) N3 transfer*)	2,031
S44	(MH "Mentalization") OR (MH "Thinking") OR (MH "Critical Thinking")	11,776
S45	TI (mentalization OR mentalizing) OR AB (mentalization OR mentalizing) OR TI (thinking OR thought*) OR AB (thinking OR thought*)	86,824
S46	(MH "Concept Formation") OR (MH "Concept Mapping")	3,430
S47	TI (conceptualization OR conceptualisation OR (concept* N3 (formation OR learning OR mapping))) OR AB (conceptualization OR conceptualisation OR (concept* N3 (formation OR learning OR mapping)))	12,173
S48	(MH "Creativeness")	6,424
S49	TI (creativity OR creativeness OR (creative N3 (ability OR abilities OR thinking)) OR innovativeness OR originality) OR AB (creativity OR creativeness OR (creative N3 (ability OR abilities OR thinking)) OR innovativeness OR originality)	11,354
S50	(MH "Decision Making") OR (MH "Judgment")	63,514
S51	TI (decision* N3 making) OR AB (decision* N3 making) OR TI ((approach* OR choice*) N3 (behavior* OR behaviour*)) OR AB ((approach* OR choice*) N3 (behavior* OR behaviour*)) OR TI (judgment* OR heuristic*) OR AB (judgment* OR heuristic*)	106,265
S52	(MH "Problem Identification") OR (MH "Problem Solving") OR (MH "Brainstorming")	13,352
S53	TI (brainstorming OR problem* N3 (identify OR identification OR solve* OR solving)) OR AB (brainstorming OR problem* N3 (identify OR identification OR solve* OR solving))	22,694
S54	TI (psychological N3 (anticipation OR expectation*)) OR AB (psychological N3 (anticipation OR expectation*)) OR TI (psychological N3 anticipation) OR AB (psychological N3 anticipation) OR TI ((brain OR cognitive) N3 reserve*) OR AB ((brain OR cognitive) N3 reserve*) OR TI ((cognitive N3 (awareness* OR control* OR knowledge* OR monitoring*)) OR metaemotion* OR meta-emotion* OR metamemory OR meta-memory OR metamemories OR meta-memories OR (meta N3 (emotion* OR memory OR memories))) OR AB (cognitive N3 (awareness* OR control* OR knowledge* OR monitoring*)) OR metaemotion* OR meta-emotion* OR metamemory OR meta-memory OR metamemories OR meta-memories	8,110

	OR (meta N3 (emotion* OR memory OR memories))) OR TI (higher N3 nervous N3 activit*) OR AB (higher N3 nervous N3 activit*)	
S55	TI ((psycholog* OR response OR stimulus) N3 (generalization* OR generalisation*)) OR AB ((psycholog* OR response OR stimulus) N3 (generalization* OR generalisation*)) OR TI (overlearning*) OR AB (overlearning*) OR TI (psycholog* N3 (practic* OR set*)) OR AB (psycholog* N3 (practic* OR set*)) OR TI (spatial N3 (ability OR abilities OR navigation* OR visualization* OR visualization*)) OR AB (spatial N3 (ability OR abilities OR navigation* OR visualization* OR visualization*))	4,381
S56	S32 OR S33 OR S34 OR S35 OR S36 OR S37 OR S38 OR S39 OR S40 OR S41 OR S42 OR S43 OR S44 OR S45 OR S46 OR S47 OR S48 OR S49 OR S50 OR S51 OR S52 OR S53 OR S54 OR S55	896,649
S57	S3 AND S31 AND S56<br>Limiters - Peer Reviewed	21,603
	**Systematic review and/or meta-analysis**	
S58	TI (meta-analysis OR "meta analysis" OR "systematic review") OR AB (meta-analysis OR "meta analysis" OR "systematic review")	173,286
S59	S57 AND S58	659
S60	Limiters - Publication Type: Meta Analysis, Systematic Review	136,083
S61	S57 AND S60	710
S62	S59 OR S61	710
	Interface - EBSCOhost Research Databases<br>Search Screen - Advanced Search<br>Search modes - Boolean/Phrase	

Cochrane Library (Wiley) n = 500

Date of final search: 30 May 2023
Years of coverage: 1996 – 2023

ID	Search	Hits
#1	(aged OR ageing OR aging OR elder* OR (old* NEAR/3 (adult* OR person* OR people)) OR senior* OR centenarian* OR nonagenarian* OR octogenarian* OR “oldest old” OR “old age” OR “middle age” OR “middle adulthood” OR geriatric* OR gerontolog*):ti,ab,kw	639,548
#2	(((aerobic OR acute OR isometric OR physical) NEAR/3 (activit* OR conditioning OR exercise* OR fitness OR training*)) OR ((cool-down OR cooldown OR (cool NEAR/3 down) OR cooling-down OR recovery OR warm-down OR (warm NEAR/3 down) OR warming-down OR warm-up OR warmup OR warming-up OR (warm* NEAR/3 up)) NEAR/3 exercise*)	341,808

	OR gymnastics OR calisthenics OR ((muscle* OR active OR ballistic OR dynamic OR isometric OR passive OR PNF OR “Proprioceptive Neuromuscular Facilitation*” OR relaxed OR static OR static-active OR (static NEAR/3 active) OR static-passive OR (static NEAR/3 passive)) NEAR/3 stretching) OR (human NEAR/3 (conditioning OR training) NEAR/3 physical) OR ((circuit OR circuit-based) NEAR/3 (exercise* OR training*)) OR (endurance NEAR/3 (exercise* OR training*)) OR ((high-intensity OR (high NEAR/3 intensity) OR interval OR intermittent OR sprint) NEAR/3 (exercise* OR interval* OR training*)) OR ((plyometric OR cycle OR stretch-shortening OR (stretch NEAR/3 shortening)) NEAR/3 (drill* OR exercise* OR training*)) OR ((resistance OR strength OR strengthening OR weight-bearing OR weight-lifting OR (weight* NEAR/3 (bear* OR lift*))) NEAR/3 (activit* OR conditioning OR exercise* OR training* OR program*)) OR run OR running OR runnings OR jog OR jogging OR joggings OR marathon* OR ultramarathon* OR swim* OR swimming OR walk* OR walking OR (stair* NEAR/3 (climbing OR navigation)) OR sport* OR athletic* OR (cardiorespiratory NEAR/3 fitness) OR (physical NEAR/3 (endurance OR fitness OR stamina)) OR ((physical OR functional) NEAR/3 performance*) OR baseball* OR softball* OR basketball* OR netball* OR bike* OR bicycl* OR boxing* OR cricket OR football* OR rugby OR rugbies OR golf* OR hockey* OR mountaineering* OR Aikido OR “Hap Ki Do” OR Judo OR Karate OR Jujitsu OR (martial NEAR/3 arts*) OR “Kung Fu” OR (gong NEAR/3 fu) OR Gongfu OR “Tae Kwon Do” OR Wushu OR Tai-Ji OR (Tai NEAR/3 Chi) OR (Tai NEAR/3 Ji NEAR/3 Quan) OR Taiji OR Taijiquan OR “T’ai Chi” OR badminton OR lacrosse OR racketball OR racquetball OR ((racquet OR racket OR squash) NEAR/3 ball) OR tennis OR skating* OR skateboarding* OR soccer* OR ((return OR resumption*) NEAR/3 (play OR recreational)) OR snowmobiling OR sledding OR snowboarding OR skiing* OR parathletic* OR para-athletic* OR para-sport* OR track* OR “Field and Track” OR volleyball* OR boating OR canoeing OR diving* OR kayaking OR rowing OR surfing OR surfboarding OR (water NEAR/3 polo) OR (water NEAR/3 skiing) OR (weight NEAR/3 lifting*) OR wrestling* OR (exercise NEAR/3 movement NEAR/3 (technics OR technique*)) OR pilates OR pilates-based OR (breathing NEAR/3 exercise*) OR (respiratory NEAR/3 muscle NEAR/3 training*) OR Qigong OR “Qi Gong” OR “Ch’i Kung” OR yoga OR dance OR dancing OR ballet):ti,ab,kw	
#3	((mental NEAR/3 process*) OR (cognitive NEAR/3 process*) OR (information NEAR/3 processing) OR (psychological NEAR/3 (anticipation OR expectation*)) OR cognition* OR (cognitive NEAR/3 (ability OR abilities OR flexibility OR function*)) OR awareness* OR ((brain OR cognitive) NEAR/3 reserve*) OR comprehension OR understanding* OR consciousness* OR imagination* OR intention* OR intuition* OR (executive NEAR/3 (control* OR function*)) OR (higher NEAR/3 nervous NEAR/3 activit*) OR meta-cognition OR metacognition OR (meta NEAR/3 cognition) OR ((metacognitive OR meta-cognitive OR (meta NEAR/3 cognitive)) NEAR/3 (awareness* OR control* OR knowledge* OR monitoring*)) OR metaemotion* OR meta-emotion* OR metamemory OR meta-memory OR metamemories OR meta-memories OR (meta NEAR/3 (emotion* OR memory OR memories)) OR ((skill* OR training) NEAR/3 (carryover OR retain* OR retention*)) OR learning* OR phenomenography OR (psycholog* NEAR/3 conditioning) OR (social NEAR/3 learning NEAR/3 theor*) OR ((psycholog* OR response OR stimulus) NEAR/3	218,572

	(generalization* OR generalisation*)) OR memory OR memories OR ((immediate OR mental) NEAR/3 recall*) OR (psycholog* NEAR/3 (recognition OR retention)) OR familiarity OR (repetition NEAR/3 priming) OR overlearning* OR (spatial NEAR/3 (ability OR abilities OR navigation* OR visualization* OR visualization*)) OR ((psychology OR learning OR training) NEAR/3 transfer*) OR mentalization OR mentalizing OR thinking OR thought* OR conceptualization OR conceptualisation OR (concept* NEAR/3 (formation OR learning)) OR creativity OR creativeness OR (creative NEAR/3 (ability OR abilities OR thinking)) OR innovativeness OR originality OR (decision* NEAR/3 making) OR ((approach* OR choice*) NEAR/3 (behavior* OR behaviour*)) OR judgment* OR heuristic* OR brainstorming OR (problem* NEAR/3 (identify OR identification OR solve* OR solving))):ti,ab,kw	
#4	#1 AND #2 AND #3 in Cochrane Reviews	500

MEDLINE (EBSCOhost) n = 871

Date of final search: 30 May 2023
Years of coverage: 1809 – 2023

#	**Query**	**Results**
	**Age 55+**	
S1	(MH "Aged") OR (MH "Frail Elderly") OR (MH "Aged, 80 and over") OR (MH "Middle Age") OR (MH "Geriatrics")	3,466,687
S2	TI (aged OR ageing OR aging OR elder* OR (old* N3 (adult* OR person* OR people)) OR senior* OR centenarian* OR nonagenarian* OR octogenarian* OR “oldest old” OR “old age” OR “middle age” OR “middle adulthood” OR geriatric* OR gerontolog*) OR AB (aged OR ageing OR aging OR elder* OR (old* N3 (adult* OR person* OR people)) OR senior* OR centenarian* OR nonagenarian* OR octogenarian* OR “oldest old” OR “old age” OR “middle age” OR “middle adulthood” OR geriatric* OR gerontolog*)	1,343,995
S3	S1 OR S2	4,246,374
	**Exercise, physical activity, sports**	
S4	(MH "Exercise") OR (MH "Cool-Down Exercise") OR (MH "Warm-Up Exercise")	141,003
S5	TI ((aerobic OR acute OR isometric OR physical) N3 (activit* OR conditioning OR exercise* OR fitness OR training*)) OR AB ((aerobic OR acute OR isometric OR physical) N3 (activit* OR conditioning OR exercise* OR fitness OR training*)) OR TI ((cool-down OR cooldown OR (cool N3 down) OR cooling-down OR recovery OR warm-down OR (warm N3 down) OR warming-down OR warm-up OR warmup OR warming-up OR (warm* N3 up)) N3 exercise*) OR AB ((cool-down OR cooldown OR (cool N3 down) OR cooling-down OR recovery OR warm-down OR (warm	230,968

	N3 down) OR warming-down OR warm-up OR warmup OR warming-up OR (warm* N3 up)) N3 exercise*)	
S6	(MH "Gymnastics") OR (MH "Muscle Stretching Exercises")	4,379
S7	TI (gymnastics OR calisthenics) OR AB (gymnastics OR calisthenics) OR TI ((muscle* OR active OR ballistic OR dynamic OR isometric OR passive OR PNF OR “Proprioceptive Neuromuscular Facilitation*” OR relaxed OR static OR static-active OR (static N3 active) OR static-passive OR (static N3 passive)) N3 stretching) OR AB ((muscle* OR active OR ballistic OR dynamic OR isometric OR passive OR PNF OR “Proprioceptive Neuromuscular Facilitation*” OR relaxed OR static OR static-active OR (static N3 active) OR static-passive OR (static N3 passive)) N3 stretching)	6,291
S8	(MH "Physical Conditioning, Human") OR (MH "Circuit-Based Exercise") OR (MH "Endurance Training")	3,553
S9	TI (human N3 (conditioning OR training) N3 physical) OR AB (human N3 (conditioning OR training) N3 physical) OR TI ((circuit OR circuit-based) N3 (exercise* OR training*)) OR AB ((circuit OR circuit-based) N3 (exercise* OR training*)) OR TI (endurance N3 (exercise* OR training*)) OR AB (endurance N3 (exercise* OR training*))	13,873
S10	(MH "High-Intensity Interval Training") OR (MH "Plyometric Exercise")	2,908
S11	TI ((high-intensity OR (high N3 intensity) OR interval OR intermittent OR sprint) N3 (exercise* OR interval* OR training*)) OR AB ((high-intensity OR (high N3 intensity) OR interval OR intermittent OR sprint) N3 (exercise* OR interval* OR training*)) OR TI ((plyometric OR cycle OR stretch-shortening OR (stretch N3 shortening)) N3 (drill* OR exercise* OR training*)) OR AB ((plyometric OR cycle OR stretch-shortening OR (stretch N3 shortening)) N3 (drill* OR exercise* OR training*))	28,122
S12	(MH "Resistance Training")	12,185
S13	TI ((resistance OR strength OR strengthening OR weight-bearing OR weight-lifting OR (weight* N3 (bear* OR lift*))) N3 (activit* OR conditioning OR exercise* OR training* OR program*)) OR AB ((resistance OR strength OR strengthening OR weight-bearing OR weight-lifting OR (weight* N3 (bear* OR lift*))) N3 (activit* OR conditioning OR exercise* OR training* OR program*))	53,329
S14	(MH "Running") OR (MH "Jogging") OR (MH "Marathon Running")	23,669
S15	TI (run OR running OR runnings OR jog OR jogging OR joggings OR marathon* OR ultramarathon*) OR AB (run OR running OR runnings OR jog OR jogging OR joggings OR marathon* OR ultramarathon*)	190,629
S16	(MH "Swimming") OR (MH "Walking") OR (MH "Stair Climbing")	60,991

S17	TI (swim* OR swimming OR walk* OR walking OR (stair* N3 (climbing OR navigation))) OR AB (swim* OR swimming OR walk* OR walking OR (stair* N3 (climbing OR navigation)))	192,300
S18	(MH "Sports") OR (MH "Athletic Performance")	47,622
S19	TI (sport* OR athletic*) OR AB (sport* OR athletic*)	110,654
S20	(MH "Cardiorespiratory Fitness") OR (MH "Physical Endurance") OR (MH "Physical Fitness") OR (MH "Physical Functional Performance")	52,788
S21	TI (cardiorespiratory N3 fitness) OR AB (cardiorespiratory N3 fitness) OR TI (physical N3 (endurance OR fitness OR stamina)) OR AB (physical N3 (endurance OR fitness OR stamina)) OR TI ((physical OR functional) N3 performance*) OR AB ((physical OR functional) N3 performance*)	48,361
S22	(MH "Baseball") OR (MH "Basketball") OR (MH "Bicycling") OR (MH "Boxing") OR (MH "Cricket Sport") OR (MH "Football") OR (MH "Golf") OR (MH "Hockey") OR (MH "Mountaineering")	32,874
S23	TI (baseball* OR softball* OR basketball* OR netball* OR bike* OR bicycl* OR boxing* OR cricket) OR AB (baseball* OR softball* OR basketball* OR netball* OR bike* OR bicycl* OR boxing*) OR TI (football* OR rugby OR rugbies OR golf* OR hockey* OR mountaineering*) OR AB (football* OR rugby OR rugbies OR golf* OR hockey* OR mountaineering*)	62,699
S24	(MH "Martial Arts") OR (MH "Tai Ji")	3,247
S25	TI (Aikido OR “Hap Ki Do” OR Judo OR Karate OR Jujitsu OR (martial N3 arts*) OR “Kung Fu” OR (gong N3 fu) OR Gongfu OR “Tae Kwon Do” OR Wushu OR Tai-Ji OR (Tai N3 Chi) OR (Tai N3 Ji N3 Quan) OR Taiji OR Taijiquan OR “T’ai Chi”) OR AB (Aikido OR “Hap Ki Do” OR Judo OR Karate OR Jujitsu OR (martial N3 arts*) OR “Kung Fu” OR (gong N3 fu) OR Gongfu OR “Tae Kwon Do” OR Wushu OR Tai-Ji OR (Tai N3 Chi) OR (Tai N3 Ji N3 Quan) OR Taiji OR Taijiquan OR “T’ai Chi”)	4,774
S26	(MH "Racquet Sports") OR (MH "Tennis") OR (MH "Skating") OR (MH "Soccer")	13,600
S27	TI (badminton OR lacrosse OR racketball OR racquetball OR ((racquet OR racket OR squash) N3 ball)) OR AB (badminton OR lacrosse OR racketball OR racquetball OR ((racquet OR racket OR squash) N3 ball)) OR TI (tennis OR skating* OR skateboarding* OR soccer*) OR AB (tennis OR skating* OR skateboarding* OR soccer*)	17,701
S28	(MH "Return to Sport")	2, 485
S29	TI ((return OR resumption*) N3 (play OR recreational)) OR AB ((return OR resumption*) N3 (play OR recreational))	2,864

S30	(MH "Snow Sports") OR (MH "Skiing") OR (MH "Team Sports") OR (MH "Track and Field") OR (MH "Volleyball")	6,096
S31	TI (snowmobiling OR sledding OR snowboarding OR skiing* OR track* OR “Field and Track” OR volleyball*) OR AB (snowmobiling OR sledding OR snowboarding OR skiing* OR track* OR “Field and Track” OR volleyball*)	203,482
S32	(MH "Sports for Persons with Disabilities")	317
S33	TI (parathletic* OR para-athletic* OR para-sport* OR (athletic* N3 (adaptive OR disabled OR para OR wheelchair*))) OR AB (parathletic* OR para-athletic* OR para-sport* OR (athletic* N3 (adaptive OR disabled OR para OR wheelchair*)))	136
S34	(MH "Water Sports") OR (MH "Diving") OR (MH "Weight Lifting") OR (MH "Wrestling")	13,913
S35	TI (boating OR canoeing OR diving* OR kayaking OR rowing OR surfing OR surfboarding OR (water N3 polo) OR (water N3 skiing)) OR AB (boating OR canoeing OR diving* OR kayaking OR rowing OR surfing OR surfboarding OR (water N3 polo) OR (water N3 skiing)) OR TI ((weight N3 lifting*) OR wrestling*) OR AB ((weight N3 lifting*) OR wrestling*)	14,254
S36	(MH "Exercise Movement Techniques")	866
S37	TI ((exercise N3 movement N3 (technics OR technique*)) OR pilates OR pilates-based) OR AB ((exercise N3 movement N3 (technics OR technique*)) OR pilates OR pilates-based)	801
S38	(MH "Breathing Exercises") OR (MH "Qigong") OR (MH "Yoga")	7,717
S39	TI ((breathing N3 exercise*) OR (respiratory N3 muscle N3 training*)) OR AB ((breathing N3 exercise*) OR (respiratory N3 muscle N3 training*)) OR TI (Qigong OR “Qi Gong” OR “Ch’i Kung” OR yoga) OR AB (Qigong OR “Qi Gong” OR “Ch’i Kung” OR yoga)	10,393
S40	(MH "Dance Therapy") OR (MH "Dancing")	3,845
S41	TI (dance OR dancing OR ballet) OR AB (dance OR dancing OR ballet)	7,964
S42	S4 OR S5 OR S6 OR S7 OR S8 OR S9 OR S10 OR S11 OR S12 OR S13 OR S14 OR S15 OR S16 OR S17 OR S18 OR S19 OR S20 OR S21 OR S22 OR S23 OR S24 OR S25 OR S26 OR S27 OR S28 OR S29 OR S30 OR S31 OR S32 OR S33 OR S34 OR S35 OR S36 OR S37 OR S38 OR S39 OR S40 OR S41	1,092,741
	**Cognitive function**	
S43	(MH "Mental Processes") OR (MH "Anticipation, Psychological")	14,781

S44	TI ((mental N3 process*) OR (cognitive N3 process*) OR (information N3 processing)) OR AB ((mental N3 process*) OR (cognitive N3 process*) OR (information N3 processing)) OR TI (psychological N3 (anticipation OR expectation*)) OR AB (psychological N3 (anticipation OR expectation*))	72,824
S45	(MH "Cognition") OR (MH "Awareness") OR (MH "Cognitive Reserve")	146,838
S46	TI (cognition* OR (cognitive N3 (ability OR abilities OR flexibility OR function*)) OR awareness*) OR AB (cognition* OR (cognitive N3 (ability OR abilities OR flexibility OR function*)) OR awareness*) OR TI ((brain OR cognitive) N3 reserve*) OR AB ((brain OR cognitive) N3 reserve*)	389,597
S47	(MH "Comprehension") OR (MH "Consciousness") OR (MH "Imagination") OR (MH "Intuition") OR (MH "Intention")	58,0236
S48	TI (comprehension OR understanding* OR consciousness* OR imagination* OR intuition* OR intention*) OR AB (comprehension OR understanding* OR consciousness* OR imagination* OR intuition* OR intention*)	1,300,707
S49	(MH "Metacognition")	1,795
S50	TI ((cognitive N3 (awareness* OR control* OR knowledge* OR monitoring*)) OR metaemotion* OR meta-emotion* OR metamemory OR meta-memory OR metamemories OR meta-memories OR (meta N3 (emotion* OR memory OR memories))) OR AB ((cognitive N3 (awareness* OR control* OR knowledge* OR monitoring*)) OR metaemotion* OR meta-emotion* OR metamemory OR meta-memory OR metamemories OR meta-memories OR (meta N3 (emotion* OR memory OR memories))) OR TI ((skill* OR training) N3 (carryover OR retain* OR retention*)) OR AB((skill* OR training) N3 (carryover OR retain* OR retention*))	26,290
S51	(MH "Executive Function") OR (MH "Higher Nervous Activity")	21,411
S52	TI (executive N3 (control* OR function*)) OR AB (executive N3 (control* OR function*)) OR TI (higher N3 nervous N3 activit*) OR AB (higher N3 nervous N3 activit*)	40,052
S53	(MH "Learning") OR (MH "Probability Learning") OR (MH "Problem-Based Learning") OR (MH "Discrimination Learning") OR (MH "Self-Directed Learning as Topic") OR (MH "Programmed Instructions as Topic") OR (MH "Spatial Learning") OR (MH "Maze Learning+") OR (MH "Verbal Learning") OR (MH "Serial Learning") OR (MH "Paired-Associate Learning") OR (MH "Association") OR (MH "Association Learning")	162,955
S54	TI (learning* OR phenomenography OR (memory N3 training)) OR AB (learning* OR phenomenography OR (memory N3 training))	444,174
S55	(MH "Conditioning, Psychological")	17,733

S56	TI ((psycholog* N3 conditioning) OR (social N3 learning N3 theor*)) OR AB ((psycholog* N3 conditioning) OR (social N3 learning N3 theor*))	1,159
S57	(MH "Generalization, Psychological") OR (MH "Generalization, Stimulus") OR (MH "Generalization, Response")	5,946
S58	TI ((psycholog* OR response OR stimulus) N3 (generalization* OR generalisation*)) OR AB ((psycholog* OR response OR stimulus) N3 (generalization* OR generalisation*))	1,566
S59	(MH "Memory") OR (MH "Spatial Memory") OR (MH "Retention, Psychology") OR (MH "Repetition Priming") OR (MH "Recognition, Psychology") OR (MH "Mental Recall") OR (MH "Memory, Short-Term") OR (MH "Memory, Long-Term") OR (MH "Memory Consolidation") OR (MH "Memory, Episodic")	153,105
S60	TI (memory OR memories OR ((immediate OR mental) N3 recall*) OR (psycholog* N3 (recognition OR retention)) OR familiarity OR (repetition N3 priming)) OR AB (memory OR memories OR ((immediate OR mental) N3 recall*) OR (psycholog* N3 (recognition OR retention)) OR familiarity OR (repetition N3 priming))	332,927
S61	(MH "Overlearning") OR (MH "Spatial Navigation")	2,153
S62	TI (overlearning*) OR AB (overlearning*) OR TI (spatial N3 (ability OR abilities OR navigation* OR visualization* OR visualization*)) OR AB (spatial N3 (ability OR abilities OR navigation* OR visualization* OR visualization*))	8,304
S63	(MH "Transfer, Psychology")	4,637
S64	TI ((psychology OR learning OR training) N3 transfer*) OR AB ((psychology OR learning OR training) N3 transfer*)	8,861
S65	(MH "Mentalization") OR (MH "Thinking")	18,055
S66	TI (mentalization OR mentalizing) OR AB (mentalization OR mentalizing) OR TI (thinking OR thought*) OR AB (thinking OR thought*)	352,947
S67	(MH "Concept Formation") OR (MH "Creativity")	20,218
S68	TI (conceptualization OR conceptualisation OR (concept* N3 (formation OR learning OR mapping))) OR AB (conceptualization OR conceptualisation OR (concept* N3 (formation OR learning OR mapping))) OR TI (creativity OR creativeness OR (creative N3 (ability OR abilities OR thinking)) OR innovativeness OR originality) OR AB (creativity OR creativeness OR (creative N3 (ability OR abilities OR thinking)) OR innovativeness OR originality)	35,317
S69	(MH "Decision Making") OR (MH "Choice Behavior") OR (MH "Judgment") OR (MH "Heuristics") OR (MH "Problem Solving")	178,485

S70	TI (decision* N3 making) OR AB (decision* N3 making) OR TI ((approach* OR choice*) N3 (behavior* OR behaviour*)) OR AB ((approach* OR choice*) N3 (behavior* OR behaviour*)) OR TI (judgment* OR heuristic*) OR AB (judgment* OR heuristic*) OR TI (brainstorming OR problem* N3 (identify OR identification OR solve* OR solving)) OR AB (brainstorming OR problem* N3 (identify OR identification OR solve* OR solving))	360,794
S71	S43 OR S44 OR S45 OR S46 OR S47 OR S48 OR S49 OR S50 OR S51 OR S52 OR S53 OR S54 OR S55 OR S56 OR S57 OR S58 OR S59 OR S60 OR S61 OR S62 OR S63 OR S64 OR S65 OR S66 OR S67 OR S68 OR S69 OR S70	3,084,124
S72	S3 AND S42 AND S71	32,258
	**Systematic review and/or meta-analysis**	
S73	TI (meta-analysis OR "meta analysis" OR "systematic review") OR AB (meta-analysis OR "meta analysis" OR "systematic review")	360,017
S74	S72 AND S73	1,041
S75	Limiters - Publication Type: Meta-Analysis, Systematic Review	311,082
S76	S72 AND S75	871
S77	S74 OR S76	871
	Search Screen - Advanced Search<br>Search modes - Boolean/Phrase	

PsycInfo (ProQuest) n = 1249

Date of final search: 30 May 2023
Years of coverage: 1806 – 2023

(ti(meta-analysis OR "meta analysis" OR "systematic review") OR ab(meta-analysis OR "meta analysis" OR "systematic review")) AND ((MAINSUBJECT.EXACT("Older Adulthood") OR MAINSUBJECT.EXACT("Middle Adulthood")) OR (aged OR ageing OR aging OR elder* OR (old* NEAR/3 (adult* OR person* OR people)) OR senior* OR centenarian* OR nonagenarian* OR octogenarian* OR "oldest old" OR "old age" OR "middle age" OR "middle adulthood" OR geriatric* OR gerontolog*)) AND ((MAINSUBJECT.EXACT("Exercise") OR MAINSUBJECT.EXACT("Aerobic Exercise") OR MAINSUBJECT.EXACT("Physical Activity") OR MAINSUBJECT.EXACT("Physical Strength") OR MAINSUBJECT.EXACT("Weightlifting") OR MAINSUBJECT.EXACT("Walking") OR MAINSUBJECT.EXACT("Running") OR MAINSUBJECT.EXACT("Swimming") OR MAINSUBJECT.EXACT("Sports") OR MAINSUBJECT.EXACT("Extreme Sports") OR MAINSUBJECT.EXACT("Professional Sports") OR MAINSUBJECT.EXACT("Athletic Performance") OR MAINSUBJECT.EXACT("Physical Endurance") OR MAINSUBJECT.EXACT("Physical Fitness") OR MAINSUBJECT.EXACT("Baseball") OR MAINSUBJECT.EXACT("Basketball") OR MAINSUBJECT.EXACT("Football") OR MAINSUBJECT.EXACT("Tennis") OR MAINSUBJECT.EXACT("Soccer") OR MAINSUBJECT.EXACT("Martial Arts") OR

MAINSUBJECT.EXACT("Judo") OR MAINSUBJECT.EXACT("Yoga") OR MAINSUBJECT.EXACT("Dance") OR MAINSUBJECT.EXACT("Dance Therapy")) OR (((aerobic OR acute OR isometric OR physical) NEAR/3 (activit* OR conditioning OR exercise* OR fitness OR training*)) OR ((cool-down OR cooldown OR (cool NEAR/3 down) OR cooling-down OR recovery OR warm-down OR (warm NEAR/3 down) OR warming-down OR warm-up OR warmup OR warming-up OR (warm* NEAR/3 up)) NEAR/3 exercise*) OR gymnastics OR calisthenics OR ((muscle* OR active OR ballistic OR dynamic OR isometric OR passive OR PNF OR "Proprioceptive Neuromuscular Facilitation*" OR relaxed OR static OR static-active OR (static NEAR/3 active) OR static-passive OR (static NEAR/3 passive)) NEAR/3 stretching) OR (human NEAR/3 (conditioning OR training) NEAR/3 physical) OR ((circuit OR circuit-based) NEAR/3 (exercise* OR training*)) OR (endurance NEAR/3 (exercise* OR training*)) OR ((high-intensity OR (high NEAR/3 intensity) OR interval OR intermittent OR sprint) NEAR/3 (exercise* OR interval* OR training*)) OR ((plyometric OR cycle OR stretch-shortening OR (stretch NEAR/3 shortening)) NEAR/3 (drill* OR exercise* OR training*)) OR ((resistance OR strength OR strengthening OR weight-bearing OR weight-lifting OR (weight* NEAR/3 (bear* OR lift*))) NEAR/3 (activit* OR conditioning OR exercise* OR training* OR program*)) OR run OR running OR runnings OR jog OR jogging OR joggings OR marathon* OR ultramarathon* OR swim* OR swimming OR walk* OR walking OR (stair* NEAR/3 (climbing OR navigation)) OR sport* OR athletic* OR (cardiorespiratory NEAR/3 fitness) OR (physical NEAR/3 (endurance OR fitness OR stamina)) OR ((physical OR functional) NEAR/3 performance*) OR baseball* OR softball* OR basketball* OR netball* OR bike* OR bicycl* OR boxing* OR cricket OR football* OR rugby OR rugbies OR golf* OR hockey* OR mountaineering* OR Aikido OR "Hap Ki Do" OR Judo OR Karate OR Jujitsu OR (martial NEAR/3 arts*) OR "Kung Fu" OR (gong NEAR/3 fu) OR Gongfu OR "Tae Kwon Do" OR Wushu OR Tai-Ji OR (Tai NEAR/3 Chi) OR (Tai NEAR/3 Ji NEAR/3 Quan) OR Taiji OR Taijiquan OR "T'ai Chi" OR badminton OR lacrosse OR racketball OR racquetball OR ((racquet OR racket OR squash) NEAR/3 ball) OR tennis OR skating* OR skateboarding* OR soccer* OR ((return OR resumption*) NEAR/3 (play OR recreational)) OR snowmobiling OR sledding OR snowboarding OR skiing* OR parathletic* OR para-athletic* OR para-sport* OR track* OR "Field and Track" OR volleyball* OR boating OR canoeing OR diving* OR kayaking OR rowing OR surfing OR surfboarding OR (water NEAR/3 polo) OR (water NEAR/3 skiing) OR (weight NEAR/3 lifting*) OR wrestling* OR (exercise NEAR/3 movement NEAR/3 (technics OR technique*)) OR pilates OR pilates-based OR (breathing NEAR/3 exercise*) OR (respiratory NEAR/3 muscle NEAR/3 training*) OR Qigong OR "Qi Gong" OR "Ch'i Kung" OR yoga OR dance OR dancing OR ballet)) AND ((MAINSUBJECT.EXACT("Cognitive Processes") OR MAINSUBJECT.EXACT("Cognition") OR MAINSUBJECT.EXACT("Cognitive Ability") OR MAINSUBJECT.EXACT("Cognitive Flexibility") OR MAINSUBJECT.EXACT("Awareness") OR MAINSUBJECT.EXACT("Cognitive Reserve") OR MAINSUBJECT.EXACT.EXPLODE("Comprehension") OR MAINSUBJECT.EXACT("Consciousness States") OR MAINSUBJECT.EXACT("Imagination") OR MAINSUBJECT.EXACT("Intuition") OR MAINSUBJECT.EXACT("Intention") OR MAINSUBJECT.EXACT("Metacognition") OR MAINSUBJECT.EXACT.EXPLODE("Executive Function") OR MAINSUBJECT.EXACT("Learning") OR MAINSUBJECT.EXACT("Discrimination Learning") OR MAINSUBJECT.EXACT("Probability Learning") OR MAINSUBJECT.EXACT("Probability Judgment") OR MAINSUBJECT.EXACT("Problem Based Learning") OR MAINSUBJECT.EXACT("Self-Regulated Learning") OR MAINSUBJECT.EXACT.EXPLODE("Serial Learning") OR MAINSUBJECT.EXACT("Spatial Learning") OR MAINSUBJECT.EXACT.EXPLODE("Verbal Learning") OR MAINSUBJECT.EXACT("Conditioning") OR MAINSUBJECT.EXACT("Cognitive Generalization") OR MAINSUBJECT.EXACT.EXPLODE("Generalization (Learning)") OR MAINSUBJECT.EXACT("Memory") OR MAINSUBJECT.EXACT("Explicit Memory") OR MAINSUBJECT.EXACT("Associative Memory") OR MAINSUBJECT.EXACT("Memory Trace") OR MAINSUBJECT.EXACT.EXPLODE("Spatial Memory") OR MAINSUBJECT.EXACT.EXPLODE("Semantic Memory") OR MAINSUBJECT.EXACT.EXPLODE("Visual Memory") OR MAINSUBJECT.EXACT("Verbal Memory") OR MAINSUBJECT.EXACT("Memory Consolidation") OR MAINSUBJECT.EXACT("Episodic Memory") OR MAINSUBJECT.EXACT("Short Term Memory")

OR MAINSUBJECT.EXACT("Long Term Memory") OR MAINSUBJECT.EXACT.EXPLODE("Retention") OR MAINSUBJECT.EXACT("Overlearning") OR MAINSUBJECT.EXACT("Transfer (Learning)") OR MAINSUBJECT.EXACT.EXPLODE("Spatial Ability") OR MAINSUBJECT.EXACT("Mentalization") OR MAINSUBJECT.EXACT("Thinking") OR MAINSUBJECT.EXACT.EXPLODE("Concept Formation") OR MAINSUBJECT.EXACT("Creativity") OR MAINSUBJECT.EXACT("Decision Making") OR MAINSUBJECT.EXACT("Problem Solving") OR MAINSUBJECT.EXACT.EXPLODE("Choice Behavior") OR MAINSUBJECT.EXACT("Judgment") OR MAINSUBJECT.EXACT("Heuristics")) OR ((mental NEAR/3 process*) OR (cognitive NEAR/3 process*) OR (information NEAR/3 processing) OR (psychological NEAR/3 (anticipation OR expectation*)) OR cognition* OR (cognitive NEAR/3 (ability OR abilities OR flexibility OR function*)) OR awareness* OR ((brain OR cognitive) NEAR/3 reserve*) OR comprehension OR understanding* OR consciousness* OR imagination* OR intention* OR intuition* OR (executive NEAR/3 (control* OR function*)) OR (higher NEAR/3 nervous NEAR/3 activit*) OR meta-cognition OR metacognition OR (meta NEAR/3 cognition) OR ((metacognitive OR meta-cognitive OR (meta NEAR/3 cognitive)) NEAR/3 (awareness* OR control* OR knowledge* OR monitoring*)) OR metaemotion* OR meta-emotion* OR metamemory OR meta-memory OR metamemories OR meta-memories OR (meta NEAR/3 (emotion* OR memory OR memories)) OR ((skill* OR training) NEAR/3 (carryover OR retain* OR retention*)) OR learning* OR phenomenography OR (psycholog* NEAR/3 conditioning) OR (social NEAR/3 learning NEAR/3 theor*) OR ((psycholog* OR response OR stimulus) NEAR/3 (generalization* OR generalisation*)) OR memory OR memories OR ((immediate OR mental) NEAR/3 recall*) OR (psycholog* NEAR/3 (recognition OR retention)) OR familiarity OR (repetition NEAR/3 priming) OR overlearning* OR (spatial NEAR/3 (ability OR abilities OR navigation* OR visualization* OR visualization*)) OR ((psychology OR learning OR training) NEAR/3 transfer*) OR mentalization OR mentalizing OR thinking OR thought* OR conceptualization OR conceptualisation OR (concept* NEAR/3 (formation OR learning)) OR creativity OR creativeness OR (creative NEAR/3 (ability OR abilities OR thinking)) OR innovativeness OR originality OR (decision* NEAR/3 making) OR ((approach* OR choice*) NEAR/3 (behavior* OR behaviour*)) OR judgment* OR heuristic* OR brainstorming OR (problem* NEAR/3 (identify OR identification OR solve* OR solving))))

Limits applied: Peer-reviewed

Scopus (Elsevier) n = 3,382

Date of final search: 30 May 2023
Years of coverage: 1788 – 2023

(TITLE-ABS (meta-analysis OR "meta analysis" OR "systematic review")) AND (TITLE-ABS-KEY (aged OR ageing OR aging OR elder* OR (old* W/3 (adult* OR person* OR people)) OR senior* OR centenarian* OR nonagenarian* OR octogenarian* OR "oldest old" OR "old age" OR "middle age" OR "middle adulthood" OR geriatric* OR gerontolog*)) AND (TITLE-ABS-KEY (((aerobic OR acute OR isometric OR physical) W/3 (activit* OR conditioning OR exercise* OR fitness OR training*)) OR ((cool-down OR cooldown OR (cool W/3 down) OR cooling-down OR recovery OR warm-down OR (warm W/3 down) OR warming-down OR warm-up OR warmup OR warming-up OR (warm* W/3 up)) W/3 exercise*) OR gymnastics OR calisthenics OR ((muscle* OR active OR ballistic OR dynamic OR isometric OR passive OR pnf OR "Proprioceptive Neuromuscular Facilitation*" OR relaxed OR static OR static-active OR (static W/3 active) OR static-passive OR (static W/3 passive)) W/3 stretching) OR (human W/3 (conditioning OR training) W/3 physical) OR ((circuit OR circuit-based) W/3 (exercise* OR training*)) OR (endurance W/3 (exercise* OR training*)) OR ((high-intensity OR (high W/3 intensity) OR interval OR intermittent OR sprint) W/3 (exercise* OR interval* OR training*)) OR ((plyometric OR cycle OR stretch-shortening OR (stretch W/3 shortening)) W/3 (drill* OR exercise* OR training*)) OR ((resistance OR strength OR strengthening OR weight-bearing OR weight-lifting OR (weight* W/3 (bear* OR lift*))) W/3 (activit* OR conditioning OR

exercise* OR training* OR program*)) OR run OR running OR runnings OR jog OR jogging OR joggings OR marathon* OR ultramarathon* OR swim* OR swimming OR walk* OR walking OR (stair* W/3 (climbing OR navigation)) OR sport* OR athletic* OR (cardiorespiratory W/3 fitness) OR (physical W/3 (endurance OR fitness OR stamina)) OR ((physical OR functional) W/3 performance*) OR baseball* OR softball* OR basketball* OR netball* OR bike* OR bicycl* OR boxing* OR cricket OR football* OR rugby OR rugbies OR golf* OR hockey* OR mountaineering* OR aikido OR "Hap Ki Do" OR judo OR karate OR jujitsu OR (martial W/3 arts*) OR "Kung Fu" OR (gong W/3 fu) OR gongfu OR "Tae Kwon Do" OR wushu OR tai-ji OR (tai W/3 chi) OR (tai W/3 ji W/3 quan) OR taiji OR taijiquan OR "T'ai Chi" OR badminton OR lacrosse OR racketball OR racquetball OR ((racquet OR racket OR squash) W/3 ball) OR tennis OR skating* OR skateboarding* OR soccer* OR ((return OR resumption*) W/3 (play OR recreational)) OR snowmobiling OR sledding OR snowboarding OR skiing* OR parathletic* OR para-athletic* OR para-sport* OR track* OR "Field and Track" OR volleyball* OR boating OR canoeing OR diving* OR kayaking OR rowing OR surfing OR surfboarding OR (water W/3 polo) OR (water W/3 skiing) OR (weight W/3 lifting*) OR wrestling* OR (exercise W/3 movement W/3 (technics OR technique*)) OR pilates OR pilates-based OR (breathing W/3 exercise*) OR (respiratory W/3 muscle W/3 training*) OR qigong OR "Qi Gong" OR "Ch'i Kung" OR yoga OR dance OR dancing OR ballet)) AND (TITLE-ABS-KEY ((mental W/3 process*) OR (cognitive W/3 process*) OR (information W/3 processing) OR (psychological W/3 (anticipation OR expectation*)) OR cognition* OR (cognitive W/3 (ability OR abilities OR flexibility OR function*)) OR awareness* OR ((brain OR cognitive) W/3 reserve*) OR comprehension OR understanding* OR consciousness* OR imagination* OR intention* OR intuition* OR (executive W/3 (control* OR function*)) OR (higher W/3 nervous W/3 activit*) OR meta-cognition OR metacognition OR (meta W/3 cognition) OR ((metacognitive OR meta-cognitive OR (meta W/3 cognitive)) W/3 (awareness* OR control* OR knowledge* OR monitoring*)) OR metaemotion* OR meta-emotion* OR metamemory OR meta-memory OR metamemories OR meta-memories OR (meta W/3 (emotion* OR memory OR memories)) OR ((skill* OR training) W/3 (carryover OR retain* OR retention*)) OR learning* OR phenomenography OR (psycholog* W/3 conditioning) OR (social W/3 learning W/3 theor*) OR ((psycholog* OR response OR stimulus) W/3 (generalization* OR generalisation*)) OR memory OR memories OR ((immediate OR mental) W/3 recall*) OR (psycholog* W/3 (recognition OR retention)) OR familiarity OR (repetition W/3 priming) OR overlearning* OR (spatial W/3 (ability OR abilities OR navigation* OR visualization* OR visualization*)) OR ((psychology OR learning OR training) W/3 transfer*) OR mentalization OR mentalizing OR thinking OR thought* OR conceptualization OR conceptualisation OR (concept* W/3 (formation OR learning)) OR creativity OR creativeness OR (creative W/3 (ability OR abilities OR thinking)) OR innovativeness OR originality OR (decision* W/3 making) OR ((approach* OR choice*) W/3 (behavior* OR behaviour*)) OR judgment* OR heuristic* OR brainstorming OR (problem* W/3 (identify OR identification OR solve* OR solving)))))

Web of Science Core Collection n = 2,515

Date of final search: 30 May 2023
Years of coverage: 1945 – 2023

1	meta-analysis OR "meta analysis" OR "systematic review" (Title) or meta-analysis OR "meta analysis" OR "systematic review" (Abstract)	333,710
2	TS=(aged OR ageing OR aging OR elder* OR (old* NEAR/3 (adult* OR person* OR people)) OR senior* OR centenarian* OR nonagenarian* OR octogenarian* OR “oldest old” OR “old age” OR “middle age” OR “middle adulthood” OR geriatric* OR gerontolog*) | Exact search	1,600,116
3	TS=(((aerobic OR acute OR isometric OR physical) NEAR/3 (activit* OR conditioning OR exercise* OR fitness OR training*)) OR ((cool-down OR	2,761,118

	cooldown OR (cool NEAR/3 down) OR cooling-down OR recovery OR warm-down OR (warm NEAR/3 down) OR warming-down OR warm-up OR warmup OR warming-up OR (warm* NEAR/3 up)) NEAR/3 exercise*) OR gymnastics OR calisthenics OR ((muscle* OR active OR ballistic OR dynamic OR isometric OR passive OR PNF OR "Proprioceptive Neuromuscular Facilitation*" OR relaxed OR static OR static-active OR (static NEAR/3 active) OR static-passive OR (static NEAR/3 passive)) NEAR/3 stretching) OR (human NEAR/3 (conditioning OR training) NEAR/3 physical) OR ((circuit OR circuit-based) NEAR/3 (exercise* OR training*)) OR (endurance NEAR/3 (exercise* OR training*)) OR ((high-intensity OR (high NEAR/3 intensity) OR interval OR intermittent OR sprint) NEAR/3 (exercise* OR interval* OR training*)) OR ((plyometric OR cycle OR stretch-shortening OR (stretch NEAR/3 shortening)) NEAR/3 (drill* OR exercise* OR training*)) OR ((resistance OR strength OR strengthening OR weight-bearing OR weight-lifting OR (weight* NEAR/3 (bear* OR lift*))) NEAR/3 (activit* OR conditioning OR exercise* OR training* OR program*)) OR run OR running OR runnings OR jog OR jogging OR joggings OR marathon* OR ultramarathon* OR swim* OR swimming OR walk* OR walking OR (stair* NEAR/3 (climbing OR navigation)) OR sport* OR athletic* OR (cardiorespiratory NEAR/3 fitness) OR (physical NEAR/3 (endurance OR fitness OR stamina)) OR ((physical OR functional) NEAR/3 performance*) OR baseball* OR softball* OR basketball* OR netball* OR bike* OR bicycl* OR boxing* OR cricket OR football* OR rugby OR rugbies OR golf* OR hockey* OR mountaineering* OR Aikido OR "Hap Ki Do" OR Judo OR Karate OR Jujitsu OR (martial NEAR/3 arts*) OR "Kung Fu" OR (gong NEAR/3 fu) OR Gongfu OR "Tae Kwon Do" OR Wushu OR Tai-Ji OR (Tai NEAR/3 Chi) OR (Tai NEAR/3 Ji NEAR/3 Quan) OR Taiji OR Taijiquan OR "T'ai Chi" OR badminton OR lacrosse OR racketball OR racquetball OR ((racquet OR racket OR squash) NEAR/3 ball) OR tennis OR skating* OR skateboarding* OR soccer* OR ((return OR resumption*) NEAR/3 (play OR recreational)) OR snowmobiling OR sledding OR snowboarding OR skiing* OR parathletic* OR para-athletic* OR para-sport* OR track* OR "Field and Track" OR volleyball* OR boating OR canoeing OR diving* OR kayaking OR rowing OR surfing OR surfboarding OR (water NEAR/3 polo) OR (water NEAR/3 skiing) OR (weight NEAR/3 lifting*) OR wrestling* OR (exercise NEAR/3 movement NEAR/3 (technics OR technique*)) OR pilates OR pilates-based OR (breathing NEAR/3 exercise*) OR (respiratory NEAR/3 muscle NEAR/3 training*) OR Qigong OR "Qi Gong" OR "Ch'i Kung" OR yoga OR dance OR dancing OR ballet) | Exact search	
4	TS=((mental NEAR/3 process*) OR (cognitive NEAR/3 process*) OR (information NEAR/3 processing) OR (psychological NEAR/3 (anticipation OR expectation*)) OR cognition* OR (cognitive NEAR/3 (ability OR abilities OR flexibility OR function*)) OR awareness* OR ((brain OR cognitive) NEAR/3 reserve*) OR comprehension OR understanding* OR consciousness* OR imagination* OR intention* OR intuition* OR (executive NEAR/3 (control* OR function*)) OR (higher NEAR/3 nervous NEAR/3 activit*) OR meta-cognition OR metacognition OR (meta NEAR/3 cognition) OR ((metacognitive OR meta-cognitive OR (meta NEAR/3 cognitive)) NEAR/3 (awareness* OR control* OR knowledge* OR monitoring*)) OR metaemotion* OR meta-emotion* OR metamemory OR meta-memory OR metamemories OR meta-memories OR (meta NEAR/3 (emotion* OR memory OR memories)) OR ((skill* OR training) NEAR/3 (carryover OR retain* OR retention*)) OR learning* OR phenomenography (psycholog* NEAR/3 conditioning) OR (social NEAR/3 learning NEAR/3 theor*) OR ((psycholog* OR response OR stimulus) NEAR/3 (generalization* OR generalisation*)) OR memory OR memories OR ((immediate OR mental) NEAR/3 recall*) OR (psycholog* NEAR/3 (recognition OR retention)) OR familiarity OR (repetition NEAR/3 priming) OR overlearning* OR (spatial NEAR/3 (ability OR abilities OR navigation* OR visualization* OR visualization*)) OR ((psychology OR learning OR training) NEAR/3	5,749,561

	transfer*) OR mentalization OR mentalizing OR thinking OR thought* OR conceptualization OR conceptualisation OR (concept* NEAR/3 (formation OR learning)) OR creativity OR creativeness OR (creative NEAR/3 (ability OR abilities OR thinking)) OR innovativeness OR originality OR (decision* NEAR/3 making) OR ((approach* OR choice*) NEAR/3 (behavior* OR behaviour*)) OR judgment* OR heuristic* OR brainstorming OR (problem* NEAR/3 (identify OR identification OR solve* OR solving))) | Exact search	
5	(((#1) AND #2) AND #3) AND #4	2,515
	Timespan: All years.<br>Indexes: SCI-EXPANDED, SSCI, A&HCI, CPCI-S, CPCI-SSH, ESCI.	
