# Supplementary material for: Mind body exercise improves cognitive function more than aerobic- and resistance exercise in healthy adults aged 55 years and older – an umbrella review
Source: Eur Rev Aging Phys Act. 2023 Aug 9;20:15. doi: 10.1186/s11556-023-00325-4 (PMC10413530; doi:10.1186/s11556-023-00325-4)
Supplement: Supplementary file 2 — Additional file 2: Supplement S2. Characteristics of excluded studies. [file 11556_2023_325_MOESM2_ESM.pdf]

Supplement S2. Characteristics of excluded studies

Author, year, referens	Reason for exclusion, wrong
Acevedo et al, 2016 (1)	outcome
Ahlskog et al, 2011 (2)	design
Ahn et al, 2021 (3)	intervention
Ai et al, 2021 (4)	participants
Al-Yahya et al, 2011 (5)	intervention
Amagasa et al, 2018 (6)	outcome
Anderson et al, 2014 (7)	design
Araque-Martínez et al, 2021 (8)	design
Barha et al, 2017 (9)	participants
Barreto et al, 2018 (10)	design
Bauman et al, 2016 (11)	design
Beckett et al, 2015 (12)	intervention
Bediou et al, 2018 (13)	intervention
Beydoun et al, 2014 (14)	design
Bleakley et al, 2015 (15)	outcome
Blondell et al, 2014 (16)	intervention
Bouaziz et al, 2016 (17)	participants
Bouaziz et al, 2017 (18)	participants
Brasure et al, 2018 (19)	design
Bray et al, 2021 (20)	outcome
Bruderer-Hofstetter et al, 2018 (21)	intervention
Campos et al, 2021 (22)	outcome
Carvalho et al, 2014 (23)	outcome
Catchlove et al, 2018 (24)	outcome
Chan et al, 2019 (25)	participants
Chang et al, 2017 (26)	design
Chang et al, 2012 (27)	design
Chen et al, 2020 (28)	outcome
Chen et al, 2021 (29)	participants
Cheng et al, 2022 (30)	design
Chobe et al, 2020 (31)	participants
Clouston et al, 2013 (32)	design
Coelho-Júnior. et al, 2021(33)	design
Colcombe et al, 2003 (34)	participants
Conti et al, 2009 (35)	intervention
Cooper et al, 2011 (36)	design
Cordes et al, 2021 (37)	participants
Cunningham et al, 2020 (38)	design
Cunningham et al, 2018 (39)	design
Cunningham et al, 2020 (40)	design
Daviglus et al, 2011 (41)	intervention
de Assis et al, 2017 (42)	participants
de Keijzer et al, 2020 (43)	intervention
De Silva et al, 2019 (44)	intervention
de Sousa et al, 2018 (45)	participants
Dedeyne et al, 2017 (46)	intervention
Erlenbach et al, 2021 (47)	design
Etnier et al, 2006 (48)	intervention
Gallardo-Gomez et al, 2022 (49)	participants
Gallou-Guyot et al, 2020 (50)	intervention

Gavelin et al, 2021 (51)	intervention
Gheysen et al, 2018 (52)	intervention
Gomes-Osman et al, 2018 (53)	design
Gothe et al, 2015 (54)	participants
Griebler et al 2022 (55)	design
Gu et al, 2019 (56)	design
Guimarães et al, 2014 (57)	design
Guo et al, 2020 (58)	intervention
Hajar et al, 2019 (59)	participants
Han et al 2022 (60)	participants
Hatchard et al, 2014 (61)	participants
Herold et al, 2019 (62)	outcome
Herold et al 2023 (63)	design
Hewston et al, 2021 (64)	participants
Hindin et al, 2009 (65)	design
Hoffmann et al, 2021 (66)	participants
Howes et al, 2017 (67)	participants
Hoy et al, 2021 (68)	design
Hsieh et al, 2021 (69)	design
Intzandt et al, 2021 (70)	design
Jedrziewski et al, 2007 (71)	design
Ji et al, 2021 (72)	design
Joubert et al, 2018 (73)	design
Jung et al, 2020 (74)	intervention
Karr et al, 2014 (75)	participants
Kelly et al, 2014 (76)	participants
Koch et al, 2019 (77)	participants
Kramer et al, 2003 (78)	design
Kramer et al, 2018 (79)	design
Kramer et al, 2007 (80)	design
Laird et al, 2018 (81)	design
Lanrigan et al, 2020 (82)	participants
Lauenroth et al, 2016 (83)	design
Law et al, 2014 (84)	intervention
Lee et al, 2010 (85)	design
Lehert et al, 2015 (86)	intervention
Lei et al, 2019 (87)	design
Leung et al, 2007 (88)	design
Levin et al, 2017 (89)	intervention
Li et al, 2018 (90)	design
Li et al, 2022 (91)	intervention
Li et al, 2023 (92)	intervention
Lipnicki et al, 2019 (93)	design
Liu et al, 2021 (94)	participants
Liu et al, 2021 (95)	intervention
Loprinzi et al, 2018 (96)	design
Ludyga et al, 2016 (97)	participants
Ludyga et al, 2020 (98)	participants
Luu et al, 2016 (99)	design
Lü et al, 2016 (100)	design
Mansor et al, 2020 (101)	participants
McMorris et al, 2012 (102)	participants
McSween et al, 2019 (103)	design

Meng et al, 2020 (104)	design
Menichetti et al, 2016 (105)	design
Miller et al, 2014 (106)	design
Miller et al, 2014 (107)	design
Miller et al, 2016 (108)	design
Morat et al, 2021 (109)	intervention
Moreau et al, 2019 (110)	participants
Muiños et al, 2021 (111)	design
Northey et al, 2018 (112)	participants
Oberste et al, 2019 (113)	participants
Oberste et al, 2021 (114)	participants
Ogawa et al, 2016 (115)	design
Olanrewaju et al, 2016 (116)	design
Olanrewaju et al, 2020 (117)	intervention
Origua Rios et al, 2018 (118)	intervention
Patel et al, 2012 (119)	design
Paterson et al, 2010 (120)	design
Pessoa et al, 2019 (121)	design
Predovan et al, 2019 (122)	design
Ramsey et al, 2021 (123)	design
Rathore et al, 2017 (124)	intervention
Ren et al, 2021 (125)	participants
Roberts et al, 2017 (126)	outcome
Rojer et al, 2021 (127)	design
Russo et al, 2020 (128)	language
Saez de Asteasu et al, 2017 (129)	design
Sakaki et al, 2021 (130)	design
Sanders et al, 2019 (131)	participants
Sexton et al, 2019 (132)	participants
Silva et al, 2017 (133)	design
Smith et al, 2010 (134)	participants
Snowden et al, 2011 (135)	design
Soares et al, 2021 (136)	intervention
Sofi et al, 2011 (137)	intervention
Solloway et al, 2016 (138)	participants
Stanmore et al, 2017 (149)	participants
Stein et al, 2018 (140)	design
Stojan et al, 2019 (141)	design
Teixeira-Machado et al, 2019 (142)	design
Tseng et al, 2011 (143)	design
Turner et al, 2021 (144)	design
van Uffelen et al, 2008 (145)	design
Vaportzis et al, 2019 (146)	intervention
Wang et al, 2016 (147)	intervention
Wang et al, 2021 (148)	participants
Wayne et al, 2014 (149)	participants
Whitty et al, 2020 (150)	design
Wilke et al, 2019 (151)	participants
Williams et al, 2010 (152)	design
Wollesen et al, 2020 (153)	intervention
Wu et al, 2019 (154)	participants
Wu et al, 2021 (155)	participants
Wu et al, 2013 (156)	participants

Xu et al, 2020 (157)	intervention
Xu et al, 2023 (158)	participants
Yang et al, 2021 (159)	outcome
Yang et al, 2021 (160)	intervention
Yen et al, 2021(161)	participants
Yoneda et al, 2021 (162)	design
Young et al, 2015 (163)	intervention
Zhang et al, 2016 (164)	intervention
Zheng et al, 2015 (165)	design
Zheng et al, 2021 (166)	design
Zheng et al, 2022 (167)	design
Zhidong et al, 2021 (168)	participants
Zhu et al, 2020 (169)	design
Zhu et al, 2016 (170)	intervention
Zou et al, 2020 (171)	design
Zou et al, 2018 (172)	design
Zülke et al, 2023 (173)	design

1. Acevedo BP, Pospos S, Lavretsky H. The Neural Mechanisms of Meditative Practices: Novel Approaches for Healthy Aging. Current behavioral neuroscience reports. 2016;3(4):328-39.
2. Ahlskog JE, Geda YE, Graff-Radford NR, Petersen RC. Physical exercise as a preventive or disease-modifying treatment of dementia and brain aging. Mayo Clin Proc. 2011;86(9):876-84.
3. Ahn S, Chung JW, Crane MK, Bassett DR, Jr., Anderson JG. The Effects of Multi-Domain Interventions on Cognition: A Systematic Review. West J Nurs Res. 2022;44(12):1134-54.
4. Ai JY, Chen FT, Hsieh SS, Kao SC, Chen AG, Hung TM, et al. The Effect of Acute High-Intensity Interval Training on Executive Function: A Systematic Review. Int J Environ Res Public Health. 2021;18(7).
5. Al-Yahya E, Dawes H, Smith L, Dennis A, Howells K, Cockburn J. Cognitive motor interference while walking: a systematic review and meta-analysis. Neurosci Biobehav Rev. 2011;35(3):715-28.
6. Amagasa S, Machida M, Fukushima N, Kikuchi H, Takamiya T, Odagiri Y, et al. Is objectively measured light-intensity physical activity associated with health outcomes after adjustment for moderate-to-vigorous physical activity in adults? A systematic review. Int J Behav Nutr Phys Act. 2018;15(1):65.
7. Anderson D, Seib C, Rasmussen L. Can physical activity prevent physical and cognitive decline in postmenopausal women? A systematic review of the literature. Maturitas. 2014;79(1):14-33.
8. Araque-Martínez MÁ, Artés-Rodríguez EM, Ruiz-Montero PJ, Casimiro-Andújar AJ. Physical, cognitive and emotional outcomes in older adults exercisers: A systematic review. 2021.
9. Barha CK, Davis JC, Falck RS, Nagamatsu LS, Liu-Ambrose T. Sex differences in exercise efficacy to improve cognition: A systematic review and meta-analysis of randomized controlled trials in older humans. Front Neuroendocrinol. 2017;46:71-85.
10. de Souto Barreto P, Demougeot L, Vellas B, Rolland Y. Exercise Training for Preventing Dementia, Mild Cognitive Impairment, and Clinically Meaningful Cognitive Decline: A Systematic Review and Meta-analysis. J Gerontol A Biol Sci Med Sci. 2018;73(11):1504-11.
11. Bauman A, Merom D, Bull FC, Buchner DM, Fiatarone Singh MA. Updating the evidence for physical activity: summative reviews of the epidemiological evidence, prevalence, and interventions to promote “active aging”. The gerontologist. 2016;56(Suppl_2):S268-S80.

12. Beckett MW, Ardern CI, Rotondi MA. A meta-analysis of prospective studies on the role of physical activity and the prevention of Alzheimer's disease in older adults. BMC Geriatr. 2015;15(1):1-7.
13. Bediou B, Adams DM, Mayer RE, Tipton E, Green CS, Bavelier D. Meta-analysis of action video game impact on perceptual, attentional, and cognitive skills. Psychol Bull. 2018;144(1):77.
14. Beydoun MA, Beydoun HA, Gamaldo AA, Teel A, Zonderman AB, Wang Y. Epidemiologic studies of modifiable factors associated with cognition and dementia: systematic review and meta-analysis. BMC Public Health. 2014;14:643.
15. Bleakley CM, Charles D, Porter-Armstrong A, McNeill MD, McDonough SM, McCormack B. Gaming for health: a systematic review of the physical and cognitive effects of interactive computer games in older adults. J Appl Gerontol. 2015;34(3):Np166-89.
16. Blondell SJ, Hammersley-Mather R, Veerman JL. Does physical activity prevent cognitive decline and dementia?: A systematic review and meta-analysis of longitudinal studies. BMC Public Health. 2014;14:510.
17. Bouaziz W, Lang PO, Schmitt E, Kaltenbach G, Geny B, Vogel T. Health benefits of multicomponent training programmes in seniors: a systematic review. Int J Clin Pract. 2016;70(7):520-36.
18. Bouaziz W, Vogel T, Schmitt E, Kaltenbach G, Geny B, Lang PO. [Health benefits of aerobic training programs in adults aged 70 or over: A systematic review]. Presse Med. 2017;46(9):794-807.
19. Brasure M, Desai P, Davila H, Nelson VA, Calvert C, Jutkowitz E, et al. Physical Activity Interventions in Preventing Cognitive Decline and Alzheimer-Type Dementia: A Systematic Review. Annals of internal medicine. 2018;168(1):30-8.
20. Bray NW, Pieruccini-Faria F, Bartha R, Doherty TJ, Nagamatsu LS, Montero-Odasso M. The effect of physical exercise on functional brain network connectivity in older adults with and without cognitive impairment. A systematic review. Mech Ageing Dev. 2021;196:111493.
21. Bruderer-Hofstetter M, Rausch-Osthoff AK, Meichtry A, Münzer T, Niedermann K. Effective multicomponent interventions in comparison to active control and no interventions on physical capacity, cognitive function and instrumental activities of daily living in elderly people with and without mild impaired cognition - A systematic review and network meta-analysis. Ageing research reviews. 2018;45:1-14.
22. Campos DM, Ferreira DL, Gonçalves GH, Farche ACS, de Oliveira JC, Ansai JH. Effects of aquatic physical exercise on neuropsychological factors in older people: A systematic review. Arch Gerontol Geriatr. 2021;96:104435.
23. Carvalho A, Rea IM, Parimon T, Cusack BJ. Physical activity and cognitive function in individuals over 60 years of age: a systematic review. Clin Interv Aging. 2014;9:661-82.
24. Catchlove SJ, Pipingas A, Hughes ME, Macpherson H. Magnetic resonance imaging for assessment of cerebrovascular reactivity and its relationship to cognition: a systematic review. BMC Neurosci. 2018;19(1):21.
25. Chan JSY, Deng K, Wu J, Yan JH. Effects of Meditation and Mind-Body Exercises on Older Adults' Cognitive Performance: A Meta-analysis. Gerontologist. 2019;59(6):e782-e90.
26. Chang YK, Chu CH, Chen FT, Hung TM, Etnier JL. Combined Effects of Physical Activity and Obesity on Cognitive Function: Independent, Overlapping, Moderator, and Mediator Models. Sports Med. 2017;47(3):449-68.
27. Chang YK, Labban JD, Gapin JI, Etnier JL. The effects of acute exercise on cognitive performance: a meta-analysis. Brain Res. 2012;1453:87-101.
28. Chen FT, Hopman RJ, Huang CJ, Chu CH, Hillman CH, Hung TM, et al. The Effect of Exercise Training on Brain Structure and Function in Older Adults: A Systematic Review Based on Evidence from Randomized Control Trials. Journal of clinical medicine. 2020;9(4).

29. Chen ML, Wotiz SB, Banks SM, Connors SA, Shi Y. Dose-Response Association of Tai Chi and Cognition among Community-Dwelling Older Adults: A Systematic Review and Meta-Analysis. Int J Environ Res Public Health. 2021;18(6).
30. Cheng A, Zhao Z, Liu H, Yang J, Luo J. The physiological mechanism and effect of resistance exercise on cognitive function in the elderly people. Frontiers in public health. 2022;10:1013734.
31. Chobe S, Chobe M, Metri K, Patra SK, Nagaratna R. Impact of Yoga on cognition and mental health among elderly: A systematic review. Complement Ther Med. 2020;52:102421.
32. Clouston SA, Brewster P, Kuh D, Richards M, Cooper R, Hardy R, et al. The dynamic relationship between physical function and cognition in longitudinal aging cohorts. Epidemiol Rev. 2013;35(1):33-50.
33. Coelho-Júnior HJ, Trichopoulou A, Panza F. Cross-sectional and longitudinal associations between adherence to Mediterranean diet with physical performance and cognitive function in older adults: A systematic review and meta-analysis. Ageing research reviews. 2021;70:101395.
34. Colcombe S, Kramer AF. Fitness effects on the cognitive function of older adults: a meta-analytic study. Psychol Sci. 2003;14(2):125-30.
35. Conti A, Voelkl J, McGuire F. The potential role of leisure in the prevention of dementia. Annual in Therapeutic Recreation. 2009;17:31-45.
36. Cooper R, Kuh D, Cooper C, Gale CR, Lawlor DA, Matthews F, et al. Objective measures of physical capability and subsequent health: a systematic review. Age and ageing. 2011;40(1):14-23.
37. Cordes T, Schoene D, Kemmler W, Wollesen B. Chair-Based Exercise Interventions for Nursing Home Residents: A Systematic Review. J Am Med Dir Assoc. 2021;22(4):733-40.
38. Cunningham C, R OS, Caserotti P, Tully MA. Consequences of physical inactivity in older adults: A systematic review of reviews and meta-analyses. Scand J Med Sci Sports. 2020;30(5):816-27.
39. Cunningham J, Broglio S, Wilson F. Influence of playing rugby on long-term brain health following retirement: a systematic review and narrative synthesis. BMJ open sport & exercise medicine. 2018;4(1):e000356.
40. Cunningham J, Broglio SP, O'Grady M, Wilson F. History of Sport-Related Concussion and Long-Term Clinical Cognitive Health Outcomes in Retired Athletes: A Systematic Review. Journal of athletic training. 2020;55(2):132-58.
41. Daviglus ML, Plassman BL, Pirzada A, Bell CC, Bowen PE, Burke JR, et al. Risk factors and preventive interventions for Alzheimer disease: state of the science. Arch Neurol. 2011;68(9):1185-90.
42. de Assis GG, de Almondes KM. Exercise-dependent BDNF as a Modulatory Factor for the Executive Processing of Individuals in Course of Cognitive Decline. A Systematic Review. Front Psychol. 2017;8:584.
43. de Keijzer C, Bauwelinck M, Dadvand P. Long-Term Exposure to Residential Greenspace and Healthy Ageing: a Systematic Review. Current environmental health reports. 2020;7(1):65-88.
44. De Silva NA, Gregory MA, Venkateshan SS, Verschoor CP, Kuspinar A. Examining the Association between Life-Space Mobility and Cognitive Function in Older Adults: A Systematic Review. Journal of aging research. 2019;2019:3923574.
45. Fernandes M. de Sousa A, Medeiros AR, Del Rosso S, Stults-Kolehmainen M, Boullosa DA. The influence of exercise and physical fitness status on attention: a systematic review. International Review of Sport and Exercise Psychology. 2019;12(1):202-34.
46. Dedeyne L, Deschodt M, Verschueren S, Tournoy J, Gielen E. Effects of multi-domain interventions in (pre)frail elderly on frailty, functional, and cognitive status: a systematic review. Clin Interv Aging. 2017;12:873-96.
47. Erlenbach E, McAuley E, Gothe NP. The Association Between Light Physical Activity and Cognition Among Adults: A Scoping Review. J Gerontol A Biol Sci Med Sci. 2021;76(4):716-24.

48. Etnier JL, Nowell PM, Landers DM, Sibley BA. A meta-regression to examine the relationship between aerobic fitness and cognitive performance. Brain research reviews. 2006;52(1):119-30.
49. Gallardo-Gómez D, Del Pozo-Cruz J, Noetel M, Álvarez-Barbosa F, Alfonso-Rosa RM, Del Pozo Cruz B. Optimal dose and type of exercise to improve cognitive function in older adults: A systematic review and bayesian model-based network meta-analysis of RCTs. Ageing research reviews. 2022;76:101591.
50. Gallou-Guyot M, Mandigout S, Bherer L, Perrochon A. Effects of exergames and cognitive-motor dual-task training on cognitive, physical and dual-task functions in cognitively healthy older adults: An overview. Ageing research reviews. 2020;63:101135.
51. Gavelin HM, Dong C, Minkov R, Bahar-Fuchs A, Ellis KA, Lautenschlager NT, et al. Combined physical and cognitive training for older adults with and without cognitive impairment: A systematic review and network meta-analysis of randomized controlled trials. Ageing research reviews. 2021;66:101232.
52. Gheysen F, Poppe L, DeSmet A, Swinnen S, Cardon G, De Bourdeaudhuij I, et al. Physical activity to improve cognition in older adults: can physical activity programs enriched with cognitive challenges enhance the effects? A systematic review and meta-analysis. Int J Behav Nutr Phys Act. 2018;15(1):63.
53. Gomes-Osman J, Cabral DF, Morris TP, McInerney K, Cahalin LP, Rundek T, et al. Exercise for cognitive brain health in aging: A systematic review for an evaluation of dose. Neurology Clinical practice. 2018;8(3):257-65.
54. Gothe NP, McAuley E. Yoga and Cognition: A Meta-Analysis of Chronic and Acute Effects. Psychosom Med. 2015;77(7):784-97.
55. Griebler N, Schröder N, Artifon M, Frigotto M, Pietta-Dias C. The effects of acute exercise on memory of cognitively healthy seniors: A systematic review. Arch Gerontol Geriatr. 2022;99:104583.
56. Gu Q, Zou L, Loprinzi PD, Quan M, Huang T. Effects of Open Versus Closed Skill Exercise on Cognitive Function: A Systematic Review. Front Psychol. 2019;10:1707.
57. Guimarães AV, Rocha SV, Barbosa AR. Exercise and co cise and co cise and cognitive performance in older adults: in older adults: a systema a systema a systematic review. Medicina (Ribeirão Preto). 2014;47(4):377-86.
58. Guo W, Zang M, Klich S, Kawczyński A, Smoter M, Wang B. Effect of Combined Physical and Cognitive Interventions on Executive Functions in OLDER Adults: A Meta-Analysis of Outcomes. Int J Environ Res Public Health. 2020;17(17).
59. Hajar MS, Rizal H, Kuan G. Effects of physical activity on sustained attention: A systematic review. Scientia Medica. 2019;29(2):e32864-e.
60. Han K, Tang Z, Bai Z, Su W, Zhang H. Effects of combined cognitive and physical intervention on enhancing cognition in older adults with and without mild cognitive impairment: A systematic review and meta-analysis. Front Aging Neurosci. 2022;14:878025.
61. Hatchard T, Ting JJ, Messier C. Translating the impact of exercise on cognition: methodological issues in animal research. Behav Brain Res. 2014;273:177-88.
62. Herold F, Törpel A, Schega L, Müller NG. Functional and/or structural brain changes in response to resistance exercises and resistance training lead to cognitive improvements - a systematic review. European review of aging and physical activity : official journal of the European Group for Research into Elderly and Physical Activity. 2019;16:10.
63. Herold F, Theobald P, Gronwald T, Kaushal N, Zou L, de Bruin ED, et al. Alexa, let's train now! - A systematic review and classification approach to digital and home-based physical training interventions aiming to support healthy cognitive aging. Journal of sport and health science. 2023.
64. Hewston P, Kennedy CC, Borhan S, Merom D, Santaguida P, Ioannidis G, et al. Effects of dance on cognitive function in older adults: a systematic review and meta-analysis. Age and ageing. 2021;50(4):1084-92.
65. Hindin S, Zelinski E, editors. Training for transfer: a meta-analysis of cognitive and physical fitness training in older adults. Gerontologist; 2009: Gerontological Soc Amer 1030 15th St Nw, ste 250, Washinton, DC 20005202

66. Hoffmann CM, Petrov ME, Lee RE. Aerobic physical activity to improve memory and executive function in sedentary adults without cognitive impairment: A systematic review and meta-analysis. Preventive medicine reports. 2021;23:101496.
67. Howes SC, Charles DK, Marley J, Pedlow K, McDonough SM. Gaming for Health: Systematic Review and Meta-analysis of the Physical and Cognitive Effects of Active Computer Gaming in Older Adults. Phys Ther. 2017;97(12):1122-37.
68. Hoy S, Östh J, Pascoe M, Kandola A, Hallgren M. Effects of yoga-based interventions on cognitive function in healthy older adults: A systematic review of randomized controlled trials. Complement Ther Med. 2021;58:102690.
69. Hsieh SS, Chueh TY, Huang CJ, Kao SC, Hillman CH, Chang YK, et al. Systematic review of the acute and chronic effects of high-intensity interval training on executive function across the lifespan. J Sports Sci. 2021;39(1):10-22.
70. Intzandt B, Vrinceanu T, Huck J, Vincent T, Montero-Odasso M, Gauthier CJ, et al. Comparing the effect of cognitive vs. exercise training on brain MRI outcomes in healthy older adults: A systematic review. Neurosci Biobehav Rev. 2021;128:511-33.
71. Jedrziewski MK, Lee VM, Trojanowski JQ. Physical activity and cognitive health. Alzheimer's & dementia : the journal of the Alzheimer's Association. 2007;3(2):98-108.
72. Ji L, Steffens DC, Wang L. Effects of physical exercise on the aging brain across imaging modalities: A meta-analysis of neuroimaging studies in randomized controlled trials. Int J Geriatr Psychiatry. 2021;36(8):1148-57.
73. Joubert C, Chainay H. Aging brain: the effect of combined cognitive and physical training on cognition as compared to cognitive and physical training alone - a systematic review. Clin Interv Aging. 2018;13:1267-301.
74. Jung M, Zou L, Yu JJ, Ryu S, Kong Z, Yang L, et al. Does exercise have a protective effect on cognitive function under hypoxia? A systematic review with meta-analysis. Journal of sport and health science. 2020;9(6):562-77.
75. Karr JE, Areshenkoff CN, Rast P, Garcia-Barrera MA. An empirical comparison of the therapeutic benefits of physical exercise and cognitive training on the executive functions of older adults: a meta-analysis of controlled trials. Neuropsychology. 2014;28(6):829-45.
76. Kelly ME, Loughrey D, Lawlor BA, Robertson IH, Walsh C, Brennan S. The impact of exercise on the cognitive functioning of healthy older adults: a systematic review and meta-analysis. Ageing research reviews. 2014;16:12-31.
77. Koch SC, Riege RFF, Tisborn K, Biondo J, Martin L, Beelmann A. Effects of Dance Movement Therapy and Dance on Health-Related Psychological Outcomes. A Meta-Analysis Update. Front Psychol. 2019;10:1806.
78. Kramer AF, Colcombe SJ, McAuley E, Eriksen KI, Scalf P, Jerome GJ, et al. Enhancing brain and cognitive function of older adults through fitness training. J Mol Neurosci. 2003;20(3):213-21.
79. Kramer AF, Colcombe S. Fitness Effects on the Cognitive Function of Older Adults: A Meta-Analytic Study-Revisited. Perspect Psychol Sci. 2018;13(2):213-7.
80. Kramer AF, Erickson KI. Effects of physical activity on cognition, well-being, and brain: human interventions. Alzheimer's & dementia : the journal of the Alzheimer's Association. 2007;3(2 Suppl):S45-51.
81. Laird KT, Paholpak P, Roman M, Rahi B, Lavretsky H. Mind-Body Therapies for Late-Life Mental and Cognitive Health. Current psychiatry reports. 2018;20(1):2.
82. Landrigan JF, Bell T, Crowe M, Clay OJ, Mirman D. Lifting cognition: a meta-analysis of effects of resistance exercise on cognition. Psychol Res. 2020;84(5):1167-83.
83. Lauenroth A, Ioannidis AE, Teichmann B. Influence of combined physical and cognitive training on cognition: a systematic review. BMC Geriatr. 2016;16:141.
84. Law LL, Barnett F, Yau MK, Gray MA. Effects of combined cognitive and exercise interventions on cognition in older adults with and without cognitive impairment: a systematic review. Ageing research reviews. 2014;15:61-75.
85. Lee Y, Back JH, Kim J, Kim SH, Na DL, Cheong HK, et al. Systematic review of health behavioral risks and cognitive health in older adults. Int Psychogeriatr. 2010;22(2):174-87.

86. Lehert P, Villaseca P, Hogervorst E, Maki PM, Henderson VW. Individually modifiable risk factors to ameliorate cognitive aging: a systematic review and meta-analysis. Climacteric. 2015;18(5):678-89.
87. Lei X, Wu Y, Xu M, Jones OD, Ma J, Xu X. Physical exercise: bulking up neurogenesis in human adults. Cell & bioscience. 2019;9:74.
88. Leung G, Lam L. Leisure Activities and Cognitive Impairment in Late Life--a Selective Literature Review of Longitudinal Cohort Studies. Hong Kong Journal of Psychiatry. 2007;17(3).
89. Levin O, Netz Y, Ziv G. The beneficial effects of different types of exercise interventions on motor and cognitive functions in older age: a systematic review. European review of aging and physical activity : official journal of the European Group for Research into Elderly and Physical Activity. 2017;14:20.
90. Li Z, Peng X, Xiang W, Han J, Li K. The effect of resistance training on cognitive function in the older adults: a systematic review of randomized clinical trials. Aging Clin Exp Res. 2018;30(11):1259-73.
91. Li F, Wang L, Qin Y, Liu G. Combined Tai Chi and cognitive interventions for older adults with or without cognitive impairment: A meta-analysis and systematic review. Complement Ther Med. 2022;67:102833.
92. Li Q, Gong B, Zhao Y, Wu C. Effect of Exercise Cognitive Combined Training on Physical Function in Cognitively Healthy Older Adults: A Systematic Review and Meta-Analysis. Journal of aging and physical activity. 2023;31(1):155-70.
93. Lipnicki DM, Makkar SR, Crawford JD, Thalamuthu A, Kochan NA, Lima-Costa MF, et al. Determinants of cognitive performance and decline in 20 diverse ethno-regional groups: A COSMIC collaboration cohort study. PLoS Med. 2019;16(7):e1002853.
94. Liu F, Chen X, Nie P, Lin S, Guo J, Chen J, et al. Can Tai Chi Improve Cognitive Function? A Systematic Review and Meta-Analysis of Randomized Controlled Trials. J Altern Complement Med. 2021;27(12):1070-83.
95. Liu T, Li N, Hou Z, Liu L, Gao L, Wang L, et al. Nutrition and exercise interventions could ameliorate age-related cognitive decline: a meta-analysis of randomized controlled trials. Aging Clin Exp Res. 2021;33(7):1799-809.
96. Loprinzi PD, Frith E, Edwards MK. Resistance exercise and episodic memory function: a systematic review. Clin Physiol Funct Imaging. 2018.
97. Ludyga S, Gerber M, Brand S, Holsboer-Trachsler E, Pühse U. Acute effects of moderate aerobic exercise on specific aspects of executive function in different age and fitness groups: A meta-analysis. Psychophysiology. 2016;53(11):1611-26.
98. Ludyga S, Gerber M, Pühse U, Looser VN, Kamijo K. Systematic review and meta-analysis investigating moderators of long-term effects of exercise on cognition in healthy individuals. Nature human behaviour. 2020;4(6):603-12.
99. Luu K, Hall PA. Hatha Yoga and Executive Function: A Systematic Review. J Altern Complement Med. 2016;22(2):125-33.
100. Lü J, Fu W, Liu Y. Physical activity and cognitive function among older adults in China: A systematic review. Journal of sport and health science. 2016;5(3):287-96.
101. Mansor NS, Chow CM, Halaki M. Cognitive effects of video games in older adults and their moderators: a systematic review with meta-analysis and meta-regression. Aging & mental health. 2020;24(6):841-56.
102. McMorris T, Hale BJ. Differential effects of differing intensities of acute exercise on speed and accuracy of cognition: a meta-analytical investigation. Brain Cogn. 2012;80(3):338-51.
103. McSween MP, Coombes JS, MacKay CP, Rodriguez AD, Erickson KI, Copland DA, et al. The Immediate Effects of Acute Aerobic Exercise on Cognition in Healthy Older Adults: A Systematic Review. Sports Med. 2019;49(1):67-82.
104. Meng X, Li G, Jia Y, Liu Y, Shang B, Liu P, et al. Effects of dance intervention on global cognition, executive function and memory of older adults: a meta-analysis and systematic review. Aging Clin Exp Res. 2020;32(1):7-19.

105. Menichetti J, Cipresso P, Bussolin D, Graffigna G. Engaging older people in healthy and active lifestyles: a systematic review. Ageing & Society. 2016;36(10):2036-60.
106. Miller KJ, Adair BS, Pearce AJ, Said CM, Ozanne E, Morris MM. Effectiveness and feasibility of virtual reality and gaming system use at home by older adults for enabling physical activity to improve health-related domains: a systematic review. Age and ageing. 2014;43(2):188-95.
107. Miller SM, Taylor-Piliae RE. Effects of Tai Chi on cognitive function in community-dwelling older adults: a review. Geriatr Nurs. 2014;35(1):9-19.
108. Miller SM, Taylor-Piliae RE, Insel KC. The association of physical activity, cognitive processes and automobile driving ability in older adults: A review of the literature. Geriatr Nurs. 2016;37(4):313-20.
109. Morat M, Morat T, Zijlstra W, Donath L. Effects of multimodal agility-like exercise training compared to inactive controls and alternative training on physical performance in older adults: a systematic review and meta-analysis. European review of aging and physical activity : official journal of the European Group for Research into Elderly and Physical Activity. 2021;18(1):4.
110. Moreau D, Chou E. The Acute Effect of High-Intensity Exercise on Executive Function: A Meta-Analysis. Perspect Psychol Sci. 2019;14(5):734-64.
111. Muiños M, Ballesteros S. Does dance counteract age-related cognitive and brain declines in middle-aged and older adults? A systematic review. Neurosci Biobehav Rev. 2021;121:259-76.
112. Northey JM, Cherbuin N, Pumpa KL, Smee DJ, Rattray B. Exercise interventions for cognitive function in adults older than 50: a systematic review with meta-analysis. Br J Sports Med. 2018;52(3):154-60.
113. Oberste M, Javelle F, Sharma S, Joisten N, Walzik D, Bloch W, et al. Effects and Moderators of Acute Aerobic Exercise on Subsequent Interference Control: A Systematic Review and Meta-Analysis. Front Psychol. 2019;10:2616.
114. Oberste M, Sharma S, Bloch W, Zimmer P. Acute Exercise-Induced Set Shifting Benefits in Healthy Adults and Its Moderators: A Systematic Review and Meta-Analysis. Front Psychol. 2021;12:528352.
115. Ogawa EF, You T, Leveille SG. Potential Benefits of Exergaming for Cognition and Dual-Task Function in Older Adults: A Systematic Review. Journal of aging and physical activity. 2016;24(2):332-6.
116. Olanrewaju O, Kelly S, Cowan A, Brayne C, Lafortune L. Physical Activity in Community Dwelling Older People: A Systematic Review of Reviews of Interventions and Context. PLoS One. 2016;11(12):e0168614.
117. Olanrewaju O, Stockwell S, Stubbs B, Smith L. Sedentary behaviours, cognitive function, and possible mechanisms in older adults: a systematic review. Aging Clin Exp Res. 2020;32(6):969-84.
118. Origua Rios S, Marks J, Estevan I, Barnett LM. Health benefits of hard martial arts in adults: a systematic review. J Sports Sci. 2018;36(14):1614-22.
119. Patel NK, Newstead AH, Ferrer RL. The effects of yoga on physical functioning and health related quality of life in older adults: a systematic review and meta-analysis. J Altern Complement Med. 2012;18(10):902-17.
120. Paterson DH, Warburton DE. Physical activity and functional limitations in older adults: a systematic review related to Canada's Physical Activity Guidelines. Int J Behav Nutr Phys Act. 2010;7:38.
121. Pessoa RF, Neves CM, Ferreira MEC. Dance therapy in aging: A systematic review. Journal of Physical Education and Sport. 2019;19(2):1180-7.
122. Predovan D, Julien A, Esmail A, Bherer L. Effects of Dancing on Cognition in Healthy Older Adults: a Systematic Review. Journal of cognitive enhancement : towards the integration of theory and practice. 2019;3(2):161-7.
123. Ramsey KA, Meskers CGM, Maier AB. Every step counts: synthesising reviews associating objectively measured physical activity and sedentary behaviour with clinical

outcomes in community-dwelling older adults. The Lancet Healthy longevity. 2021;2(11):e764-e72.
124. Rathore A, Lom B. The effects of chronic and acute physical activity on working memory performance in healthy participants: a systematic review with meta-analysis of randomized controlled trials. Systematic reviews. 2017;6(1):124.
125. Ren FF, Chen FT, Zhou WS, Cho YM, Ho TJ, Hung TM, et al. Effects of Chinese Mind-Body Exercises on Executive Function in Middle-Aged and Older Adults: A Systematic Review and Meta-Analysis. Front Psychol. 2021;12:656141.
126. Roberts CE, Phillips LH, Cooper CL, Gray S, Allan JL. Effect of Different Types of Physical Activity on Activities of Daily Living in Older Adults: Systematic Review and Meta-Analysis. Journal of aging and physical activity. 2017;25(4):653-70.
127. Rojer AGM, Ramsey KA, Amaral Gomes ES, D'Andrea L, Chen C, Szoeke C, et al. Objectively assessed physical activity and sedentary behavior and global cognitive function in older adults: a systematic review. Mech Ageing Dev. 2021;198:111524.
128. Russo M, Kañevsky A, Leis A, Iturry M, Roncoroni M, Serrano C. Role of physical activity in preventing cognitive impairment and dementia in older adults: A systematic review. Neurol Argentina. 2020;12(2):124-37.
129. Sáez de Asteasu ML, Martínez-Velilla N, Zambom-Ferraresi F, Casas-Herrero Á, Izquierdo M. Role of physical exercise on cognitive function in healthy older adults: A systematic review of randomized clinical trials. Ageing research reviews. 2017;37:117-34.
130. Sakaki K, Nouchi R, Matsuzaki Y, Saito T, Dinet J, Kawashima R. Benefits of VR Physical Exercise on Cognition in Older Adults with and without Mild Cognitive Decline: A Systematic Review of Randomized Controlled Trials. Healthcare (Basel, Switzerland). 2021;9(7).
131. Sanders LMJ, Hortobágyi T, la Bastide-van Gemert S, van der Zee EA, van Heuvelen MJG. Dose-response relationship between exercise and cognitive function in older adults with and without cognitive impairment: A systematic review and meta-analysis. PLoS One. 2019;14(1):e0210036.
132. Sexton BP, Taylor NF. To sit or not to sit? A systematic review and meta-analysis of seated exercise for older adults. Australas J Ageing. 2019;38(1):15-27.
133. Silva RB, Aldoradin-Cabeza H, Eslick GD, Phu S, Duque G. The Effect of Physical Exercise on Frail Older Persons: A Systematic Review. The Journal of frailty & aging. 2017;6(2):91-6.
134. Smith PJ, Blumenthal JA, Hoffman BM, Cooper H, Strauman TA, Welsh-Bohmer K, et al. Aerobic exercise and neurocognitive performance: a meta-analytic review of randomized controlled trials. Psychosom Med. 2010;72(3):239-52.
135. Snowden M, Steinman L, Mochan K, Grodstein F, Prohaska TR, Thurman DJ, et al. Effect of exercise on cognitive performance in community-dwelling older adults: review of intervention trials and recommendations for public health practice and research. J Am Geriatr Soc. 2011;59(4):704-16.
136. Soares VN, Yoshida HM, Magna TS, Sampaio RAC, Fernandes PT. Comparison of exergames versus conventional exercises on the cognitive skills of older adults: a systematic review with meta-analysis. Arch Gerontol Geriatr. 2021;97:104485.
137. Sofi F, Valecchi D, Bacci D, Abbate R, Gensini GF, Casini A, et al. Physical activity and risk of cognitive decline: a meta-analysis of prospective studies. J Intern Med. 2011;269(1):107-17.
138. Solloway MR, Taylor SL, Shekelle PG, Miake-Lye IM, Beroes JM, Shanman RM, et al. An evidence map of the effect of Tai Chi on health outcomes. Systematic reviews. 2016;5(1):126.
139. Stanmore E, Stubbs B, Vancampfort D, de Bruin ED, Firth J. The effect of active video games on cognitive functioning in clinical and non-clinical populations: A meta-analysis of randomized controlled trials. Neurosci Biobehav Rev. 2017;78:34-43.
140. Stein AM, Silva TMV, Coelho FGM, Arantes FJ, Costa JLR, Teodoro E, et al. Physical exercise, IGF-1 and cognition A systematic review of experimental studies in the elderly. Dementia & neuropsychologia. 2018;12(2):114-22.

141. Stojan R, Voelcker-Rehage C. A Systematic Review on the Cognitive Benefits and Neurophysiological Correlates of Exergaming in Healthy Older Adults. Journal of clinical medicine. 2019;8(5).
142. Teixeira-Machado L, Arida RM, de Jesus Mari J. Dance for neuroplasticity: A descriptive systematic review. Neurosci Biobehav Rev. 2019;96:232-40.
143. Tseng CN, Gau BS, Lou MF. The effectiveness of exercise on improving cognitive function in older people: a systematic review. The journal of nursing research : JNR. 2011;19(2):119-31.
144. Turner DT, Hu MX, Generaal E, Bos D, Ikram MK, Heshmatollah A, et al. Physical Exercise Interventions Targeting Cognitive Functioning and the Cognitive Domains in Nondementia Samples: A Systematic Review of Meta-Analyses. J Geriatr Psychiatry Neurol. 2021;34(2):91-101.
145. van Uffelen JG, Chin APMJ, Hopman-Rock M, van Mechelen W. The effects of exercise on cognition in older adults with and without cognitive decline: a systematic review. Clin J Sport Med. 2008;18(6):486-500.
146. Vaportzis E, Niechcial MA, Gow AJ. A systematic literature review and meta-analysis of real-world interventions for cognitive ageing in healthy older adults. Ageing research reviews. 2019;50:110-30.
147. Wang P, Liu HH, Zhu XT, Meng T, Li HJ, Zuo XN. Action Video Game Training for Healthy Adults: A Meta-Analytic Study. Front Psychol. 2016;7:907.
148. Wang X, Wu J, Ye M, Wang L, Zheng G. Effect of Baduanjin exercise on the cognitive function of middle-aged and older adults: A systematic review and meta-analysis. Complement Ther Med. 2021;59:102727.
149. Wayne PM, Walsh JN, Taylor-Piliae RE, Wells RE, Papp KV, Donovan NJ, et al. Effect of tai chi on cognitive performance in older adults: systematic review and meta-analysis. J Am Geriatr Soc. 2014;62(1):25-39.
150. Whitty E, Mansour H, Aguirre E, Palomo M, Charlesworth G, Ramjee S, et al. Efficacy of lifestyle and psychosocial interventions in reducing cognitive decline in older people: Systematic review. Ageing research reviews. 2020;62:101113.
151. Wilke J, Giesche F, Klier K, Vogt L, Herrmann E, Banzer W. Acute Effects of Resistance Exercise on Cognitive Function in Healthy Adults: A Systematic Review with Multilevel Meta-Analysis. Sports Med. 2019;49(6):905-16.
152. Williams KN, Kemper S. Interventions to reduce cognitive decline in aging. J Psychosoc Nurs Ment Health Serv. 2010;48(5):42-51.
153. Wollesen B, Wildbredt A, van Schooten KS, Lim ML, Delbaere K. The effects of cognitive-motor training interventions on executive functions in older people: a systematic review and meta-analysis. European review of aging and physical activity : official journal of the European Group for Research into Elderly and Physical Activity. 2020;17:9.
154. Wu C, Yi Q, Zheng X, Cui S, Chen B, Lu L, et al. Effects of Mind-Body Exercises on Cognitive Function in Older Adults: A Meta-Analysis. J Am Geriatr Soc. 2019;67(4):749-58.
155. Wu J, Wang X, Ye M, Wang L, Zheng G. Effect of regular resistance training on memory in older adults: A systematic review. Exp Gerontol. 2021;150:111396.
156. Wu Y, Wang Y, Burgess EO, Wu J. The effects of Tai Chi exercise on cognitive function in older adults: A meta-analysis. Journal of sport and health science. 2013;2(4):193-203.
157. Xu W, Liang HN, Baghaei N, Wu Berberich B, Yue Y. Health Benefits of Digital Videogames for the Aging Population: A Systematic Review. Games for health journal. 2020;9(6):389-404.
158. Xu L, Gu H, Cai X, Zhang Y, Hou X, Yu J, et al. The Effects of Exercise for Cognitive Function in Older Adults: A Systematic Review and Meta-Analysis of Randomized Controlled Trials. Int J Environ Res Public Health. 2023;20(2).
159. Yang FC, Desai AB, Esfahani P, Sokolovskaya TV, Bartlett DJ. Effectiveness of Tai Chi for Health Promotion of Older Adults: A Scoping Review of Meta-Analyses. Am J Lifestyle Med. 2022;16(6):700-16.

160. Yang C, Han X, Jin M, Xu J, Wang Y, Zhang Y, et al. The Effect of Video Game-Based Interventions on Performance and Cognitive Function in Older Adults: Bayesian Network Meta-analysis. JMIR serious games. 2021;9(4):e27058.
161. Yen HY, Chiu HL. Virtual Reality Exergames for Improving Older Adults' Cognition and Depression: A Systematic Review and Meta-Analysis of Randomized Control Trials. J Am Med Dir Assoc. 2021;22(5):995-1002.
162. Yoneda T, Lewis NA, Knight JE, Rush J, Vendittelli R, Kleineidam L, et al. The Importance of Engaging in Physical Activity in Older Adulthood for Transitions Between Cognitive Status Categories and Death: A Coordinated Analysis of 14 Longitudinal Studies. J Gerontol A Biol Sci Med Sci. 2021;76(9):1661-7.
163. Young J, Angevaren M, Rusted J, Tabet N. Aerobic exercise to improve cognitive function in older people without known cognitive impairment. The Cochrane database of systematic reviews. 2015(4):Cd005381.
164. Zhang F, Kaufman D. Physical and Cognitive Impacts of Digital Games on Older Adults: A Meta-Analytic Review. J Appl Gerontol. 2016;35(11):1189-210.
165. Zheng G, Liu F, Li S, Huang M, Tao J, Chen L. Tai Chi and the Protection of Cognitive Ability: A Systematic Review of Prospective Studies in Healthy Adults. Am J Prev Med. 2015;49(1):89-97.
166. Zheng K, Zou L, Wei G, Huang T. Concurrent Performance of Executive Function during Acute Bouts of Exercise in Adults: A Systematic Review. Brain sciences. 2021;11(10).
167. Zheng J, Su X, Xu C. Effects of exercise intervention on executive function of middle-aged and elderly people: A systematic review of randomized controlled trials. Front Aging Neurosci. 2022;14:960817.
168. Zhidong C, Wang X, Yin J, Song D, Chen Z. Effects of physical exercise on working memory in older adults: a systematic and meta-analytic review. European review of aging and physical activity : official journal of the European Group for Research into Elderly and Physical Activity. 2021;18(1):18.
169. Zhu H, Chen A, Guo W, Zhu F, Wang B. Which type of exercise is more beneficial for cognitive function? A meta-analysis of the effects of open-skill exercise versus closed-skill exercise among children, adults, and elderly populations. Applied Sciences. 2020;10(8):2737.
170. Zhu X, Yin S, Lang M, He R, Li J. The more the better? A meta-analysis on effects of combined cognitive and physical intervention on cognition in healthy older adults. Ageing research reviews. 2016;31:67-79.
171. Zou L, Yu Q, Liu S, Loprinzi PD. Exercise on Visuo-Spatial Memory: Direct Effects and Underlying Mechanisms. Am J Health Behav. 2020;44(2):169-79.
172. Zou L, Huang T, Tsang T, Pan Z, Wang C, Liu Y, et al. Hard martial arts for cognitive function across the lifespan: A systematic review. Archives of Budo. 2018;14:41-58.
173. Zülke AE, Riedel-Heller SG, Wittmann F, Pabst A, Röhr S, Luppa M. Gender-Specific Design and Effectiveness of Non-Pharmacological Interventions against Cognitive Decline - Systematic Review and Meta-Analysis of Randomized Controlled Trials. The journal of prevention of Alzheimer's disease. 2023;10(1):69-82.
