# Supplementary material for: Mind body exercise improves cognitive function more than aerobic- and resistance exercise in healthy adults aged 55 years and older – an umbrella review
Source: Eur Rev Aging Phys Act. 2023 Aug 9;20:15. doi: 10.1186/s11556-023-00325-4 (PMC10413530; doi:10.1186/s11556-023-00325-4)
Supplement: Supplementary file 3 — Additional file 3: Supplement S3. Included studies overlap. [file 11556_2023_325_MOESM3_ESM.pdf]

Supplement S3. Included studies overlap

Author	Year	Angevaren et al (7) 2008	Bhattacharyya et al (9) 2020	Chen et al (10) 2020	Clifford et al (48) 2023	Coelho-Junior et al (8) 2022	Falck et al (40) 2019	Gasquoine and Chen (2022) 2022	Hindin and Zelinski (51) 2012	Jiang et al (49) 2022	Loprinzi et al (45) 2019	Ma et al (13) 2023	Martins et al (50) 2022	Roig et al (14) 2013	Scherder et al (52) 2014	Wang et al (46) 2021	Xiong et al (39) 2020	Ye et al (32) 2021	Zhao et al (53) 2022	Zhidong et al (55) 2021	Zhu et al (54) 2023
Ackerman	2010								•												
Adcock	2020									•											
Adamczyk	2020											•									
Aguiñaga & Marqu	2019				•																
Albinet	2016			•			•													•	
Albinet	2010			•													•				
Alves	2013							•				•									
Anderson-Hanley	2012									•											
Ansai, Rebelatto	2015					•	•	•											•		
Antunes, De Mello	2015						•	•									•				
Antunes, Santos-G	2015						•	•									•				
Areeudomwong	2019				•																
Audiffren	2009													•							
Bae	2019																			•	
Bakken	2001	•												•							
Baniqued	2017																•				
Bantoft	2016										•										
Barnes	2013						•	•													
Barry	1966								•												
Basak	2008								•												
Basso	2015										•										
Bean	2004												•								
Beijersbergen	2017												•								
Beijersbergen	2017												•								
Beijersbergen	2017												•								
Bennett & Hackney	2018				•							•									
Berryman	2014						•										•				
Bherer	2005								•												
Bherer	2008								•												
Biasutti	2018											•									
Blumenthal	1989	•					•		•					•							
Boa Sorte Silva	2017															•					
Boa Sorte Silva	2018															•					
Boa Sorte Silva	2020															•					
Bolandzadeh	2015						•														
Borella	2010								•												
Borges	2012											•									
Bouaziz	2019																		•		
Brown, Liu-Ambros	2009						•	•												•	•

Author	Year	2008 Angevaren et al (7)	2020 Bhattacharyya et al (9)	2020 Chen et al (10)	2023 Clifford et al (48)	2022 Coelho-Junior et al (8)	2019 Falck et al (40)	2022 Gasquoine and Chen (2022)	2012 Hindin and Zelinski (51)	2022 Jiang et al (49)	2019 Loprinzi et al (45)	2023 Ma et al (13)	2022 Martins et al (50)	2013 Roig et al (14)	2014 Scherder et al (52)	2021 Wang et al (46)	2020 Xiong et al (39)	2021 Ye et al (32)	2022 Zhao et al (53)	2021 Zhidong et al (55)	2023 Zhu et al (54)
Buschkuehl	2008								•												
Cai	2018																	•			
Cassilhas	2007					•	•	•									•				
Cepeda	2015				•							•									
Chang	2017															•					
Chapman	2013																•				
Cherup	2018																•				
Clark	1987								•												
Coelho-Junior	2020					•															
Coetsee & Terblan	2017			•																	
Coles	2008													•							
Covassin	2007													•							
Crush	2017										•										
Cruz-Ferreira	2015				•							•									
da Silva	2010								•												
Dahlin	2008								•												
Damirichi	2018																			•	
Davey	1973													•							
Degé	2018											•									
Delancey	2018										•										
Desjardins-Crepea	2016						•														
Dietrich	2004													•							
Dillon	2020																		•		
Doi	2017			•																	
Donnezan	2018																			•	
Drew	1986								•												
Dustman	1984													•	•						
Edwards	2002								•												
Eggenberger	2016						•			•										•	
Eggenberger	2015											•									
Eggermont	2009																			•	
Elkana	2018																				•
Emery	1998	•																			
Emery	1990	•							•												
Emery	md													•							
Emery, Gatz	1990							•													
Erickson	2011			•																	
Erickson	md													•							
Esmail	2020											•									

Author	Year	2008 Angevaren et al (7)	2020 Bhattacharyya et al (9)	2020 Chen et al (10)	2023 Clifford et al (48)	2022 Coelho-Junior et al (8)	2019 Falck et al (40)	2022 Gasquoine and Chen (2022)	2012 Hindin and Zelinski (51)	2022 Jiang et al (49)	2019 Loprinzi et al (45)	2023 Ma et al (13)	2022 Martins et al (50)	2013 Roig et al (14)	2014 Scherder et al (52)	2021 Wang et al (46)	2020 Xiong et al (39)	2021 Ye et al (32)	2022 Zhao et al (53)	2021 Zhidong et al (55)	2023 Zhu et al (54)
Evers	2011																•				
Eyigor	2009											•									
Eyre	2016		•																		
Eyre	2017		•																		
Fabre	2002	•					•		•					•						•	•
Fang	2020												•								
Ferreira	2015			•				•												•	
Fragala	2014					•															
Franco	2020				•							•									
Frandin	2016						•														
Frith	2017										•										
Gallego	2011		•																		
Gao	2021											•									
Goldstein	1997								•												
Gothe	2014		•	•													•	•			
Gothe	2016			•																•	
Gothe	2017			•																	
Granacher	2012				•							•									
Griffin	2011													•							
Gschwind	2015						•			•											
Guimarães, Barbos	2018									•											
Hamacher	2016											•									
Hamacher	2015				•																
Hariprasad	2013		•															•		•	•
Hars	2014											•									
Hassmén	1992													•							
Hassmén	1997	•												•							
Hawkins	1992								•												
Haynes	2018										•										
Heath	2017															•					
Hill	1993								•												
Hill	md													•							
Hiyama	2012														•						
Hofgaard	2019				•																
Holmerová	2010											•									
Hong	2018					•														•	•
Hopkins	2012													•							
Hsu	2017						•														
Hu	2014						•														

Author	Year	2008 Angevaren et al (7)	2020 Bhattacharyya et al (9)	2020 Chen et al (10)	2023 Clifford et al (48)	2022 Coelho-Junior et al (8)	2019 Falck et al (40)	2022 Gasquoine and Chen (2022)	2012 Hindin and Zelinski (51)	2022 Jiang et al (49)	2019 Loprinzi et al (45)	2023 Ma et al (13)	2022 Martins et al (50)	2013 Roig et al (14)	2014 Scherder et al (52)	2021 Wang et al (46)	2020 Xiong et al (39)	2021 Ye et al (32)	2022 Zhao et al (53)	2021 Zhidong et al (55)	2023 Zhu et al (54)
Hvid	2016												•								
Hötting	2012													•							
Ijuin	2013							•													
Im	2019											•									
Innes	2016		•																		
Innes	2018		•																		
Iuliano	2015			•													•				
Janyacharoen	2013				•							•									
Janyacharoen	2018											•									
Jean	2010											•									
Jennings	2005								•												
Jonasson	2016																•				
Joung	2019				•																
Jun	2015											•									
Kalbe	2018																			•	•
Kamegaya	2014						•														
Karbach	2009								•												
Kattenstroth	2013						•					•									
Katula	2008												•								
Keyan	2017										•										
Kim	2021											•									
Kimura	2010			•																	
Klusmann	2010			•				•									•				
Kosmat	2017											•									
Kramer	1999								•												
Kramer	1995								•												
Kramer	1984														•						
Kramer	2001	•															•				
Kramer	2002																				•
Krampe	2013				•																
Kuo	2018						•														
Labban	2011										•			•							
Labban	2018										•										
Lachman	2006					•		•												•	
Lam	2015			•																	
Lam	2012																	•		•	
Lam	2010																				
Lambourne	2010													•							
Lambourne	2012													•							

Author	Year	2008 Angevaren et al (7)	2020 Bhattacharyya et al (9)	2020 Chen et al (10)	2023 Clifford et al (48)	2022 Coelho-Junior et al (8)	2019 Falck et al (40)	2022 Gasquoine and Chen (2022)	2012 Hindin and Zelinski (51)	2022 Jiang et al (49)	2019 Loprinzi et al (45)	2023 Ma et al (13)	2022 Martins et al (50)	2013 Roig et al (14)	2014 Scherder et al (52)	2021 Wang et al (46)	2020 Xiong et al (39)	2021 Ye et al (32)	2022 Zhao et al (53)	2021 Zhidong et al (55)	2023 Zhu et al (54)
Langlois	2013						•										•				
Lavretsky	2013		•																		
Lazarou	2017			•																	
Leckie	2014			•																	
Lee	2020												•								
Legault	2011																•				
Li	2016																			•	•
Li	2019																				•
Li	2020																				•
Liao	2019									•											
Liao	2020									•											
Lin	2016																	•			
Lin	2019																	•			
Linde	2014						•	•													
Lindheimer	2017																				•
Liu	2018																	•			
Liu-Ambrose	2012			•																	
Liu-Ambrose	2010			•		•	•	•												•	
Liu-Ambrose	2008						•	•									•				
Liu-Ambrose	2016						•														
Lo Bue-Estes	md													•							
Loprinzi	2015										•										
Lord	2003						•														
Lord	1994								•												
Lu	2016			•			•														
Lü	2015																			•	
Lustig	2008								•												
Machacova	2017											•									
Maclean	2014											•									
Madden	1989	•												•							
Mahncke	2006								•												
Maillot	2012													•							
Maki	2012							•													
Malliot	2012						•	•		•											
Marsh	2009												•								
Martins	2013													•							
Matson	2019																		•		
Matsufuji	2015						•														
Mavros	2017			•																	

Author	Year	2008 Angevaren et al (7)	2020 Bhattacharyya et al (9)	2020 Chen et al (10)	2023 Clifford et al (48)	2022 Coelho-Junior et al (8)	2019 Falck et al (40)	2022 Gasquoine and Chen (2022)	2012 Hindin and Zelinski (51)	2022 Jiang et al (49)	2019 Loprinzi et al (45)	2023 Ma et al (13)	2022 Martins et al (50)	2013 Roig et al (14)	2014 Scherder et al (52)	2021 Wang et al (46)	2020 Xiong et al (39)	2021 Ye et al (32)	2022 Zhao et al (53)	2021 Zhidong et al (55)	2023 Zhu et al (54)
McKinkley	2008											•									
McMorris	2008													•							
McNerney	2017										•										
Merom	2016				•		•					•									
Middleton	2018						•														
Miles	1998													•							
Miszko	2003												•								
Monteiro-Junior	2017									•											
Moreau	2017																				•
Mortimer	2012							•									•	•			
Most	2017										•										
Moul	1995	•					•		•												
Moul	md													•							
Moynihan	2013		•																		
Mozolic	2011								•												
Müller	2017				•																
Muscari	2009						•														
Nagamatsu	2013			•			•														
Nagamatsu	2012						•														
Nanda	2013										•										
Napoli	2014						•	•													
Nasimento	2014						•														
Nasimento	2015						•														
Netz	md													•							
Nguyen & Kruse	2012			•																	
Niemann	2014						•														
Nishiguchi	2015			•																•	•
Nocera	2015			•				•											•		
Nocera	2013																	•			
Noopud	2019				•							•									
Norouzi	2019																			•	
Nouchi	2014			•													•			•	•
Oken	1999														•						
Oken	2006		•	•				•										•			
Oken	md													•							
Okumiya	1996								•												
Ordnung	2017									•											
Pahor	2006						•														
Pahor	2014						•														

Author	Year	2008 Angevaren et al (7)	2020 Bhattacharyya et al (9)	2020 Chen et al (10)	2023 Clifford et al (48)	2022 Coelho-Junior et al (8)	2019 Falck et al (40)	2022 Gasquoine and Chen (2022)	2012 Hindin and Zelinski (51)	2022 Jiang et al (49)	2019 Loprinzi et al (45)	2023 Ma et al (13)	2022 Martins et al (50)	2013 Roig et al (14)	2014 Scherder et al (52)	2021 Wang et al (46)	2020 Xiong et al (39)	2021 Ye et al (32)	2022 Zhao et al (53)	2021 Zhidong et al (55)	2023 Zhu et al (54)
Pantoa	1990	•																			
Park	2017											•									
Peng & Zhou	2016																				•
Perri	1984								•												
Perri	md													•							
Perrig-Chiello	1998					•															
Petrosyan	2013					•															
Phirom, Kamnards	2020									•											
Pichierri	2012						•					•									
Pontifex	2009													•							
Potter	2005													•							
Prehn	2017			•																	
Rehfeld	2018				•																
Rehfeld	2017				•																
Reid	2008												•								
Rikli	1991								•												
Roche	2009								•												
Rodrigues-Rodrigu	2018				•																
Rogan	2016											•									
Roig	2012													•							
Ruschweyh	2011													•							
Salas	2011										•										
Sayers & Gibson	2010												•								
Schatil	2013																•				
Schattin	2016						•			•											
Scherder	2005																			•	
Schigematsu	2006															•					
Schigematsu	2008															•					
Schmidt-Kassow	2014										•										
Schoene	2013						•			•											
Schoene	2015						•			•											
Schramke	1997										•			•							
Segal	md													•							
Sejnoha Minsterov	2020				•																
Serrano-Guzmán	2016											•									
Shan	2016																			•	
Shigematsu	2002											•									
Shimizu	2013											•									
Sibley	2007													•							

Author	Year	2008 Angevaren et al (7)	2020 Bhattacharyya et al (9)	2020 Chen et al (10)	2023 Clifford et al (48)	2022 Coelho-Junior et al (8)	2019 Falck et al (40)	2022 Gasquoine and Chen (2022)	2012 Hindin and Zelinski (51)	2022 Jiang et al (49)	2019 Loprinzi et al (45)	2023 Ma et al (13)	2022 Martins et al (50)	2013 Roig et al (14)	2014 Scherder et al (52)	2021 Wang et al (46)	2020 Xiong et al (39)	2021 Ye et al (32)	2022 Zhao et al (53)	2021 Zhidong et al (55)	2023 Zhu et al (54)
Siddiqui	2018										•										
Sink	2015						•	•									•				
Sjöberg	1980													•							
Smiley-Oyen	2008			•																	
Smith	2009								•												
Smolarek	2016					•															
Sng	2017										•										
Sofianidis	2009				•																
Sofianidis	2018				•							•									
Stones	1993										•			•							
Stroth	md													•							
Sugano	2012						•														
Sungkarat	2016																			•	
Sungkarat	2018			•														•			
Suzuki	2012			•																	
Talwadkar	2014		•																		
Tarazona-Santaba	2016						•														
Taylor-Pilie	2010						•	•									•	•			
Teixeira	2013															•					
Timmons	2018					•															
Tomporowski	1987													•							
Tomporowski	2003													•							
Tomporowski	2005													•							
Tottori	2019																				•
Trombetti	2011											•									
Tsai	2017			•													•				
Tsourlou	2006											•									
Tsutsumi	1997						•														
van de Rest	2014							•													
van Dongen	2016										•										
van Uffelen	2008			•																	
Varas-Diaz	2020											•									
Varela	2012						•														
Vaughan	2014			•			•	•												•	
Venturelli	2011																		•		
Villareal	2011						•														
Voss	2006														•						
Voss	2010																•				
Voss	2009																•				

Author	Year	Angevaren et al (7) 2008	Bhattacharyya et al (9) 2020	Chen et al (10) 2020	Clifford et al (48) 2023	Coelho-Junior et al (8) 2022	Falck et al (40) 2019	Gasquoine and Chen (2022) 2022	Hindin and Zelinski (51) 2012	Jiang et al (49) 2022	Loprinzi et al (45) 2019	Ma et al (13) 2023	Martins et al (50) 2022	Roig et al (14) 2013	Scherder et al (52) 2014	Wang et al (46) 2021	Xiong et al (39) 2020	Ye et al (32) 2021	Zhao et al (53) 2022	Zhidong et al (55) 2021	Zhu et al (54) 2023
Wade	2018										•										
Wang	2021											•									
Weinberg	2014										•										
Wells	2013		•																		
Whitehurst	1991	•					•														
Wilke	2020																				•
Williams	1997						•		•												
Williamson	2009						•	•	•										•		
Winter	2007													•							
Woloszyn	2021											•									
Wu	2021																				•
Yanes	2018										•										
Yang	2019																			•	•
Yoon	2016						•														
Yoon	2017												•								
Yoon	2018												•							•	
Zhang	2021																				•
Zhang	2021																				•
Zhu	2018																			•	
Zuniga	2018										•										
