# Supplementary material for: Mind body exercise improves cognitive function more than aerobic- and resistance exercise in healthy adults aged 55 years and older – an umbrella review
Source: Eur Rev Aging Phys Act. 2023 Aug 9;20:15. doi: 10.1186/s11556-023-00325-4 (PMC10413530; doi:10.1186/s11556-023-00325-4)
Supplement: Supplementary file 4 — Additional file 4: Supplement 4. Cognitive domains and tests. [file 11556_2023_325_MOESM4_ESM.pdf]

Supplement 4. Cognitive domains and tests

Cognitive domain	Cognitive tests
Global cognitive function	Cambridge brain sciences computerized cognitive battery<br>Cambridge cognitive examination<br>Mini mental state examination<br>Montreal cognitive assessment
Executive function	Auditory verbal learning test<br>Clock drawing test<br>Control oral word association test<br>Color Trails<br>Digit-letter task<br>Digit-Span<br>Digit-Symbol<br>Dimension-switch task<br>Executive control<br>Executive interview 25<br>Flanker task<br>Floor Maze<br>Go and no go<br>Groton Maze Learning<br>Hayling task<br>Letter-number sequencing<br>List learning delayed recall test<br>N-back<br>Madrid card sorting test<br>N-back<br>Oculomotor assessment<br>Plus-minus task<br>Random number generation<br>Raven<br>Response inhibition<br>Rey osterrieth complex figure test<br>Ross information processing s progressive matrices test<br>Set-shifting<br>Spatial working memory task<br>Stopping test<br>Stroop<br>Stroop color word test<br>Task switching<br>Trail making test A<br>Trail making test B<br>Verbal digit span
Memory	2-back task<br>10-minute film<br>10-word list learning task<br>16 words immediate recall<br>16 words delayed recall<br>40-item word list<br>60s animal-naming test<br>180 images from IAPS

	III letter-number sequencing<br>Associative vocabulary learning task<br>Auditory verbal learning test<br>Backward digit span<br>Benton visual retention test<br>Brown Peterson test<br>Brown Poulton test<br>California verbal learning test<br>Category verbal fluency test<br>Clock drawing<br>CNS vital signs<br>Code substitution task<br>Cognitive neuropsychological evaluation<br>Complex figure test<br>Continual processing task<br>Corsi block-tapping<br>Delay recall<br>Digit span backwards subtest<br>Digit span numbers<br>Digit span task<br>Digit symbol substitution test<br>Face-name matching task<br>Forward digit-span test<br>Hopkins verbal learning test<br>Image recognition<br>Immediate and delayed free recall<br>Immediate word recall<br>ImPACT immediate recall verbal memory task<br>ImPACT immediate recall visual memory task<br>Letter memory<br>Letter-number sequencing<br>Letter search task<br>Letter verbal fluency<br>Logical memory delayed recall<br>Logical memory-Wechsler memory scale<br>Mattis dementia rating scale-memory<br>Mean repetition gap<br>Mental control test of Wechsler memory scale<br>Modified California verbal learning test<br>Modified Sternberg task<br>MQ of Wechsler memory scale<br>N-back, keep track<br>Operation span test<br>Paced auditory serial addition task<br>Pair associate learning task<br>Paired- associates memory task<br>Paragraph recalls<br>Picture memory<br>Random number generation task<br>Randt memory test story recall<br>Reading span test<br>Recall of visually observed film stimuli<br>Recall of words<br>Rey 15-item memory test<br>Rey auditory verbal learning delayed recall trial

	Rey auditory verbal learning task<br>Rey auditory verbal learning trial I-V<br>Ross information processing assessment immediate memory<br>Running memory span<br>Self-ordered pointing task<br>Six letter cancellation test<br>Spatial span<br>Spatial working memory<br>Subjective cognitive complaints<br>The NIH toolbox<br>Trail making test B/A<br>Verbal fluency test<br>Verbal memory test<br>Verbal memory free recall task<br>Visual memory test<br>Visual span<br>Visual spatial memory test<br>Wechsler adult intelligence scales digit span<br>Wechsler adult intelligence scales letter number sequencing<br>Wechsler adult intelligence scales logical memory immediate recall<br>Wechsler memory scales logical memory subtest<br>Wechsler memory scales memory quotient<br>Wechsler memory scales visual reproduction<br>Word fluency<br>Verbal fluency test<br>Wechsler memory scales, mental control<br>Wisconsin card sorting test<br>Word comparison
Attention	2&7 test<br>Digit span forward<br>Digit vigilance<br>Finger tapping<br>Letter search primary task RT<br>Stroop test<br>Toulouse-Pieron´s concentration attention test<br>Tracking<br>Visual search, accuracy
Processing speed	Verbal fluency test<br>Wechsler memory scales, mental control<br>Wisconsin card sorting test<br>Word comparison<br>Digit symbol substitution

Specific cognitive domains and tests used in the studies. Note that some tests appear under more than one domain.
