# Supplementary figures and images for: Mind body exercise improves cognitive function more than aerobic- and resistance exercise in healthy adults aged 55 years and older – an umbrella review
Source: Eur Rev Aging Phys Act. 2023 Aug 9;20:15. doi: 10.1186/s11556-023-00325-4 (PMC10413530; doi:10.1186/s11556-023-00325-4)

## Supplement S5. Funnel plots for subgroups

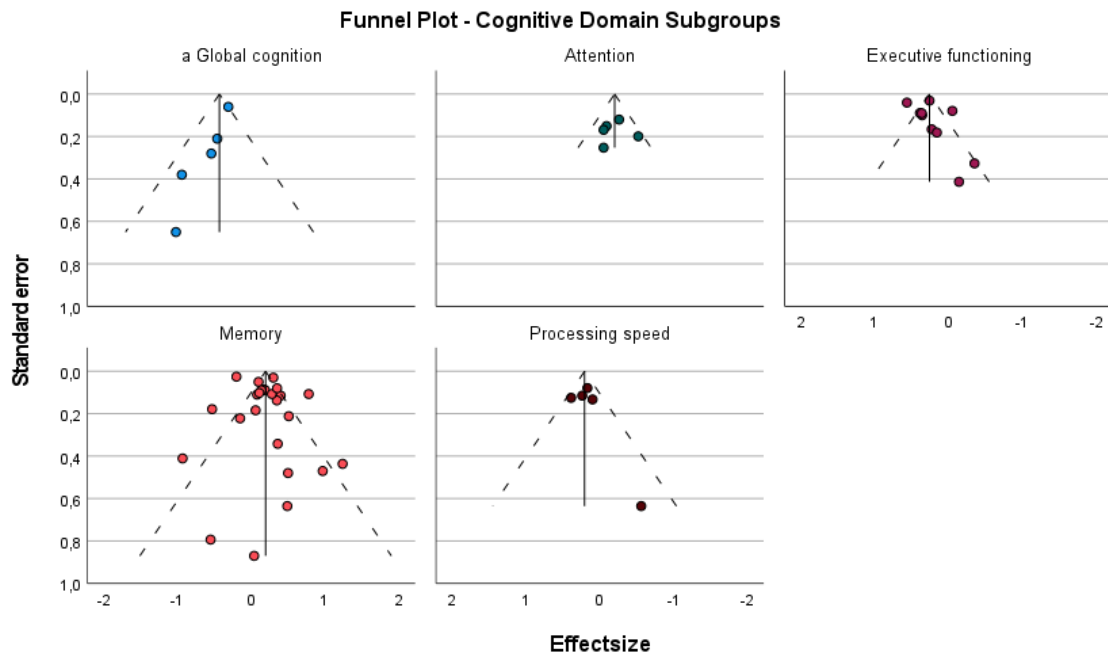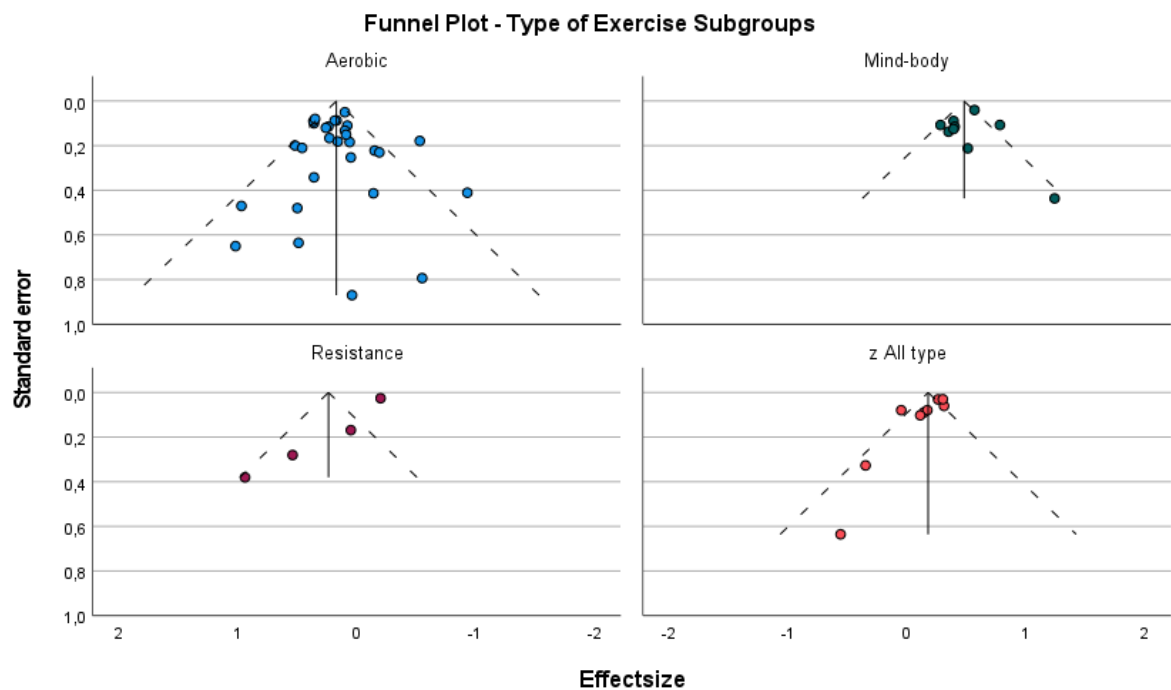

Funnel Plot - Acute and Chronic exercise

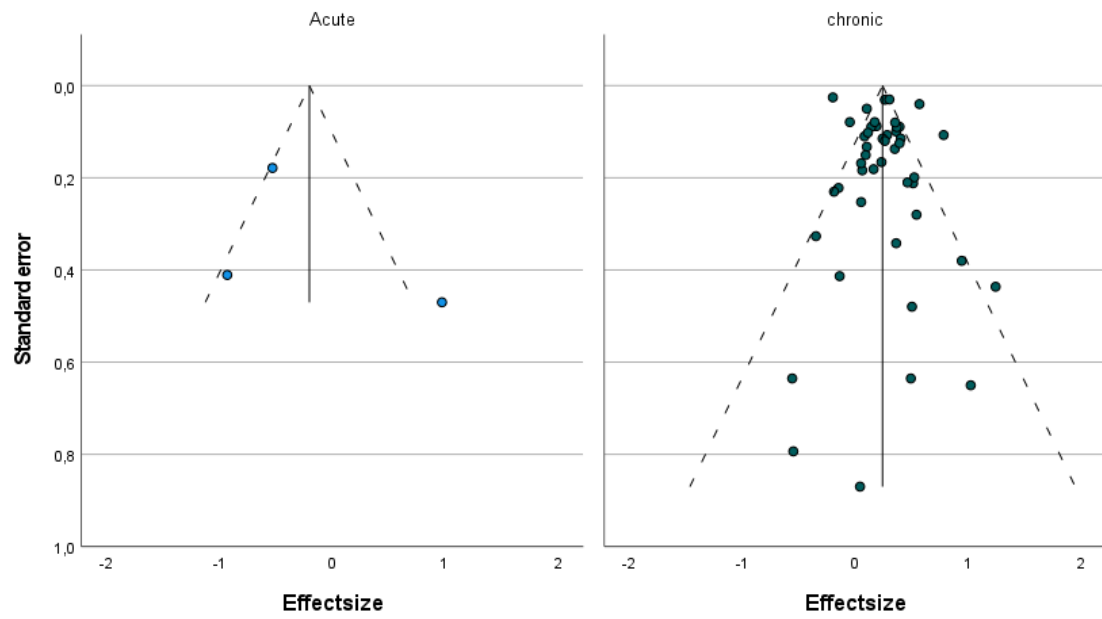

Supplement: Supplementary file 5 — Additional file 5: Supplement S5. Funnel plots for subgroups. [file 11556_2023_325_MOESM5_ESM.pdf]
