# Supplementary material for: Mind body exercise improves cognitive function more than aerobic- and resistance exercise in healthy adults aged 55 years and older – an umbrella review
Source: Eur Rev Aging Phys Act. 2023 Aug 9;20:15. doi: 10.1186/s11556-023-00325-4 (PMC10413530; doi:10.1186/s11556-023-00325-4)
Supplement: Supplementary file 6 — Additional file 6: Supplement S6. Type of exercise stratified by cognitive domain. [file 11556_2023_325_MOESM6_ESM.pdf]

Supplement S6. Type of exercise stratified by cognitive domain

						95% CI		
**Domain**	**Exercise type**	**effect size**	**std error**	**Z**	**Sig (2-tailed)**	**Lower**	**Upper**	**# reviews**
Executive function	Aerobic	0,08	0,08	1,01	0,31	-0,08	0,24	5
	Mind-body	0,50	0,10	4,93	<,001	0,30	0,70	3
	Resistance	-0,20	0,03	-7,84	<,001	-0,25	-0,15	1
	Mixed	0,21	0,07	3,14	0,00	0,08	0,34	2
	Overall	*0,20*	*0,07*	*2,90*	*0,00*	*0,06*	*0,33*	
Processing speed	Aerobic	0,18	0,09	2,07	0,04	0,01	0,35	1
	Mind-body	0,39	0,13	3,12	0,00	0,15	0,63	1
	All type	0,08	0,24	0,32	0,75	-0,40	0,55	1
	Overall	*0,21*	*0,05*	*3,94*	*0,00*	*0,10*	*0,31*	
Attention	Aerobic	0,23	0,08	2,89	0,00	0,08	0,39	1
	Resistance	0,05	0,17	0,30	0,77	-0,28	0,38	1
	Overall	*0,20*	*0,07*	*2,74*	*0,01*	*0,06*	*0,34*	
Global Cognition	Aerobic	0,51	0,20	2,57	0,01	0,12	0,90	2
	Resistance	0,68	0,23	3,02	0,00	0,24	1,12	2
	z All type	0,31	0,06	5,17	0,00	0,19	0,43	1
	Overall	*0,43*	*0,11*	*4,02*	*0,00*	*0,22*	*0,64*	
