# Supplementary material for: Mind body exercise improves cognitive function more than aerobic- and resistance exercise in healthy adults aged 55 years and older – an umbrella review
Source: Eur Rev Aging Phys Act. 2023 Aug 9;20:15. doi: 10.1186/s11556-023-00325-4 (PMC10413530; doi:10.1186/s11556-023-00325-4)
Supplement: Supplementary file 7 — Additional file 7: Supplement S7. A) Sensitivity analysis showing effect size when each study is individually removed from the analysis. [file 11556_2023_325_MOESM7_ESM.pdf]

Supplement S7. A) Sensitivity analysis showing effect size when each study is individually removed from the analysis

Study removed	Effect size	Std error	Z	Sig	95%CI (Lower)	95%CI (Upper)
**Total**	**.224**	**.0377**	**5.932**	**<.001**	**.150**	**.298**
Angevaren et al. 2008 (7)	.239	.0501	4.774	<.001	.141	.337
Bhattacharyya et al. 2021 (9)	.209	.0401	5.212	<.001	.130	.288
Chen et al. 2020 (10)	.222	.0390	5.694	<.001	.146	.299
Clifford et al. 2022 (48)	.230	.0377	6.101	<.001	.156	.304
Coelho-Junior et al. 2022 (8)	.240	.0360	6.665	<.001	.169	.311
Falk et al. 2019 (40)	.221	.0389	5.680	<.001	.145	.297
Gasquoine and Chen. 2022 (41)	.250	.0412	6.067	<.001	.169	.331
Jiang et al. 2022 (49)	.219	.0386	5.683	<.001	.144	.295
Loprinzi et al. 2019 (45)	.245	.0347	7.063	<.001	.177	.313
Ma et al. 2023 (13)	.220	.0381	5.759	<.001	.145	.294
Martins et al. 2022 (50)	.218	.0376	5.811	<.001	.145	.292
Roig et al. 2013 (14)	.227	.0397	5.731	<.001	.150	.305
Scherder et al. 2014 (52)	.220	.0386	5.691	<.001	.144	.295
Wang et al. 2021 (46)	.226	.0379	5.970	<.001	.152	.300
Xiong et al. 2021 (39)	.210	.0390	5.368	<.001	.133	.286
Ye et al. 2021 (32)	.196	.0360	5.435	<.001	.125	.266
Zhao et al. 2022 (53)	.221	.0377	5.863	<.001	.147	.295
Zhidong et al. 2021 (55)	.221	.0390	5.670	<.001	.145	.297
Zhu et al. 2023 (534	.220	.0387	5.679	<.001	.144	.296

B) Sensitivity analysis showing effect size when studies including acute exercise interventions were removed.

Study removed	Effect size	Std error	Z	Sig	95%CI (Lower)	95%CI (Upper)
**Total**	**.224**	**.0377**	**5.932**	**<.001**	**.150**	**.298**
Studies with acute exercise interventions removed	.240	.0358	6.690	<.001	.169	.31
